# Phanogracilins A–C, New Bibenzochromenones of Crinoid *Phanogenia gracilis* (Hartlaub, 1890)

**DOI:** 10.3390/biom14020151

**Published:** 2024-01-26

**Authors:** Elena A. Vasileva, Dmitrii V. Berdyshev, Natalia P. Mishchenko, Andrey V. Gerasimenko, Ekaterina S. Menchinskaya, Evgeniy A. Pislyagin, Ekaterina A. Chingizova, Leonid A. Kaluzhskiy, Salim Sh. Dautov, Sergey A. Fedoreyev

**Affiliations:** 1 G.B. Elyakov Pacific Institute of Bioorganic Chemistry, Far Eastern Branch of the Russian Academy of Sciences, Vladivostok 690022, Russia; berdyshev@piboc.dvo.ru (D.V.B.); mischenkonp@mail.ru (N.P.M.); ekaterinamenchinskaya@gmail.com (E.S.M.); pislyagin@hotmail.com (E.A.P.); martyyas@mail.ru (E.A.C.); fedoreev-s@mail.ru (S.A.F.); 2 Institute of Chemistry, Far Eastern Branch of the Russian Academy of Sciences, Vladivostok 690022, Russia; gerasimenko@ich.dvo.ru; 3 Institute of Biomedical Chemistry, Moscow 119121, Russia; leonid.kaluzhskiy@ibmc.msk.ru; 4 A.V. Zhirmunsky National Scientific Center of Marine Biology, Far Eastern Branch of the Russian Academy of Sciences, Vladivostok 690041, Russia; daut49shakir@mail.ru

**Keywords:** echinoderms, crinoids, benzochromenones, dimers, antioxidants, neuroprotectors, antimicrobials, biofilms

## Abstract

Three new bibenzochromenones named phanogracilins A–C (**1**–**3**) were isolated from the crinoid *Phanogenia gracilis*. The structure of **1** was established using X-ray crystallography as 5,5′,6,6′,8,8′-hexahydroxy-2,2′-dipropyl-4H,4′H-[7,9′-bibenzo[g]chromene]-4,4′-dione. This allowed us to assign reliably 2D NMR signals for compound **1** and subsequently for its isomer **2** that differed in the connecting position of two benzochromenone moieties (7,10′ instead of 7,9′), and compound for **3** that differed in the length of the aliphatic chain of one of the fragments. Compound **4** was derived from **1** in alkaline conditions, and its structure was elucidated as 5,5′,6′,8,8′-pentahydroxy-2,2′-dipropyl-4H,4′H-[7,9′-bibenzo[g]chromene]-4,4′,6,9-tetraone. Even though compounds **1**–**4** did not contain stereo centers, they possessed notable optical activity due to sterical hindrances, which limited the internal rotation of two benzochromenone fragments around C(7)–C(9′/10′) bonds. Isolated bibenzochromenones **1**–**4** were tested for their antiradical, neuroprotective and antimicrobial activities. Compounds **1**, **3** and **4** demonstrated significant antiradical properties towards ABTS radicals higher than the positive control trolox. Compounds **1** and **4** exhibited moderate neuroprotective activity, increasing the viability of rotenone-treated Neuro-2a cells at a concentration of 1 µM by 9.8% and 11.8%, respectively. Compounds **1** and **3** at concentrations from 25 to 100 μM dose-dependently inhibited the growth of Gram-positive bacteria *S. aureus* and yeast-like fungi *C. albicans*, and they also prevented the formation of their biofilms. Compounds **2** and **4** exhibited low antimicrobial activity.

## 1. Introduction

Crinoids are one of the most ancient echinoderm groups. The bright colors of crinoids have attracted the attention of investigators since the Challenger scientific expedition in the 1870s. These are mainly explained by the production of specific secondary metabolites—heavily oxidized quinones of polyketide origin [1]. To date, around 60 compounds of quinonoid nature have been isolated from crinoids [1,2,3,4,5,6,7], and most of them are substituted 9,10-anthraquinones, bisanthrones, phenantroperylenes, naphthopyrones and benzochromenes.

It has been assumed that sulfated quinonoid pigments have a defensive function against predators [8], as was confirmed by Takahashi et al., who showed that ptilometric acid 6-*O*-sulfate (1,6,8-trihydroxy-3-propyl-9,10-anthraquinone-2-carboxy-6-*O*-sulfate) exhibited concentration-dependent antifeedant activity towards freshwater fish (*Poecilia reticulata*) and marine fish (*Oplegnathus fasciatus*, *Parapristipomatriline atum*), but ptilometric acid was ineffective [9]. It was established that a series of sulfated naphthopyrones and anthraquinones from *Comanthus delicata* exhibited cytotoxicity to varying degrees against LNCaP (prostate cancer) and SK-Mel-2 (melanoma) cell lines [7]. Meanwhile, 9,10-anthraquinone derivatives rhodoptilometrin and crinemodin isolated from *Colobometra perspinosa* showed moderate, non-selective activity against three human tumor cell lines: MCF-7 (breast pleural-effusion adenocarcinoma), SF-268 (CNS-glioblastoma) and H460 (lung large-cell carcinoma) [10].

Naphthopyrone 6-methoxycomaparvin and its 5-methyl ether isolated from *Comanthus parvicirrus* completely inhibited TNF-α-induced NF-*κ*B activation and NF-*κ*B-DNA binding at an MIC of 300 μM [11]. Moreover, 6-methoxycomaparvin-5-methyl ether also exhibited a potent anti-inflammatory effect with 83.74% iNOS inhibition in LPS-stimulated RAW264.7 cells [5], as well as (+)-rhodoptilometrin and 3-propyl-1,6,8-trihydroxy-9,10-anthraquinone isolated from *Himerometra magnipinna* [12]. Capillasterquinone A (3-(2-one-n-pentyl)-1,6,8-trihydroxy-9,10-anthraquinone) reduced the LPS-induced iNOS and COX-2 expressions in a dose-dependent manner in RAW264.7 cells [13].

Seven benzo[*g*]chromen-4-one and benzo[*h*]chromen-4-one pigments of *Comantheria rotula* including the unusual benzo[*g*]chromenone dimer 9,9′-oxybis-neocomantherin significantly inhibited both hypoxia-induced and iron-chelator-induced HIF-1 luciferase reporter activity in breast and prostate tumor cells [14].

Crinemodin–rhodoptilometrin bianthrone isolated from *Himerometra magnipinna* slightly inhibited hyphae formation of a *Streptomyces* strain [15].

Gymnochrome D and isogymnochrome D isolated from the living fossil crinoid *Gymnocrinus richeri* were highly potent dengue antiviral agents at concentrations of <1 µM [16,17]. Naphthopyrones of *Capillaster multiradiatus* including comaparvin, 6-methoxycomaparvin-5-methyl ether and others inhibited in vitro HIV-1 replication in a T-cell line with EC_50_ values ranging from 7.5 to 25.5 µM without concomitant cytotoxicity [2].

Based on the above mentioned findings, the investigation of crinoid secondary metabolites of quinonoid nature remains pressing in the search for novel chemical structures with pharmacological potential.

In this article, we report the isolation and structural elucidation of three novel bibenzochromenones of crinoid *Phanogenia gracilis* not investigated before, along with one derivative obtained in the process of isolation, as well as certain biological activities of the isolated compounds.

## 2. Materials and Methods

### 2.1. General Experimental Procedures

Infrared (IR) spectra were obtained on an Equinox 55 Fourier-transform (FT) IR spectrophotometer (Bruker, Rheinstetten, Germany). The CD spectra were obtained on a Chirascan-plus Quick Start CD Spectrometer (Applied Photophysics Limited, Leatherhead, UK) (acetonitrile, 20 °C). The ^1^H-, ^13^C- and two-dimensional (2D) NMR spectra were recorded in DMSO-*d*_6_ using an NMR Bruker AVANCE DRX-500 instrument (Bruker, Karlsruhe, Germany). The chemical shift values (*δ*) and the coupling constants (*J*) are given in parts per million and Hz, respectively. HMBC spectra were optimized for 5 Hz coupling. HRESIMS spectra were obtained on a Bruker Maxis Impact II instrument (Bruker Daltonics, Bremen, Germany).

### 2.2. HPLC-DAD-MS Analysis

HPLC-DAD-MS was performed using a CBM-20A system controller (Shimadzu USA Manufacturing Inc., Canby, OR, USA), two LC-20 CE pumps (Shimadzu USA Manufacturing Inc., Canby, OR, USA), a DGU-20A3 degasser (Shimadzu Corp., Kyoto, Japan), an SIL-20A autosampler (Shimadzu USA Manufacturing Inc., Canby, OR, USA), a diode-matrix SPD-M20A (Shimadzu USA Manufacturing Inc., Canby, OR, USA) and mass-spectrometric detector LCMS-2020 (Shimadzu Corp., Kyoto, Japan). Separation was carried out on a Discovery HS C18 column (150 × 2.1 mm, 3 µm particle size, Supelco, Bellefonte, PA, USA) with a Supelguard Ascentis C18 pre-column (2 × 2.1 mm, 3 µm particle size, Supelco, Bellefonte, PA, USA) using a binary gradient of H_2_O (A): MeCN (B) with the addition of 0.2% AcOH, at a flow rate of 0.2 mL/min and column temperature of 40 °C. The gradient was as follows: 0–6 min, 30–100% (B); 6–14 min, 100% (B); 14–16 min, 100–30% (B), 16–20 min, 30% (B). The chromatograms were recorded at 254 nm. Mass spectra were taken in ESI mode at atmospheric pressure, recording negative ions (1.50 kV) in the m/z range of 100–900, with N_2_ as the drying gas (10 L/min) and N_2_ nebulizer gas flow (1.5 L/min), the temperature for the curved desolvation line (CDL) at 200 °C and for the heat block at 250 °C, and an interface voltage of 3.5 kV. Prior to analysis, samples were filtered through a 0.2 µm PTFE syringe filter. The injection volume was 3 µL.

### 2.3. Animal Material

A sample of crinoid was collected in the South China Sea near Ly Son Island (15°23′6 N; 109°06′09 E) by scuba divers (depth 3–9 m) during the 63rd scientific cruise of R/V Academic Oparin in May 2021. The species of crinoid was identified by Dr. S.Sh. Dautov, senior researcher at the Laboratory of Embryology of A.V. Zhirmunsky National Scientific Center of Marine Biology, Vladivostok, Russia, as *Phanogenia gracilis* (Hartlaub, 1890). A voucher sample was deposited in the collection of the Laboratory of Chemistry of Natural Quinonoid Compounds, G.B. Elyakov Pacific Institute of Bioorganic Chemistry, Vladivostok, Russia (no. 063-V32). After collection, the crinoid was rinsed with tap water, frozen whole and stored in a −20 °C freezer prior to extraction.

### 2.4. Extraction and Isolation

The defrosted *P. gracilis* (58.5 g) was first extracted with ethanol and then with 70% ethanol containing 10% H_2_SO_4_ at room temperature for 4 h. EtOH and acidified EtOH extracts were concentrated in vacuo at 50 °C. The residues were diluted with distilled water and subsequently extracted with chloroform and ethyl acetate. After evaporation of the solvents, the chloroform and ethyl acetate fractions were stored in a freezer at −20 °C prior to HPLC-DAD-MS analysis. Chloroform and ethyl acetate fractions obtained from EtOH extract with no addition of acid contained only traces of quinonoid pigments according to HPLC-DAD-MS data, so they were not used further.

Combined chloroform and ethyl acetate fractions (0.8 g) obtained from acidified EtOH extract were chromatographed repeatedly over a silica gel column (40–63 µm, Sigma-Aldrich, St. Louis, MO, USA), where silica gel was preliminary impregnated using a few drops of 5 mg/mL oxalic acid in ethanol. The column was eluted with a hexane–CHCl_3_ solution system with gradually increasing CHCl_3_ amounts (hexane/CHCl_3_, 1:0, 3:1, 2:1, 1:1, 1:2, 1:3, 1:4, 1:0 *v*/*v*) and then with CHCl_3_–MeOH with gradually increasing MeOH amounts (CHCl_3_/MeOH, 100:1, 50:1, 50:3, 10:1, 9:1, 5:1, 1:1, 1:0 *v*/*v*) to yield compounds **1** (32 mg), **2** (2.7 mg) and **3** (1.5 mg).

For further crystallization of compound **1** for X-ray analysis, we attempted to purify the compound with a simple method traditional forquinones [18]. Compound **1** (20 mg) was dissolved in CHCl_3_ and partitioned with 3% Na_2_CO_3_ in a separation funnel. The Na_2_CO_3_ aqueous phase containing compound **1** sodium salts was collected, while the CHCl_3_ layer was discarded. The aqueous phase was acidified to pH 2–3 and then partitioned with CHCl_3_. The CHCl_3_ layer was collected and evaporated in vacuo. HPLC-DAD-MS analysis showed that after this procedure, compound **4** was derived from **1**. Compound **4** (7.8 mg) was isolated after repeated chromatography of mixture of **1** and **4** on a silica gel column as described above.

Phanogracilin A (**1**), 5,5′,6,6′,8,8′-hexahydroxy-2,2′-dipropyl-4H,4′H-[7,9′-bibenzo[g]chromene]-4,4′-dione, C_32_H_26_O_10_: dark-red crystals (CHCl_3_); UV (CH_3_CN) λ_max_ 227, 253, 288, 411; CD (*c* 9.7 × 10^−5^, CH_3_CN) λ_max_ (Δε) 192 (+6.276); 211 (−2.890); 219 (−3.334); 271 (+2.343); 292 (−2.912); 317 (−0.336); IR (CDCl_3_) 1508, 1590, 1619, 1647, 2877, 2936, 2969, 3372, 3516; ^1^H and ^13^C NMR data, see Table 1; HR-ESI-MS *m*/*z* 571.1599 [M + H]^+^ (calcd. for C_32_H_27_O_10_ 571.1599).

Phanogracilin B (**2**), 5,5′,6,6′,8,8′-hexahydroxy-2,2′-dipropyl-4H,4′H-[7,10′-bibenzo[g]chromene]-4,4′-dione, C_32_H_26_O_10_: orange amorphous solids; UV (CH_3_CN) λ_max_ 227, 252, 282, 413; CD (*c* 1.4 × 10^−4^, CH_3_CN) λ_max_ (Δε) 197 (+3.312); 228 (−15.207); 249 (−3.587); 275 (+49.428); 291 (−35.339); 404 (+2.092); ^1^H and ^13^C NMR data, see Table 1; HR-ESI-MS *m*/*z* 571.1598 [M + H]^+^ (calcd. for C_32_H_27_O_10_ 571.1599).

Phanogracilin C (**3**), 5,5′,6,6′,8,8′-hexahydroxy-2′-methyl-2-propyl-4H,4′H-[7,9′-bibenzo[g]chromene]-4,4′-dione, C_30_H_22_O_10_: orange amorphous solids; UV (CH_3_CN) λ_max_ 227, 252, 286, 410; CD (*c* 1.3 × 10^−4^, CH_3_CN) λ_max_ (Δε) 221 (-2.384); 233 (+12.104); 272 (+85.919); 292 (-104.083); ^1^H and ^13^C NMR data, see Table 1; HR-ESI-MS *m*/*z* 543.1289 [M + H]^+^ (calcd. for C_30_H_23_O_10_ 543.1286).

5,5′,6′,8,8′-pentahydroxy-2,2′-dipropyl-4H,4′H-[7,9′-bibenzo[g]chromene]-4,4′,6,9-tetraone (**4**), C_32_H_24_O_11_: dark green amorphous solids; UV (CH_3_CN) λ_max_ 227, 246, 281, 347, 412; CD (*c* 2.5 × 10^−4^, CH_3_CN) λ_max_ (Δε) 194 (+3.141); 221 (−1.230); 247 (+0.262); 271 (+0.805); 293 (−0.922); ^1^H and ^13^C NMR data, see Table 1; HR-ESI-MS *m*/*z* 585.1390 [M + H]^+^ (calcd. for C_32_H_25_O_11_ 585.1391).

### 2.5. X-ray Experiment

Experimental intensity data for **1** were collected at T = 120(2) K on a BRUKER Kappa APEX2 diffractometer with graphite monochromated Mo K_α_ radiation (λ = 0.71073 Å). Intensity data were corrected for absorption using the multi-scan method. The structure was determined using direct methods and refined via least-squares calculation in anisotropic approximation for non-hydrogen atoms. Hydrogen atoms were placed in geometrically idealized positions and refined in the riding-model approximation. Data collection, reduction and refinement of the lattice parameters were performed using the Apex2 software package [19]. All calculations were performed with the SHELXL/PC program [20,21]. The main crystallographic data, details of refinement of the crystal structure of **1** and selected bond lengths are shown in Tables S1–S3.

Supplementary crystallographic data (accession number CCDC 2321657) can be obtained free of charge from the Cambridge Crystallographic Data Center via http://www.ccdc.cam.ac.uk/data_request/cif (accessed on 25 December 2023) or from the Cambridge Crystallographic Data Centre, 12 Union Road, Cambridge, UK; fax: +44-1223-336-033 or email: deposit@ccdc.cam.uk.

### 2.6. Quantum-Chemical Modeling

Conformational analysis was performed via density functional theory (DFT) with a B3LYP exchange-correlation functional, 6–311 G(d) basis set and polarizable continuum model (PCM), used for description of the solute–solvent interaction. All calculations were performed with the Gaussian 16 package of programs using default algorithms [22]. The statistical weights of individual conformations were calculated as follows:

(1) gim=e−ΔGim/RT∑ie−ΔGim/RT

where the summation was carried out over stable conformations of **1**–**4**, for which Δ*G_im_* ≤ 5 kcal/mol; the subscript “*m*” denotes conformation, for which *G* is minimal.

The UV and ECD spectra were calculated via time-dependent density functional theory (TDDFT) using the B3LYP/6–311 G(d)_PCM//B3LYP/6–311 G(d)_PCM calculation scheme. Individual bands in ECD spectra were modeled as Gauss-type functions with the bandwidths ζ = 0.28 eV. To effectively describe the high-frequency regions in ECD spectra (λ ≤ 240 nm), the total number of calculated vertical electronic transitions was taken to be n = 95. No UV shifts were used to achieve the best coincidence of the “reference” bands in calculated and experimental spectra.

### 2.7. ABTS Assay

We added 100 μL of 60 mM potassium persulfate (K_2_S_2_O_8_) to 10 mL of 7 mM 2,2-azinobis(3-ethylbenzothiazoline-6-sulfonic acid) (ABTS, CDH Ltd., New Delhi, India). This reaction mixture was left overnight in the dark to obtain a dark blue ABTS^•+^ cation radical, which was further diluted with distilled water until the initial absorbance value of 0.7 ± 0.05 at 734 nm was reached.

The studied compounds were dissolved in dimethylsulfoxide and trolox (Sigma-Aldrich, St. Louis, MO, USA) in ethanol so that their final concentration after the addition to 3 mL of ABTS was 2−25 µg/mL. The decrease in absorbance was recorded using a UV 1800 spectrophotometer (Shimadzu USA Manufacturing Inc., Canby, OR, USA) at 0 min and after 6 min. Graphs of sample concentration versus absorbance reduction were then constructed. The results were expressed as IC_50_ (µg/mL, µM) and trolox equivalent antioxidant capacity (TEAC) values. TEAC was calculated as the ratio of trolox IC_50_, µM, to sample IC_50_, µM. All measurements were performed in triplicate.

### 2.8. Cell Viability Assay (MTT Method)

The murine neuroblastoma cell line Neuro-2a was purchased from ATCC (CCL-131). Cells were cultured in DMEM (Biolot, St. Petersburg, Russia) containing 10% fetal bovine serum (Biolot, St. Petersburg, Russia) and 1% penicillin/streptomycin (Biolot, St. Petersburg, Russia). Cells were incubated at 37 °C in a humidified atmosphere containing 5% (*v*/*v*) CO_2_.

Stocks solutions of substances were prepared in DMSO at a concentration of 10 mM. All tested compounds were added to the wells of the plates in a volume of 20 μL diluted with PBS in final concentrations of 0.01, 0.1, 1.0 and 10.0 μM (final DMSO concentration < 1%).

Neuro-2a cells (1 × 10^4^ cells/well) were incubated in a CO_2_ incubator for 24 h at 37 °C for adhesion. After that, 20 μL of test solutions were loaded in the cells and incubated for 24 h. After incubation, the medium with tested substances was replaced with 100 μL of fresh medium. Then, 10 μL of MTT (3-(4,5-dimethylthiazol-2-yl)-2,5-diphenyltetrazolium bromide) (Sigma-Aldrich, St. Louis, MO, USA) stock solution (5 mg/mL) was added to each well and the microplate was incubated for 4 h. After that, 100 μL of SDS-HCl solution (1 g SDS/10 mL dH_2_O/17 μL 6 N HCl) was added to each well followed by incubation for 18 h. The absorbance of the converted dye formazan was measured using a Multiskan FC microplate photometer (Thermo Scientific Inc., Beverly, MA, USA) at a wavelength of 570 nm [23]. All experiments were repeated in triplicate. Cytotoxic activity was expressed as the percent of cell viability.

### 2.9. Rotenone-Induced In Vitro Model of Parkinson’s Disease

After 24 h of adhesion, Neuro-2a cells (1 × 10^4^ cells/well) were treated with compounds at concentrations of 0.1–10 μM for 1 h, and after that, 10 μM of rotenone (Sigma-Aldrich, St. Louis, MO, USA) was added. Cells incubated without rotenone or with rotenone were used as a positive and negative controls, respectively. Cell viability was measured after 24 h using the MTT method. The results were presented as a percentage of positive control data.

### 2.10. Reactive Oxygen Species (ROS) Level Analysis in Rotenone-Treated Cells

After 24 h of adhesion, Neuro-2a cells (1 × 10^4^ cells/well) were incubated with compounds at concentrations of 0.1–10 µM for 1 h. Then, rotenone at a concentration of 10 µM was added in each well, and cells were incubated for 1 h. To study ROS formation, 20 µL of 2,7-dichlorodihydrofluorescein diacetate solution (H_2_DCF-DA, Molecular Probes, Eugene, OR, USA) was added to each well, such that the final concentration was 10 µM, and the microplate was incubated for an additional 30 min at 37 °C. The fluorescence intensity was measured using a high-speed plate reader PHERAstar FS (BMG Labtech, Ortenberg, Germany) at λ_ex_ = 485 nm and λ_em_ = 518 nm. The results were processed in MARS Data Analysis v. 3.01R2 (BMG Labtech, Ortenberg, Germany). The results were presented as percentages of positive control data.

### 2.11. Antimicrobial Activity

The yeast-like fungi *Candida albicans* KMM 455 and bacterial strains *Staphylococcus aureus* ATCC 21027 and *Escherichia coli* VKPM (B-7935) (Collection of Marine Microorganisms PIBOC FEB RAS) were cultured on solid-medium Mueller Hinton broth with agar (16.0 g/L) in a Petri dish at 37 °C for 24 h.

The assays were performed in 96-well microplates in appropriate Mueller Hinton broth. Each well contained 90 µL of bacterial or of a yeast-like fungi suspension (10^6^ CFU/mL). Then, 10 µL was added of a compound diluted at concentrations from 1.5 µM to 100.0 µM using twofold dilution (DMSO concentration < 1%). Culture plates were incubated overnight at 37 °C, and the OD_620_ was measured using a Multiskan FC spectrophotometer (Thermo Scientific Inc., Beverly, MA, USA). The antibiotic gentamicin and antifungal agent nitrofungin were used as positive controls at 1 mg/mL; 1% DMSO in PBS served as a negative control. Examination was performed twice and in triplicate. The results were calculated as a percentage of the control data in SigmaPlot 14.0 software.

### 2.12. Biofilm Formation

The inhibition of the biofilm formation was assessed using the MTT test, as described [24]. Mueller Hinton broth was inoculated with 10^9^ CFU/mL of microorganisms: *C. albicans*, *S. aureus* and *E. coli* overnight cultures. A total of 90 µL of this cell suspension was then dispensed into 96-well microtiter plates containing 10 µL of compound diluted at concentrations from 1.5 µM to 100.0 µM using twofold dilution (DMSO concentration < 1%). After 24 h growth at 37 °C, the plates were washed with PBS to remove unbound cells. Next, 10 µL of MTT solution in PBS (5 mg/mL, Sigma-Aldrich, Munich, Germany) was added to each well and incubated for 2–4 h. Then, the media was carefully aspirated and the plates were dried for 2 h. Then, 100 µL/well of DMSO was added to each well to dissolve formazan crystals, and the absorbance was measured using a plate reader according to the manufacture’s protocol. The results were reported as percent inhibition normalized to the wild-type control. The antibiotic gentamicin and antifungal agent nitrofungin were used as positive controls at 1 mg/mL; 1% DMSO in PBS served as a negative control. Examination was performed twice and in triplicate.

## 3. Results and Discussion

### 3.1. Structure Elucidation of Compounds ***1***–***4***

In most publications, crinoids were extracted with acetone, methanol or a mixture of methanol and dichloromethane, and compounds of quinonoid nature were successfully obtained [3,4,5,6,9,10,13,14,15,25,26,27]. Sometimes, ethanol [2,28] and water [7,29] were also used as extragents. Accordingly, Sutherland et al. suggested that pigments of crinoids are not bound as stable complexes with proteins and salts in the calcareous skeleton [30] as described for other echinoderms—echinoids and holothurians [31].

In our case, ethanol extracted only traces of colored pigments from the *P. gracilis* sample (Figures S1–S3). For *Comanthus parvicirrus timorensis*, it was described that treatment of the solvent-exhausted animals with dilute hydrochloric acid dissolved the calcareous skeleton and liberated a red substance, probably polyhydroxynaphthoquinones [32]. Hypericin and its derivatives occur in the fossil crinoid material as salts, providing an effective mechanism for chemical stabilization, and can be obtained through dissolution of the calcite with hydrochloric acid [33,34,35,36]. Considering this and our experience of work with quinonoid pigments of sea urchins [37], which can be extracted from shells only with acidic solutions, we extracted *P. gracilis* with 70% ethanol containing 10% H_2_SO_4_ and obtained a deep red solution with a green tint. Acidified EtOH extract was concentrated in vacuo, and the residue was subsequently extracted with chloroform and ethyl acetate. HPLC-DAD-MS analysis revealed that chloroform and ethyl acetate extracts contained the same pigments in slightly different ratios, which were in traces in ethanolic extract: two compounds with molecular masses 570 (**1**, **2**) and one compound with molecular mass 542 (**3**) (Figures S4–S11). All of them had the very similar absorption spectra typical for quinones with λ_max_ around 227, 252, 285 and 411 nm (Figures S6, S8 and S10).

**Figure 1:**
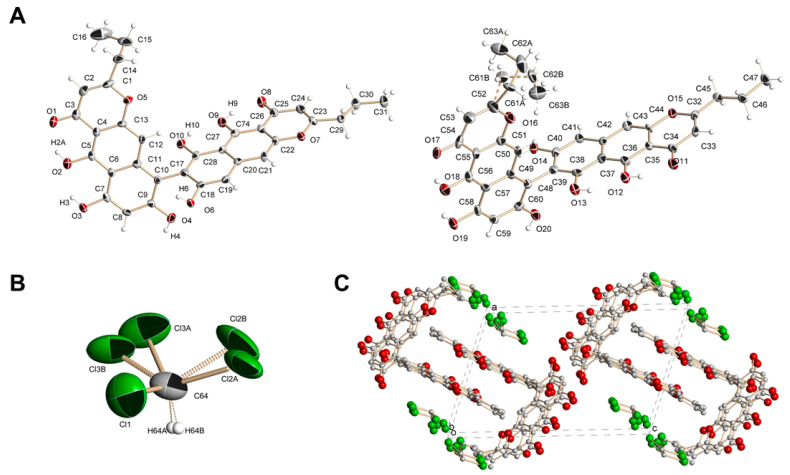
Structure of compound **1** determined via X-ray. (**A**). Crystallographically independent C_32_H_26_O_10_ molecules in the crystal of **1** (on the left is a molecule with a disordered propyl group). (**B**). Molecule of chloroform in the crystal of **1**. (**C**). Projection of crystal structure of **1** onto the *ac* plane.

Compound **1** was obtained as a dark red powder with the molecular formula C_32_H_26_O_10_, as deduced from analysis of the HRESIMS (*m*/*z* 571.1599 [M + H]^+^, calcd. for C_32_H_27_O_10_ 571.1599). The IR spectrum of **1** showed absorption bands indicating the presence of hydroxy (3516, 3372 cm^−1^) and keto (1647, 1619 cm^−1)^ groups. The ^1^H-NMR spectrum of compound **1** exhibited signals of two propyl chains, six aromatic protons, four hydroxyl protons and two bright signals in a weak field at around 16 ppm present only in spectrum recorded in acetone-*d*_6_ (Figures S16–S20), which might belong to two chelated hydroxyl protons (Table 1). The ^13^C-NMR spectrum of **1** revealed thirty-two carbon atoms (Table 1); two of these signals were assigned to carbonyl carbons (*δ*_C_ 184.21, 184.25), eight were assigned to oxygenated aromatic carbons (*δ*_C_ 152.65–163.53), twelve were assigned to the non-oxygenated aromatic carbons (*δ*_C_ 99.09–109.13), two were assigned to oxygenated aromatic carbons with aliphatic substituents (*δ*_C_ 173.42, 173.93) according to published data and six were assigned to the aliphatic carbons (*δ*_C_ 14.16–36.45) of two propyl chains. Based on the above observations, compound **1** was assumed to be a benzochromenone dimer. However, very close values of carbon atoms’ chemical shifts did not allow for accurate assignment of signals using HMBC data (Figures S21–S25). A suitable dark red prismatic crystal of **1** was obtained from a solution of chloroform. Using X-ray analysis, it was established that the crystal of **1** consisted of units containing two C_32_H_26_O_10_ molecules and one molecule of chloroform (Figure 1). Some of the chlorine atoms in the chloroform molecule and one of the propyl groups in the C_32_H_26_O_10_ molecule were equally likely disordered over two positions (A and B). The disordered atoms were refined with site occupancies of 0.5. Molecules of **1** were combined into a three-dimensional structure using O–H···O and C–H···O hydrogen bonds (Figure 1).

**Figure 2:**
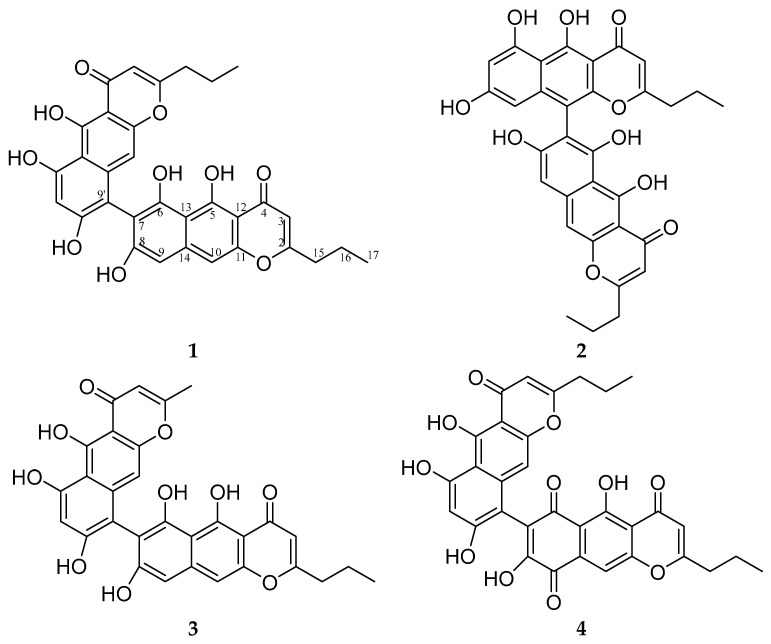
Structures of compounds **1**–**4**.

X-ray analysis revealed the structure of **1** as 5,5′,6,6′,8,8′-hexahydroxy-2,2′-dipropyl-4H,4′H-[7,9′-bibenzo[g]chromene]-4,4′-dione and allowed us to assign NMR signals more reliably. Compound **1** was named phanogracilin A (Figure 2).

Compound **2** was an isomer of compound **1** according to HRESIMS and had NMR data very similar to **1** (Table 1). ^1^H- and ^13^C-NMR data of one fragment of the dimer molecule **2** almost coincided with those of compound **1** (Figures S27–S34). The key difference in the ^1^H-NMR spectrum of the second fragment of compound **2** compared to **1** was the presence of two doublets at *δ*_H_ 6.19 and 6.30 ppm, with *J* = 2.2 Hz indicating the meta position of corresponding aromatic protons. Such a mutual arrangement of protons is possible only in the case of connection of two fragments between positions 7 and 10′, which was also supported by HMBC correlations, so the structure of compound **2** was established as 5,5′,6,6′,8,8′-hexahydroxy-2,2′-dipropyl-4H,4′H-[7,10′-bibenzo[g]chromene]-4,4′-dione and it was named phanogracilin B.

Compound **3** had an *m*/*z* 543.1289 [M + H]^+^ (calcd. for C_30_H_23_O_10_ 543.1286), which was 28 Da less than the mass of compound **1**. ^1^H- and ^13^C-NMR data of **3** were very similar to those of compound **1** (Figures S35–S42), except for a lack of signals of one propyl side chain, instead of which a signal of a methyl substituent was present (*δ*_H_ 2.27, *δ*_C_ 21.57, Table 1), which was consistent with the mass spectrometry data. So, based on HMBC correlations, the structure of compound **3** was established as 5,5′,6,6′,8,8′-hexahydroxy-2′-methyl-2-propyl-4H,4′H-[7,9′-bibenzo[g]chromene]-4,4′-dione and it was named phanogracilin C.

Compounds **1** and **2** could be considered dimers of neocomantherin [14] with demethylated hydroxy groups in positions 5 and 6, and compound **3** as a dimer of neocomantherin and comantherin [38] with demethylated hydroxy groups in the same positions. Benzochromenones are common crinoid metabolites; however, usually, they are methoxylated [1], so here arose the question of whether compound **1** and its relative compounds **2** and **3** isolated by us were native or were derived in acidic conditions during the isolation process.

We detected traces of these compounds in the ethanolic extract of *P. gracilis* with no addition of acid using HPLC-DAD-MS (Figure S1). Also, we knew from our experience of work with methoxylated quinones of sea urchins that they remain stable during the processes of isolation and purification in the conditions we used in this experiment [37]. Thus, we were inclined to consider the isolated compounds **1**–**3** to be natural and surmise that they were deposited in the *P. gracilis* skeleton.

It was interesting that only one crinoid dimeric benzochromenone was known before—9,9′-oxybis-neocomantherin (9,9′-oxybis(8-hydroxy-5,6-dimethoxy-2-propyl-4H-benzo[g]chromen-4-one), a dimeric neocomantherin connected through an oxygen bridge isolated from *Comantheria rotula* [14].

HPLC-DAD-MS analysis showed that after purification of **1** through Na_2_CO_3_ in a separation funnel, compound **4** was derived from **1** in alkaline conditions (Figures S43–S46). Compound **4** had an *m*/*z* 585.1390 [M + H]^+^ (calcd. for C_32_H_25_O_11_ 585.1391), which was 14 Da more than the mass of compound **1**. ^1^H- and ^13^C-NMR data of one fragment of molecule **4** almost coincided with those of compound **1** (Table 1, Figures S47–S54). The other fragment of compound **4** according to NMR spectra contained two carbonyl groups (*δ*_C_ 181.43, 186.5) located instead of one aromatic proton and hydroxy group, so compound **4** was assigned the structure 5,5′,6′,8,8′-pentahydroxy-2,2′-dipropyl-4H,4′H-[7,9′-bibenzo[g]chromene]-4,4′,6,9-tetraone.

### 3.2. Optical Activity of Compounds ***1***–***4***

**Figure 3:**
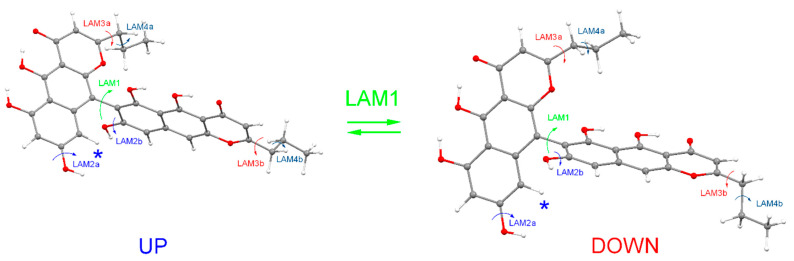
The types of large-amplitude motions (LAMs), which determine conformational rearrangements in the example of compound **2**. The asterisk “*” marks a hydrogen atom, the position of which generates the biggest sterical hindrances for LAM1 motion.

Compounds **1**–**4** belong to a rare class of compounds, which do not contain any stereo centers, but which, nevertheless, possess notable optical activity, called atropisomerism [39]. The reason for such unusual features of these compounds lies in sterical hindrances, which limit the internal rotation of two benzochromenone fragments around C(7)-C(9′/10′) bonds, thus preventing transformation of rotameric forms of the “UP” type to rotameric forms of the “DOWN” type (Figure 3).

According to our calculations, the heights of potential energy barriers for “UP” → “DOWN” transfers in **1**–**4** are ΔE^≠^ ≥ 50 kcal/mol, which means there are a very long lifetimes of “UP” and “DOWN” rotameric forms (τ ≥ 10^16^ years, the estimate was made without taking into account the tunneling effect). In addition to the “UP” → “DOWN” transfer, a number of other LAMs may occur—the prototropic tautomeric rearrangements and the internal rotations of the alkyl and hydroxyl groups. Each individual conformation of molecule—the specific rotameric form “UP” or “DOWN”, plus the specific tautomeric form, plus the specific orientation of the alkyl and hydroxyl substituents—is characterized by its own shape of ECD spectrum. Compared to the “UP” → “DOWN” transfer, all other conformational rearrangements proceed very quickly. As a result, the experimental ECD spectrum is a sum of averaged over quick processes spectra for “UP” and “DOWN” rotameric forms. The proportions in which “UP” and “DOWN” forms are present in the sample of the compound under study determine the signs and intensities of individual bands in the experimental ECD spectra of compounds **1**–**4**.

The total ECD spectrum of compounds **1**–**4** may be seen as a sum of ECD spectra generated by the pairs of mirror-reflected conformations:

(2) $$\begin{array}{ll} \Delta \varepsilon \left(\lambda \right) & =g_{up,\Sigma }\;\cdot \;\sum_{i}^{N}g_{i,up}\Delta \varepsilon_{i,up}\left(\lambda \right)+\;g_{down,\Sigma }\;\cdot \;\sum_{i}^{N}g_{i,up}\Delta \varepsilon_{i,up-mirror-reflected}\left(\lambda \right)\\ & =\left(g_{up,\Sigma }-g_{down,\Sigma }\right)\;\cdot \sum_{i=1}^{N}g_{i,up}\;\cdot \;\Delta \varepsilon_{i,up}\left(\lambda \right)=\left(g_{up,\Sigma }-g_{down,\Sigma }\right)\;\;\cdot \Delta \varepsilon_{up,\Sigma }\left(\lambda \right) \end{array}$$

where index “*i*” enumerates possible conformations for the “UP” rotameric form, “*g_up,∑_*” and “*g_down,_*_∑_” are the amounts of the “UP” and “DOWN” rotameric forms, “*g_i,up_*” is a statistical weight of the *i*-th conformation, calculated based on the assumption that 100% of the sample exclusively consists of the “UP” rotameric form; and index “*up-mirror-reflected*” designates the mirror-imaged counterpart of the “*i*-th” conformation of the “UP” rotameric form.

Hence, the interpretation of experimental ECD spectra can achieve two goals: (1) the determination of the qualitative levels of the relative amounts of “UP” and “DOWN” rotameric forms in the compound under study; (2) the determination of the absolute amounts of the “UP” and “DOWN” rotameric forms in the sample. Our study was devoted to conducting the first task. First, using density functional theory (DFT), we performed conformational analysis for compounds **1**–**4**. Based on this, the meaningful conformations were chosen for further calculations of ECD spectra (conformations with Gibbs free energies |*G_i_* − *G_i,main_*| ≤ 5 kcal/mol; “main” designates conformation, for which Gibbs free energy is minimal). The interaction of the solute with the solvent was modeled using a polarizable continuum model (PCM).

**Figure 4:**
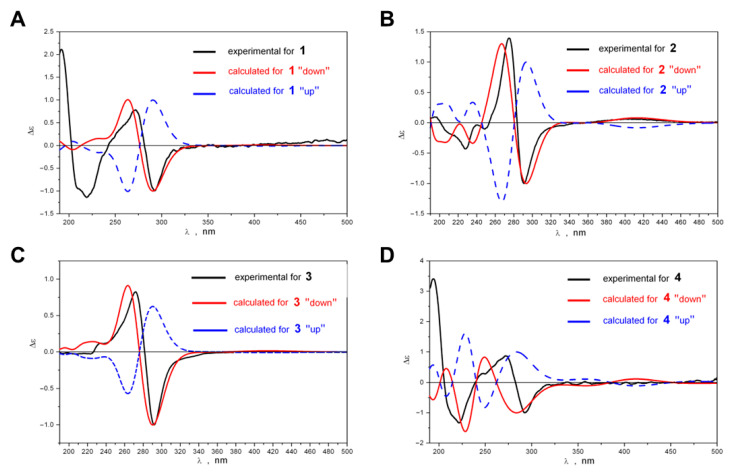
Experimental ECD spectra of compounds **1** (**A**), **2** (**B**), **3** (**C**) and **4** (**D**) compared to spectra theoretically calculated for “UP” and “DOWN” rotameric forms.

A comparison of the experimental ECD spectra of compounds **1**–**4** with the spectra theoretically calculated for the “UP” and “DOWN” rotameric forms is demonstrated in Figure 4A–D.

At the qualitative level, it is very useful to analyze ECD spectra scaled according to the intensity of the chosen band. In this work, we took the band at λ ≈ 290 nm as the reference band. As can be seen from Figure 4A–D, a good agreement between experimental and theoretical spectra occurred for “DOWN” rotameric forms—their ECD spectra correctly reproduced the positions, the signs and the relative intensities of two most characteristic bands and, hence, the sign of the Cotton effect in the region 240 ≤ λ ≤ 340 nm. For “UP’ conformations, such good correspondence did not occur. Our calculations showed that the rotation of hydroxyl groups at C-8 and C-8′ (LAM2) as well as rotation of propyl or methyl groups at C-2 and C-2′ (LAM3, 4) had only a slight influence on the positions and intensities of these two characteristic bands in the ECD spectra of compounds **1**–**4**, and did not change their signs. Thus, the shapes of ECD spectra were stable (at the qualitative level of comparison) during LAM2–LAM4 motions, and the signs of the characteristic bands in the experimental spectra were determined by the difference between the amounts of the “UP” and “DOWN” rotameric forms: Δg_up;down_ = (g_up,∑ −_ g_down,∑_). The good agreement between Δε_exp_ and Δε_down_ indicated that the “DOWN” rotameric form prevailed for compounds **1**–**4**.

### 3.3. ABTS Scavenging Activity of Compounds ***1***–***4***

Isolated bibenzochromenones **1**–**4** were tested for their ability to scavenge the ABTS cation radical. Compounds **1**, **3** and **4**, which have the connection of two fragments in the positions 7 and 9′, showed significant antiradical properties, higher than those of the positive control trolox (Table 2). Derivative **4** was the most active among the tested compounds; its activity was 2.2 times higher than that of trolox (Table 2). Compound **2** with fragments linked at positions 7 and 10′ appeared to be less active, perhaps due to steric constraints.

9,9′-Oxybis-neocomantherin—dimeric benzochromenone of *Comantheria rotula* along with monomeric benzochromenones were found to have only 40% of the DPPH radical scavenging capacity of trolox [14]. Compared to the DPPH assay, the ABTS assay better estimates the antioxidant capacity of phenolic compounds [40,41]. Also, the lesser activity of *C. rotula* compounds can be explained by the fact that all of them had methylated hydroxyl groups, which reduce antioxidant capacity.

### 3.4. Neuroprotective Properties of Compounds ***1***–***4***

The neuroprotective potential of compounds **1**–**4** was studied on Neuro-2a neuroblastoma cells exposed to the pesticide and complex I inhibitor rotenone, which reproduces features of Parkinson’s disease [42].

**Figure 5:**
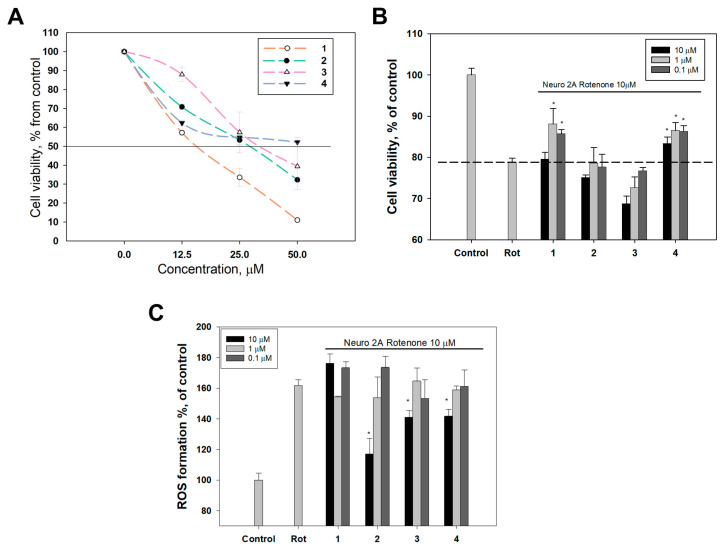
Neuroprotective properties of compounds **1**–**4**. (**A**) Cytotoxic effect of the compounds **1**–**4** on Neuro-2a neuroblastoma cells. Neuro-2a cells were incubated with compounds for 24 h. All experiments were carried out in triplicate. The data are presented as mean ± SEM. (**B**) The influence of compounds **1**–**4** on rotenone-treated Neuro-2a cells’ viability. Each bar represents the mean ± SEM of three independent replicates. (*) *p* < 0.05 vs. rotenone-treated cells. The difference between control and rotenone-treated cells was considered significant (*p* < 0.05). (**C**) The effect of compounds **1**–**4** on ROS levels in Neuro-2a cells treated with rotenone (10 µM). Each bar represents the mean ± SEM of three independent replicates. (*) *p* < 0.05 vs. rotenone-treated cells. The difference between control and rotenone-treated cells was considered significant (*p* < 0.05).

First, a cytotoxicity assay (MTT method) was carried out to determine the concentration range of compounds **1**–**4** for the subsequent study of their neuroprotective properties in the non-toxic range for the Neuro-2a cells. Derivative **4** showed the lowest toxicity (EC_50_ > 50 µM), and compounds **1**, **2** and **3** exhibited moderate toxicity with EC_50_ values of 15.3, 28.9 and 32.6 µM, respectively (Figure 5A). Further neuroprotective activity assays were performed at the concentrations of 10 µM and below for compounds **1**–**4**.

Next, the viability of Neuro-2a cells treated with the neurotoxin rotenone in the presence of compounds **1**–**4** was measured using the MTT test. Rotenone decreased the viability of Neuro-2a cells by 20% (Figure 5B).

Compounds **2** and **3** showed no neuroprotective effect, and compound **3** even slightly decreased the viability of rotenone-treated Neuro-2a cells in a dose-dependent manner. Derivative **4** statistically significantly increased the viability of rotenone-treated Neuro-2a cells at all concentrations tested (0.1–10 µM). The maximum effect was observed at a concentration of 1 µM and was 9.8% (Figure 5B). Compound **1** did not affect cell viability at a concentration of 10 µM, but significantly increased it at concentrations of 0.1 and 1 µM by 8.7% and 11.8%, respectively, so compound **1** can be considered the most potent neuroprotector among the studied compounds.

The production of reactive oxygen species (ROS) and the depletion of antioxidants have been identified as significant contributors to rotenone toxicity in rats [43]. The effect of compounds **1**–**4** on ROS levels in Neuro-2a cells treated with rotenone was studied. Incubation of Neuro-2a cells with rotenone (10 µM) caused an increase in ROS level by 59.38% compared to untreated cells (Figure 5C). None of the studied compounds statistically exhibited an ROS scavenging effect at concentrations of 0.1 and 1 µM, and compound **1** did not statistically exhibit an ROS scavenging effect at a concentration of 10 µM (Figure 5C). Compounds **2**, **3** and **4** at a concentration of 10 µM decreased the ROS formation in rotenone-treated cells by 37.8, 19.6, 17.6%, respectively (Figure 5C).

Interestingly, compound **2** had the lowest TEAC value according to the results of the ABTS assay provided above (Table 2) and it did not affect the viability of Neuro-2a cells exposed to rotenone (Figure 5B). On the contrary, compounds **1** and **4** exhibited high antiradical activity in the ABTS assay and increased the viability of Neuro-2a rotenone-treated cells, but did not prevent ROS formation in these cells (Figure 5B). It is known that the antioxidant efficacies of compounds may differ between in vitro and in vivo results [44], and it is known that there might be multiple sources of oxidative damage in Parkinson’s disease models [42], so further investigations of complex antioxidant and neuroprotective activities of crinoid benzochromenones are required.

### 3.5. Antimicrobial Activity of Compounds ***1***–***4***

The search for new antimicrobial agents remains an urgent task due to the increasing identification of antibiotic-resistant pathogens. *Candida albicans* and *Staphylococcus* species are, respectively, the most common fungal and bacterial agents isolated from bloodstream infections worldwide [45]. They are well-known for their ability to form persistent biofilms in host tissues and indwelling medical devices [46]. So, in this study, we investigated the antimicrobial properties of compounds **1**–**4** towards *C. albicans*, *S. aureus* and their biofilms.

**Figure 6:**
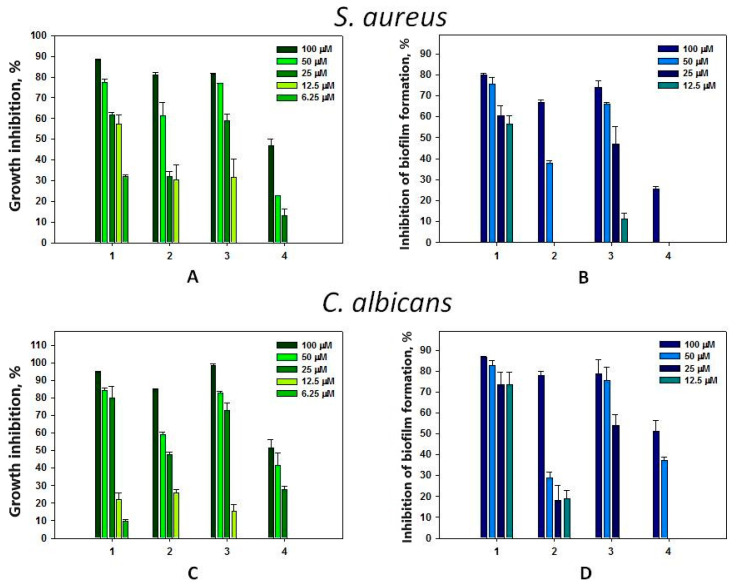
Antimicrobial activity of compounds **1**–**4** against *S. aureus* (**A**); against *S. aureus* biofilm formation (**B**); against *C. albicans* (**C**); and against *C. albicans* biofilm formation (**D**). Each bar represents the mean ± SEM of six independent replicates.

Compound **1** at concentrations from 6.25 to 100 μM dose-dependently inhibited the growth of Gram-positive bacteria *S. aureus* and yeast-like fungi *C. albicans* up to 90% (Figure 6A,C). However, this compound more effectively inhibited the biofilm formation of both microorganisms starting from a concentration of 12.5 μM (Figure 6B,D). The antimicrobial activity of compound **3** against *S. aureus* and *C. albicans* in general was comparable to that of compound **1** (Figure 6B,D). Inhibition of *S. aureus* biofilm formation was comparable to its ability to inhibit the growth of planktonic forms (Figure 6B); however, compound **3** significantly prevented *C. albicans* biofilm formation at only 50–100 μM concentrations (Figure 6D). Compound **2** exhibited less pronounced antimicrobial activity against *C. albicans* and *S. aureus*. Significant inhibition of biofilm formation by *C. albicans* and *S. aureus* was shown for compound **2** only at a concentration of 100 μM. Compound **4** had the least inhibitory activity on *S. aureus* growth and biofilm formation compared to the other tested compounds. There was a negligible effect only at its concentration of 100 μM (Figure 6B).

The studied compounds did not show antimicrobial activity against Gram-negative bacteria *E. coli* and did not inhibit the formation of biofilms by them at concentrations up to 100 μM. Crinemodin–rhodoptilometrin bianthrone isolated from *Himerometra magnipinna* slightly inhibited hyphae formation of a *Streptomyces* strain [15]. Gymnochromes E and F inhibited *Staphylococcus aureus* and methicillin-resistant *S. aureus* (MRSA) with minimum inhibition concentrations (MICs) of 25 and 12.5 μg/mL, respectively, and did not show activity against either *Pseudomonas aeruginosa* or *C. albicans* at the concentrations tested [28]. Converted to micromoles (~30 μM), these results are comparable to those obtained by us, so crinoids can be considered a source of antimicrobial agents.

Cytochrome P450 and steroid dehydrogenases are pharmaceutically significant drug targets. However, for most of these targets, there is no effective, selective and safe regulator. In this regard, many laboratories and pharmaceutical companies around the world are working on producing selective modulators of cytochrome P450 and human steroid dehydrogenase. The interaction of compound **1** with eight recombinant cytochrome P450 (*Mycobacterium tuberculosis* (CYP51, CYP124, CYP125, CYP126, CYP142), *Candida albicans* (CYP51), *Candida glabrata* (CYP51), *Homo sapiens* (CYP51) was analyzed using surface plasmon resonance technology and the rate constants of association (kon, ka) and dissociation (koff, kd), and the equilibrium dissociation constants (KD) of the complexes were determined (Table S5). Compound **1** demonstrated a Kd value of more than 10 μM, indicating a possible nonspecific interaction, which excludes compound **1** from potential selective modulators of cytochrome P450.

## 4. Conclusions

In conclusion, three new dimeric benzochromenones named phanogracilins A–C (**1**–**3**) were isolated from the crinoid *Phanogenia gracilis*, and the structure of **1** was established using X-ray crystallography, which allowed us to reliably assign 2D NMR signals for compound **1** and subsequently for its isomer **2**, and for compound **3**. As far as we know, this is the first example of C–C-connected benzochromenone molecules with demethylated hydroxy groups, which are not typical for crinoids. After purification of **1** using Na_2_CO_3_, compound **4** was derived from **1** in alkaline conditions, and its structure was elucidated as 5,5′,6′,8,8′-pentahydroxy-2,2′-dipropyl-4H,4′H-[7,9′-bibenzo[g]chromene]-4,4′,6,9-tetraone. Compounds **1**–**4** were found to be optically active despite the lack of stereo centers, and conformational analysis using density functional theory (DFT) was performed.

Isolated bibenzochromenones **1**–**4** were tested for their ability to scavenge the ABTS cation radical. Compounds **1**, **3** and **4** showed significant antiradical properties, higher than positive control trolox. Despite that, compounds **1** and **4** did not prevent ROS formation in rotenone-treated Neuro-2a cells in the model of Parkinson’s disease, at the same time increasing their viability. Compound **2** had the lowest radical scavenging activity according to the results of the ABTS assay and also did not affect the viability of Neuro-2a cells exposed to rotenone, but it significantly decreased ROS formation in rotenone-treated cells. We suggest that further investigations of the antioxidant and neuroprotective activities of compounds **1**–**4** are required.

Compounds **1** and **3** at concentrations from 25 to 100 μM dose-dependently inhibited the growth of Gram-positive bacteria *S. aureus* and yeast-like fungi *C. albicans*, and they also prevented the formation of biofilms of these microorganisms, so they can be considered potent antimicrobials.

In sum, crinoids remain a promising source of secondary metabolites with novel chemical structures, mainly of quinonoid nature, and with biomedical potential.

## Figures and Tables

**Table 1:** NMR data for compounds **1**–**4** (DMSO-*d*_6_, 500 MHz for ^1^H and 126 MHz for ^13^C, *δ*, ppm, *J*/Hz).

Position	1		2		3		4	
	*δ*_C_	*δ*_H_	*δ*_C_	*δ*_H_	*δ*_C_	*δ*_H_	*δ*_C_	*δ*_H_
2	173.42		174.51		174.42		171.96	
3	106.23 ^a^	6.20 (1H, s, H)	106.57 ^c^	6.27 (1H, s, H)	106.58 ^f^	6.26 (1H, s, H)	112.12	6.37 (1H, s, H)
4	184.21		184.69		184.67		180.83	
5	152.65		153.38		153.17		160.73	14.18 (1H, s, OH)
6	157.09	9.77 (1H, s, OH)	157.51	9.85 (1H, s, OH)	157.57	9.73 (1H, s, OH)	186.52	
7	109.13		107.85		109.60		162.32 ^g^	
8	161.44	10.03 (1H, s, OH)	161.54	10.13 (1H, s, OH)	161.93	9.96 (1H, s, OH)	159.72 ^g^	9.90 ^h^ (1H, s, OH)
9	101.72	6.84 (1H, s, H)	102.21	6.85 (1H, s, H)	102.20	6.80 (1H, s, H)	181.44	
10	100.58	7.18 (1H, s, H)	101.12	7.18 (1H, s, H)	101.04	7.14 (1H, s, H)	107.84	7.61 (1H, s, H)
11	163.45		164.25		163.88		163.58	
12	102.52		103.19 ^d^		103.07		116.41	
13	106.11 ^a^		106.49 ^c^		106.44 ^f^		113.44	
14	140.23		141.27		140.72		136.92	
15	36.28	2.56 (2H, t, *J* = 7.2, CH_2_)	36.92	2.68 (2H, t, *J* = 7.2, CH_2_)	36.92	2.68 (2H, t, *J* = 7.2, CH_2_)	36.19	2.68 (2H, t, *J* = 7.2, CH_2_)
16	20.51	1.64 (2H, m, *J* = 7.2, CH_2_)	21.05	1.75 (2H, m, *J* = 7.2, CH_2_)	21.01	1.75 (2H, m, *J* = 7.2, CH_2_)	20.79	1.75 (2H, m, *J* = 7.2, CH_2_)
17	14.16	0.91 (3H, t, *J* = 7.2, CH_3_)	14.68	0.99 (3H, t, *J* = 7.2, CH_3_)	14.69	0.99 (3H, t, *J* = 7.2, CH_3_)	14.63	0.98 (3H, t, *J* = 7.2, CH_3_)
2′	173.93		173.96		171.15		173.95	
3′	105.98 ^a^	6.31 (1H, s, H)	106.57 ^c^	6.21 (1H, s, H)	107.03 ^f^	6.17 (1H, s, H)	106.80	6.19 (1H, s, H)
4′	184.26		185.05		184.69		184.79	
5′	152.70		150.84		153.08		153.49	
6′	158.63	9.87 ^b^ (1H, s, OH)	160.33	9.98 ^e^ (1H, s, OH)	159.08	9.87 (1H, s, OH)	162.32	
7′	101.77	6.59 (1H, s, H)	101.85	6.30 (1H, d, *J* = 2.2, H)	102.23	6.54 (1H, s, H)	101.90	6.48 (1H, s, H)
8′	159.71	9.93 ^b^ (1H, s, OH)	162.17	9.98 ^e^ (1H, s, OH)	160.17	9.81 (1H, s, OH)	159.87	9.99 ^h^ (1H, s, OH)
9′	106.54		100.67	6.19 (1H, d, *J* = 2.2, H)	107.13		104.63	
10′	99.09	6.43 (1H, s, H)	105.88		99.57	6.36 (1H, s, H)	100.39	6.88 (1H, s, H)
11′	163.53		163.66		164.02		163.89	
12′	102.59		103.25 ^d^		102.83		103.22	
13′	106.23 ^a^		106.87 ^c^		106.71 ^f^		106.93	
14′	140.30		140.95		140.72		140.19	
15′	36.44	2.72 (2H, t, *J* = 7.2, CH_2_)	36.66	2.43 (2H, t, *J* = 7.2, CH_2_)	21.57	2.27 (3H, t, *J* = 7.2, CH_3_)	36.85	2.56 (2H, t, *J* = 7.2, CH_2_)
16′	20.56	1.79 (2H, m, *J* = 7.2, CH_2_)	20.54	1.45 (2H, m, *J* = 7.2, CH_2_)	-		20.99	1.65 (2H, m, *J* = 7.2, CH_2_)
17′	14.24	1.03 (3H, t, *J* = 7.2, CH_3_)	14.37	0.77 (3H, t, *J* = 7.2, CH_3_)	-		14.65	0.89 (3H, t, *J* = 7.2, CH_3_)

^a–h^ These values can be interchanged.

**Table 2:** ABTS scavenging activity of compounds **1**–**4**.

Compound	IC_50_, mkg/mL	IC_50_, µM	TEAC
**1**	5.96 ± 0.31	10.46	1.2
**2**	17.26 ± 0.32	30.28	0.4
**3**	5.11 ± 0.09	9.43	1.3
**4**	3.35 ± 0.05	5.73	2.2
Trolox	3.32 ± 0.05	12.41	1

## Data Availability

Data are contained within the article and Supplementary Materials.

## Supplementary Materials

The following supporting information can be downloaded at: https://www.mdpi.com/article/10.3390/biom14020151/s1, Figure S1: HPLC profile of ethanolic extract of *P. gracilis*, when no acid was added; Figure S2: HPLC profile of acidified ethanolic extract of *P. gracilis*; Figure S3: Data comparison of HPLC profiles of an ethanolic and acidified ethanolic extract of *P. gracilis*; Figure S4: HPLC profile of the CHCl3 fraction of the acidified ethanolic extract of *P. gracilis*; Figure S5: HPLC profile of the EtOAc fraction of the acidified ethanolic extract of *P. gracilis*; Figure S6: Absorption spectrum of **3** (DAD); Figure S7: MS data of **3**; Figure S8: Absorption spectrum of **2** (DAD); Figure S9: MS data of **2**; Figure S10: Absorption spectrum of **1** (DAD); Figure S11: MS data of **1**; Figure S12: HRESIMS data of **1** and **2**; Figure S13: HRESIMS data of **3**; Figure S14: IR spectrum of **1** (1900–1000 cm^−1^); Figure S15: IR spectrum of **1** (3800–2000 cm^−1^); Figure S16: ^1^H NMR spectrum of **1** (DMSO-*d*_6_, 500 MHz); Figure S17: Enlarged fragment of ^1^H NMR spectrum of **1** (DMSO-*d*_6_, 500 MHz); Figure S18: Enlarged fragment of ^1^H NMR spectrum of **1** (DMSO-*d*_6_, 500 MHz); Figure S19: Enlarged fragment of ^1^H NMR spectrum of **1** (DMSO-*d*_6_, 500 MHz); Figure S20: ^1^H NMR spectrum of **1** (acetone-*d*_6_, 700 MHz); Figure S21: ^13^C NMR spectrum of **1** (DMSO-*d*_6_, 126 MHz); Figure S22: Enlarged fragment of ^13^C NMR spectrum of **1** (DMSO-*d*_6_, 126 MHz); Figure S23: Enlarged fragment of ^13^C NMR spectrum of **1** (DMSO-*d*_6_, 126 MHz); Figure S24: Enlarged fragment of ^13^C NMR spectrum of **1** (acetone-*d*_6_, 126 MHz); Figure S25: Enlarged fragment of ^13^C NMR spectrum of **1** (DMSO-*d*_6_, 126 MHz); Figure S26: HMBC correlations for **1**; Table S1: Crystal data and structural refinement for 1; Table S2: Selected bond lengths in the structure of **1**; Table S3: Hydrogen bonds in the structure of **1** (Å and °); Figure S27: ^1^H NMR spectrum of **2** (DMSO-*d*_6_, 500 MHz); Figure S28: Enlarged fragment of ^1^H NMR spectrum of **2** (DMSO-*d*_6_, 500 MHz); Figure S29: Enlarged fragment of ^1^H NMR spectrum of **2** (DMSO-*d*_6_, 500 MHz); Figure S30: Enlarged fragment of ^1^H NMR spectrum of **2** (DMSO-*d*_6_, 500 MHz); Figure S31: ^13^C NMR spectrum of **2** (DMSO-*d*_6_, 126 MHz); Figure S32: Enlarged fragment of ^13^C NMR spectrum of **2** (DMSO-*d*_6_, 126 MHz); Figure S33: Enlarged fragment of ^13^C NMR spectrum of **2** (DMSO-*d*_6_, 126 MHz); Figure S34: HMBC spectrum of **2**; Figure S35: ^1^H NMR spectrum of **3** (DMSO-*d*_6_, 500 MHz); Figure S36: Enlarged fragment of ^1^H NMR spectrum of **3** (DMSO-*d*_6_, 500 MHz); Figure S37: Enlarged fragment of ^1^H NMR spectrum of **3** (DMSO-*d*_6_, 500 MHz); Figure S38: Enlarged fragment of ^1^H NMR spectrum of **3** (DMSO-*d*_6_, 500 MHz); Figure S39: ^13^C NMR spectrum of **3** (DMSO-*d*_6_, 126 MHz); Figure S40: Enlarged fragment of ^13^C NMR spectrum of **3** (DMSO-*d*_6_, 126 MHz); Figure S41: Enlarged fragment of ^13^C NMR spectrum of **3** (DMSO-*d*_6_, 126 MHz); Figure S42: HMBC spectrum of **3**; Figure S43: HPLC profile of compound **1** after purification using Na_2_CO_3_; Figure S44: Absorption spectrum of compound **4** (DAD); Figure S45: MS data of compound **4**; Figure S46: HRESIMS data of compound **4**; Figure S47: ^1^H NMR spectrum of **4** (DMSO-*d*_6_, 500 MHz); Figure S48: Enlarged fragment of ^1^H NMR spectrum of **4** (DMSO-*d*_6_, 500 MHz); Figure S49: Enlarged fragment of ^1^H NMR spectrum of **4** (DMSO-*d*_6_, 500 MHz); Figure S50: Enlarged fragment of ^1^H NMR spectrum of **4** (DMSO-*d*_6_, 500 MHz); Figure S51: ^13^C NMR spectrum of **4** (DMSO-*d*_6_, 126 MHz); Figure S52: Enlarged fragment of ^13^C NMR spectrum of **4** (DMSO-*d*_6_, 126 MHz); Figure S53: Enlarged fragment of ^13^C NMR spectrum of **4** (DMSO-*d*_6_, 126 MHz); Figure S54: HMBC spectrum of **4** (DMSO-*d*_6_, 126 MHz); Table S4: The amount of the ligand immobilized on the chip; Table S5: Parameters of interaction of compound **1** with cytochromes of various organisms immobilized on the CM5 optical chip.
