# Supplementary material for: Phanogracilins A–C, New Bibenzochromenones of Crinoid Phanogenia gracilis (Hartlaub, 1890)
Source: Biomolecules. 2024 Jan 26;14(2):151. doi: 10.3390/biom14020151 (PMC10887160; doi:10.3390/biom14020151)
Supplement: Supplementary file 1 [file biomolecules-14-00151-s001.zip › biomolecules-2821532-supplementary.pdf]

# - Supporting Information –

Phanogracilins A-C, new bibenzochromenones of crinoid *Phanogenia gracilis* (Hartlaub, 1890)

Vasileva E.A.[1], Berdyshev D.V.[1], Mishchenko N.P.[1], Gerasimenko A.V.[2], Menchinskaya E.S.[1], Pislyagin E.A.[1], Chingizova E.A.[1], Kaluzhskiy L.A.[3], Dautov S.Sh.[4], Fedoreyev S.A.[1]

[1] G.B. Elyakov Pacific Institute of Bioorganic Chemistry, Far Eastern Branch of the Russian Academy of Sciences, Vladivostok 690022, Russia;

[2] Institute of Chemistry, Far Eastern Branch of the Russian Academy of Sciences, Vladivostok 690022, Russia;

[3] Institute of Biomedical Chemistry, Moscow 119121, Russia;

[4] A.V. Zhirmunsky National Scientific Center of Marine Biology, Far Eastern Branch of the Russian Academy of Sciences, Vladivostok 690041, Russia;

* Correspondence: vasilieva_el_an@mail.ru; Tel.: +79025272055

Table of contents

Figure S1. HPLC profile of ethanolic extract of *P. gracilis*, no acid was added 4

Figure S2. HPLC profile of acidified ethanolic extract of *P. gracilis* 4

Figure S3. Data comparison of HPLC profiles of an ethanolic and of acidified ethanolic extract of *P. gracilis* 5

Figure S4. HPLC profile of the $CHCl_3$ fraction of the acidified ethanolic extract of *P. gracilis* 5

Figure S5. HPLC profile of the EtOAc fraction of the acidified ethanolic extract of *P. gracilis* 5

Figure S6. Absorption spectrum of **3** (DAD) 6

Figure S7. MS data of **3** 6

Figure S8. Absorption spectrum of **2** (DAD) 6

Figure S9. MS data of **2** 7

Figure S10. Absorption spectrum of **1** (DAD) 7

Figure S11. MS data of **1** 7

Figure S12. HRESIMS data of **1** and **2** 8

Figure S13. HRESIMS data of **3** 9

Figure S14. IR spectrum of **1** (1900-1000 cm^{-1}) 10

Figure S15. IR spectrum of **1** (3800-2000 cm^{-1}) 11

Figure S16. ^{1}H NMR spectrum of **1** (DMSO-d_6, 500 MHz) 12

Figure S17. Enlarged fragment of ^{1}H NMR spectrum of **1** (DMSO-d_6, 500 MHz) 13

Figure S18. Enlarged fragment of ^{1}H NMR spectrum of **1** (DMSO-d_6, 500 MHz) 14

Figure S19. Enlarged fragment of ^{1}H NMR spectrum of **1** (DMSO-d_6, 500 MHz) 15

Figure S20. ^{1}H NMR spectrum of **1** (acetone-d_6, 700 MHz) 16

Figure S21. ^{13}C NMR spectrum of **1** (DMSO-d_6, 126 MHz) 17

Figure S22. Enlarged fragment of ^{13}C NMR spectrum of **1** (DMSO-d_6, 126 MHz) 18

Figure S23. Enlarged fragment of ^{13}C NMR spectrum of **1** (DMSO-d_6, 126 MHz) 19

Figure S24. Enlarged fragment of ^{13}C NMR spectrum of **1** (acetone-d_6, 126 MHz) 20

Figure S25. Enlarged fragment of ^{13}C NMR spectrum of **1** (DMSO-d_6, 126 MHz) 21

Figure S26. HMBC correlations for **1** 22

Table S1. Crystal data and structure refinement for **1** 23

Table S2. Selected bond lengths in the structure of **1** 24

Table S3. Hydrogen bonds in the structure of **1** (Å and °) 27

Figure S27. ^{1}H NMR spectrum of **2** (DMSO-d_6, 500 MHz) 28

Figure S28. Enlarged fragment of ^{1}H NMR spectrum of **2** (DMSO-d_6, 500 MHz) 29

Figure S29. Enlarged fragment of ^{1}H NMR spectrum of **2** (DMSO-d_6, 500 MHz)....30
Figure S30. Enlarged fragment of ^{1}H NMR spectrum of **2** (DMSO-d_6, 500 MHz)....31
Figure S31. ^{13}C NMR spectrum of **2** (DMSO-d_6, 126 MHz)....32
Figure S32. Enlarged fragment of ^{13}C NMR spectrum of **2** (DMSO-d_6, 126 MHz)....33
Figure S33. Enlarged fragment of ^{13}C NMR spectrum of **2** (DMSO-d_6, 126 MHz)....34
Figure S34. HMBC spectrum of **2**....35
Figure S35. ^{1}H NMR spectrum of **3** (DMSO-d_6, 500 MHz)....36
Figure S36. Enlarged fragment of ^{1}H NMR spectrum of **3** (DMSO-d_6, 500 MHz)....37
Figure S37. Enlarged fragment of ^{1}H NMR spectrum of **3** (DMSO-d_6, 500 MHz)....38
Figure S38. Enlarged fragment of ^{1}H NMR spectrum of **3** (DMSO-d_6, 500 MHz)....39
Figure S39. ^{13}C NMR spectrum of **3** (DMSO-d_6, 126 MHz)....40
Figure S40. Enlarged fragment of ^{13}C NMR spectrum of **3** (DMSO-d_6, 126 MHz)....41
Figure S41. Enlarged fragment of ^{13}C NMR spectrum of **3** (DMSO-d_6, 126 MHz)....42
Figure S42. HMBC spectrum of **3**....43
Figure S43. HPLC profile of compound **1** after purification using Na_2CO_3....44
Figure S44. Absorption spectrum of compound **4** (DAD)....44
Figure S45. MS data of compound **4**....44
Figure S46. HRESIMS data of compound **4**....45
Figure S47. ^{1}H NMR spectrum of **4** (DMSO-d_6, 500 MHz)....46
Figure S48. Enlarged fragment of ^{1}H NMR spectrum of **4** (DMSO-d_6, 500 MHz)....47
Figure S49. Enlarged fragment of ^{1}H NMR spectrum of **4** (DMSO-d_6, 500 MHz)....48
Figure S50. Enlarged fragment of ^{1}H NMR spectrum of **4** (DMSO-d_6, 500 MHz)....49
Figure S51. ^{13}C NMR spectrum of **4** (DMSO-d_6, 126 MHz)....50
Figure S52. Enlarged fragment of ^{13}C NMR spectrum of **4** (DMSO-d_6, 126 MHz)....51
Figure S53. Enlarged fragment of ^{13}C NMR spectrum of **4** (DMSO-d_6, 126 MHz)....52
Figure S54. HMBC spectrum of **4** (DMSO-d_6, 126 MHz)....53
Investigation of interaction of compound **1** with recombinant cytochromes P450 using SPR analysis....54
Table S4. The amount of the ligand immobilized on the chip....54
Table S5. Parameters of interaction of compound **1** with cytochromes of various organisms immobilized on the CM5 optical chip.....55

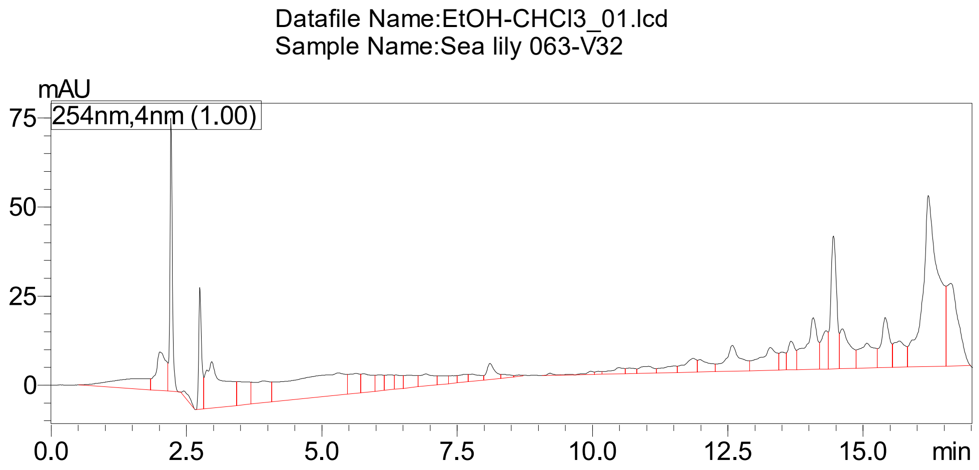

Figure S1. HPLC profile of ethanolic extract of *P. gracilis*, no acid was added. HPLC system of H_2O (A): MeCN (B) with the addition of 0.2% AcOH first was used not as described in manuscript, but as follows: 0–6 min, 10–40% (B); 6–11 min, 40–100% (B); 11–12 min, 100% (B), 12–13 min, 100–10% (B); and 13–17 min, 10% (B).

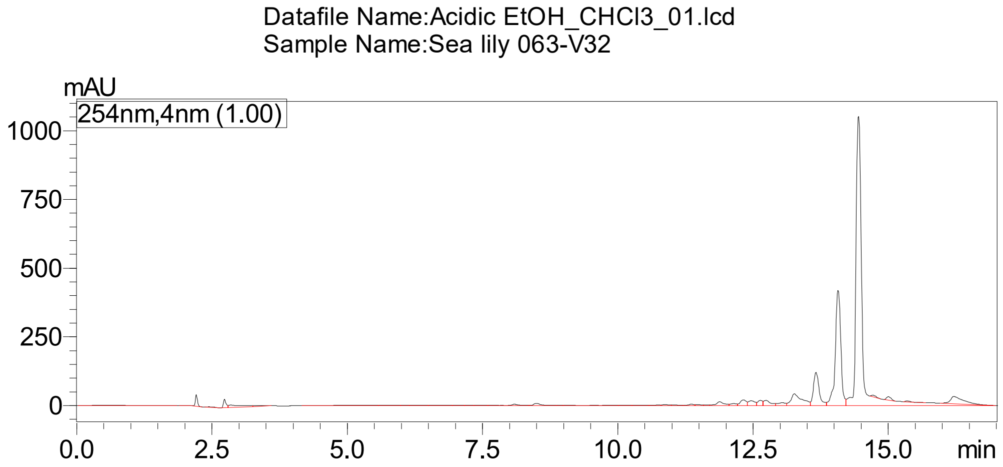

Figure S2. HPLC profile of acidified ethanolic extract of P. gracilis. HPLC system of H2O (A): MeCN (B) with the addition of 0.2% AcOH: 0–6 min, 10–40% (B); 6–11 min, 40–100% (B); 11–12 min, 100% (B), 12–13 min, 100–10% (B); and 13–17 min, 10% (B).

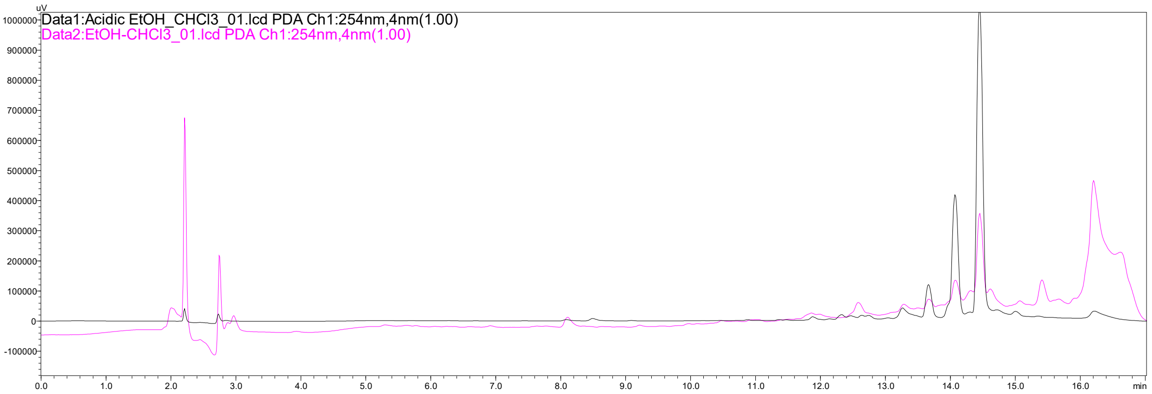

Figure S3. Data comparison of HPLC profiles of an ethanolic and of acidified ethanolic extract of P. gracilis. Coincidence of compounds **1-3** in both extracts was confirmed by coincidence of their HPLC-DAD-MS parameters.

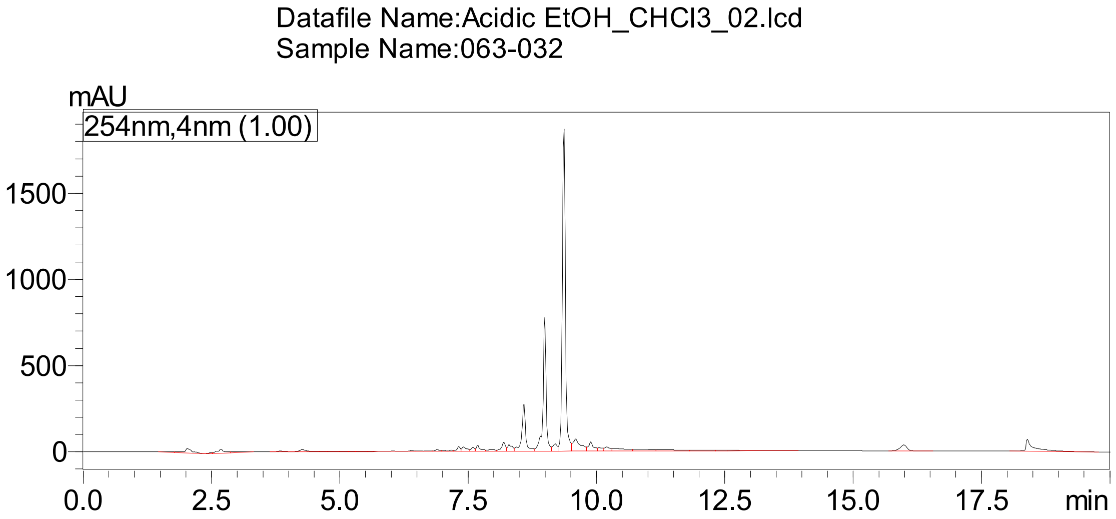

Figure S4. HPLC profile of the $CHCl_3$ fraction of the acidified ethanolic extract of *P. gracilis*

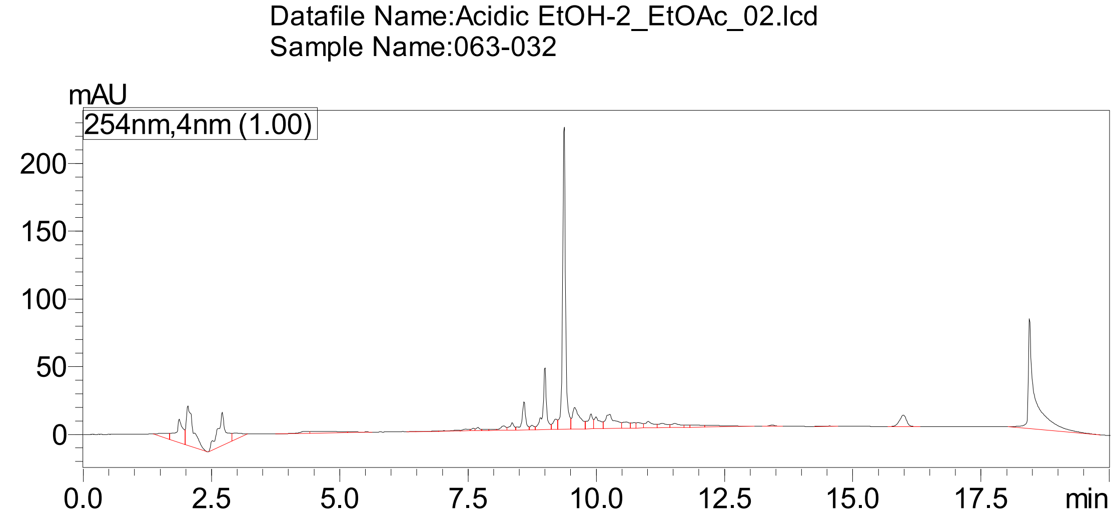

Figure S5. HPLC profile of the EtOAc fraction of the acidified ethanolic extract of *P. gracilis*

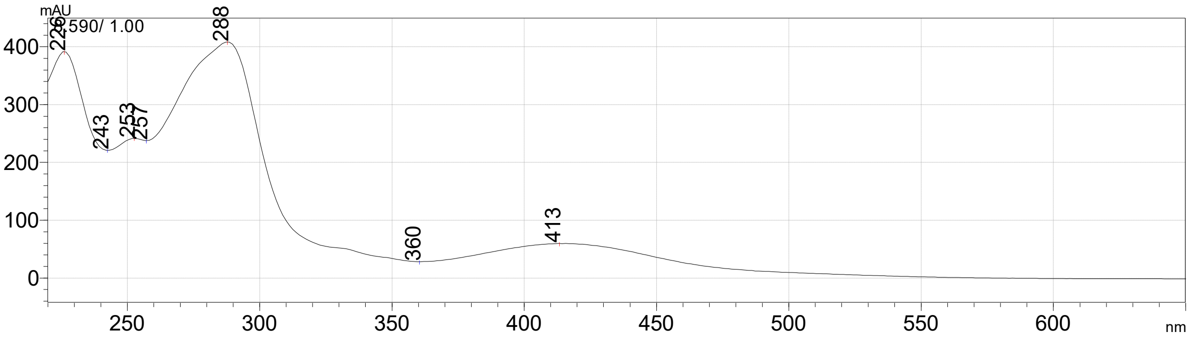

Figure S6. Absorption spectrum of **3** (DAD)

Scan(E-) Ret. Time : [8.667] Scan# : [1041]

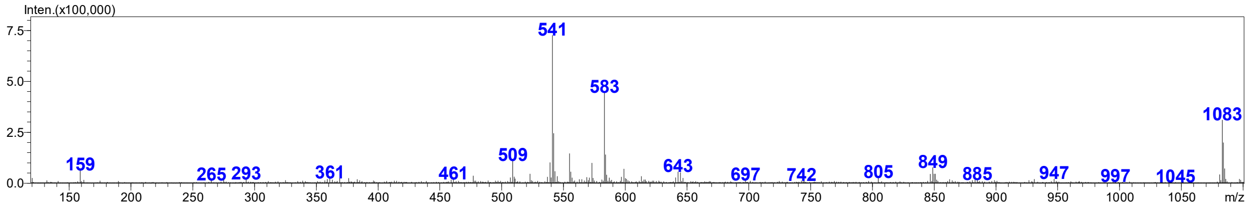

Scan(E+) Ret. Time : [8.675] Scan# : [1042]

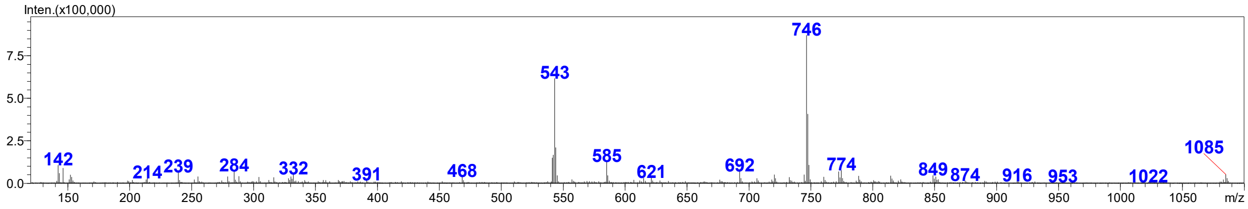

Figure S7. MS data of **3**

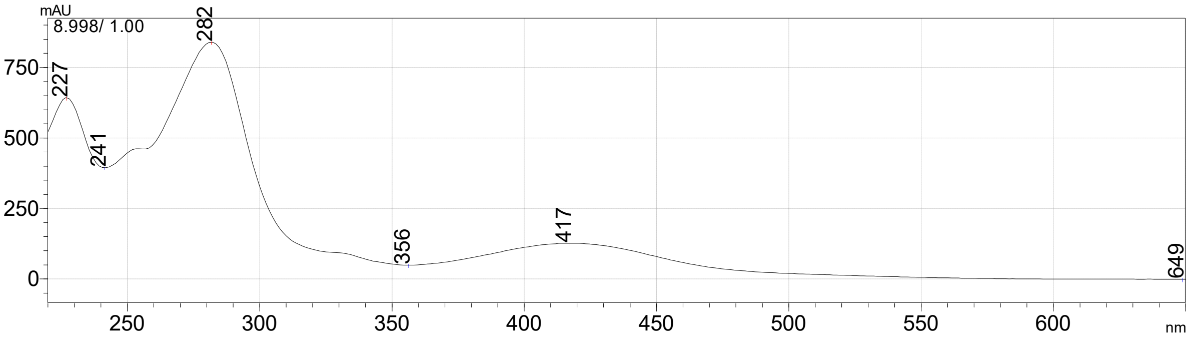

Figure S8. Absorption spectrum of **2** (DAD)

Scan(E-) Ret. Time : [9.033] Scan# : [1085]

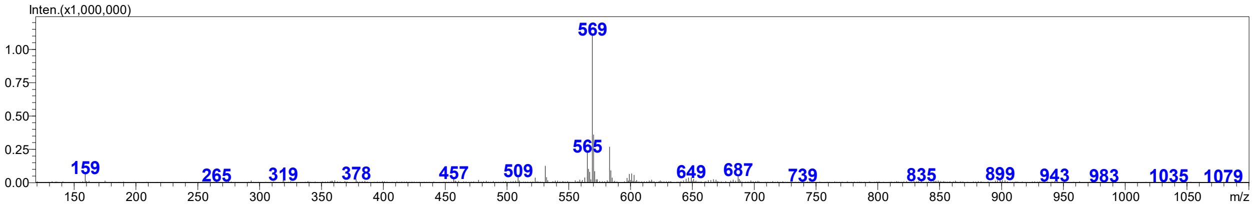

Scan(E+) Ret. Time : [9.041] Scan# : [1086]

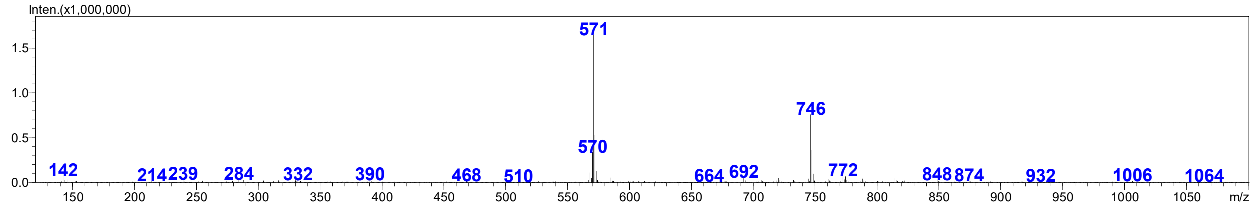

Figure S9. MS data of **2**

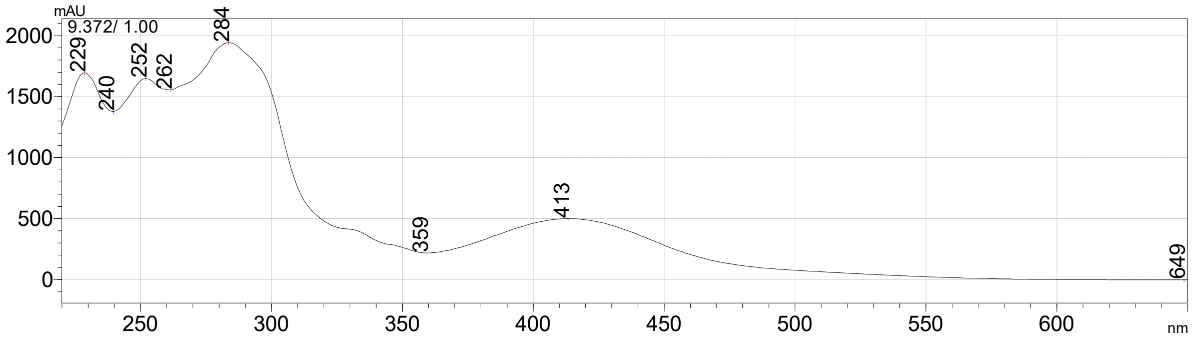

Figure S10. Absorption spectrum of **1** (DAD)

Scan(E-) Ret. Time : [9.417] Scan# : [1131]

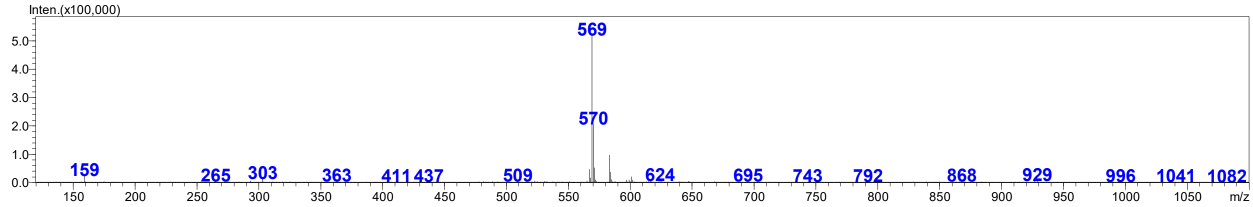

Scan(E+) Ret. Time : [9.425] Scan# : [1132]

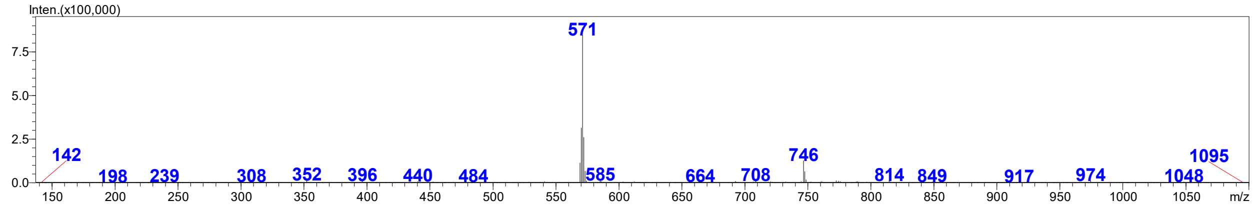

Figure S11. MS data of **1**

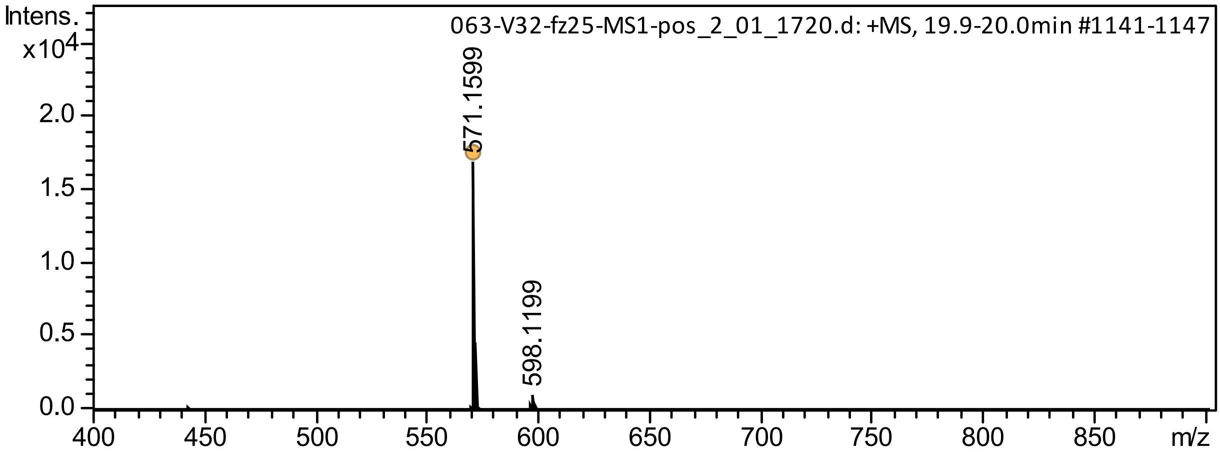

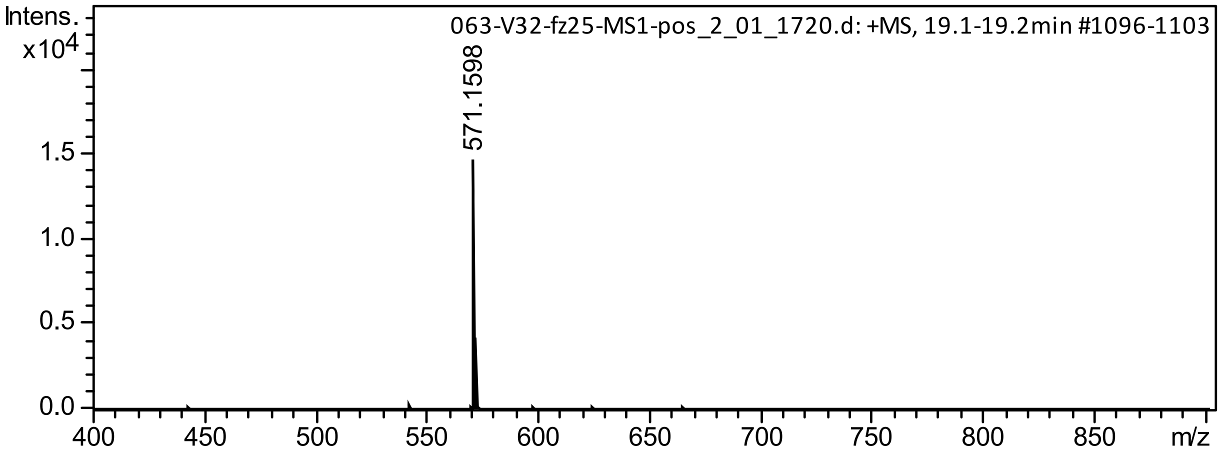

Meas. m/z	Ion Formula	m/z	err [ppm]	mSigma	Score	rdb
571,1598	C32H27O10	571,1599	0,1	34,2	100	20

Figure S12. HRESIMS data of **1** and **2**

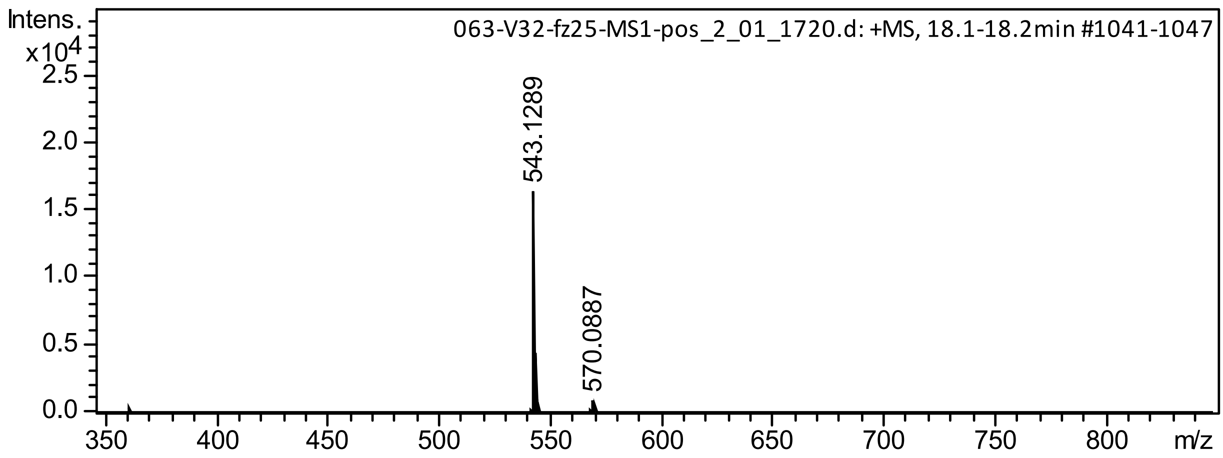

Meas. m/z	Ion Formula	m/z	err [ppm]	mSigma	Score	rdb
543,1289	C18H27N2O17	543,1304	2,9	26,7	61,16	7
	**C30H23O10**	**543,1286**	**-0,5**	**35,5**	**100**	**20**
	C31H19N4O6	543,1299	1,9	46,6	49,92	25

Figure S13. HRESIMS data of **3**

HR-ESI-MS Analysis

Chromatographic separation was carried out on a Bruker Elute UHPLC chromatograph (Bruker Daltonics, Bremen, Germany) in gradient elution mode. The separation was carried out on a Agilent InfinityLab Poroshell 120 SB-C18 column (2,1 × 150 mm, 1.9 µm particle size, Agilent Technologies, USA). The column was thermostated at 40 °C. The mobile phase used was a 0.1% aqueous solution of formic acid (A) and a 0.1% solution of formic acid in acetonitrile (B) at a flow rate of 0.35 mL/min. The gradient was as follows: 0 min, 3% (B); 0–3 min, 5% (B); 3–18 min, 70% (B); 18–21 min, 100% (B); 21–25 min, 100% (B); 25–28 min, 3% (B). The injection volume was 3 µL in the mode of partial filling of the autosampler loop.

Mass spectrometric detection was carried out on a Bruker Maxis Impact II instrument (Bruker Daltonics, Bremen, Germany) with an operating resolution of 20000, equipped with an electrospray ionization source in positive ion detection mode in the range of 50–2000 Da. The operating parameters of the ionization source were as follows: capillary voltage – 4500 V, nitrogen was used as a drying gas at a flow of 6 L/min, a pressure of 2.5 bar and a temperature of 200 °C. The voltage on the first and second ion funnel was 300 V, the voltage on the quadrupole and collision cell lenses was 5 and 10 V. MS/MS spectra in the collision-induced dissociation mode were obtained automatically, using nitrogen as the collisional activation gas. To obtain MS/MS spectra, peaks in the range of 500-600 m/z with an intensity greater than 800 p.u. were selected. The mass spectrometer was calibrated using a standard calibration solution (ESI-L Low Concentration Tuning Mix, Agilent Technologies, USA).

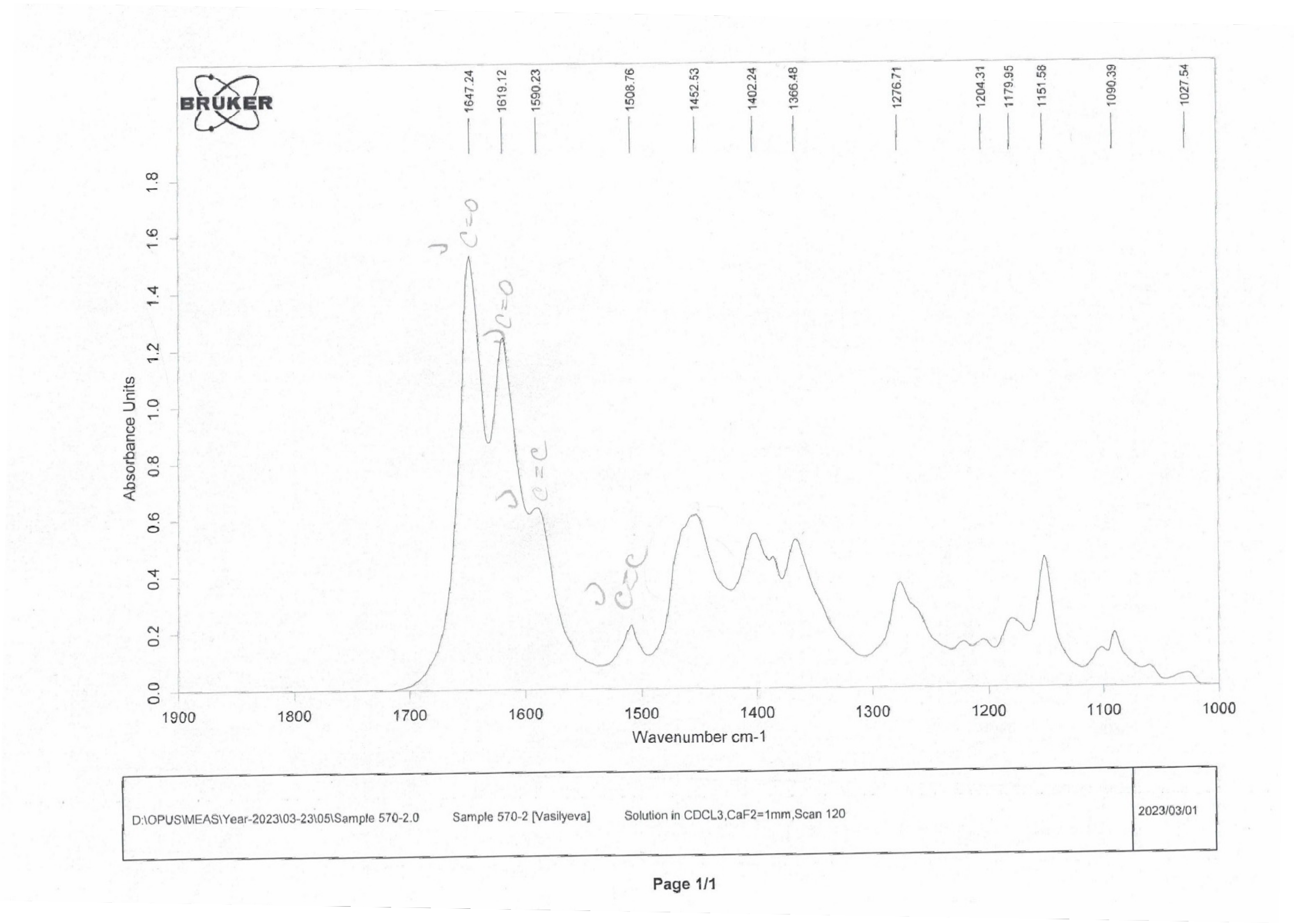

Figure S14. IR spectrum of **1** (1900-1000 cm^{-1})

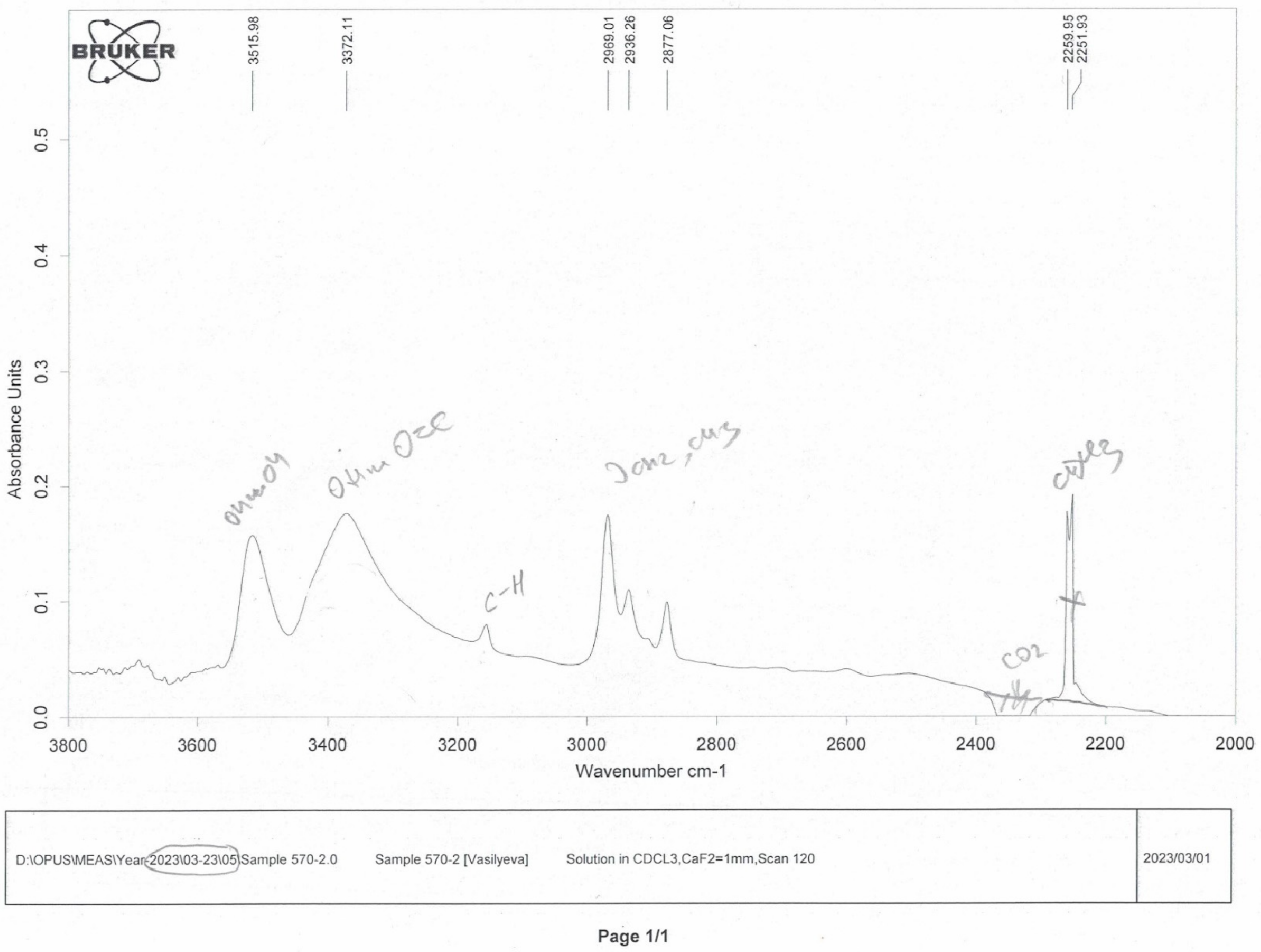

Figure S15. IR spectrum of **1** (3800-2000 cm-1)

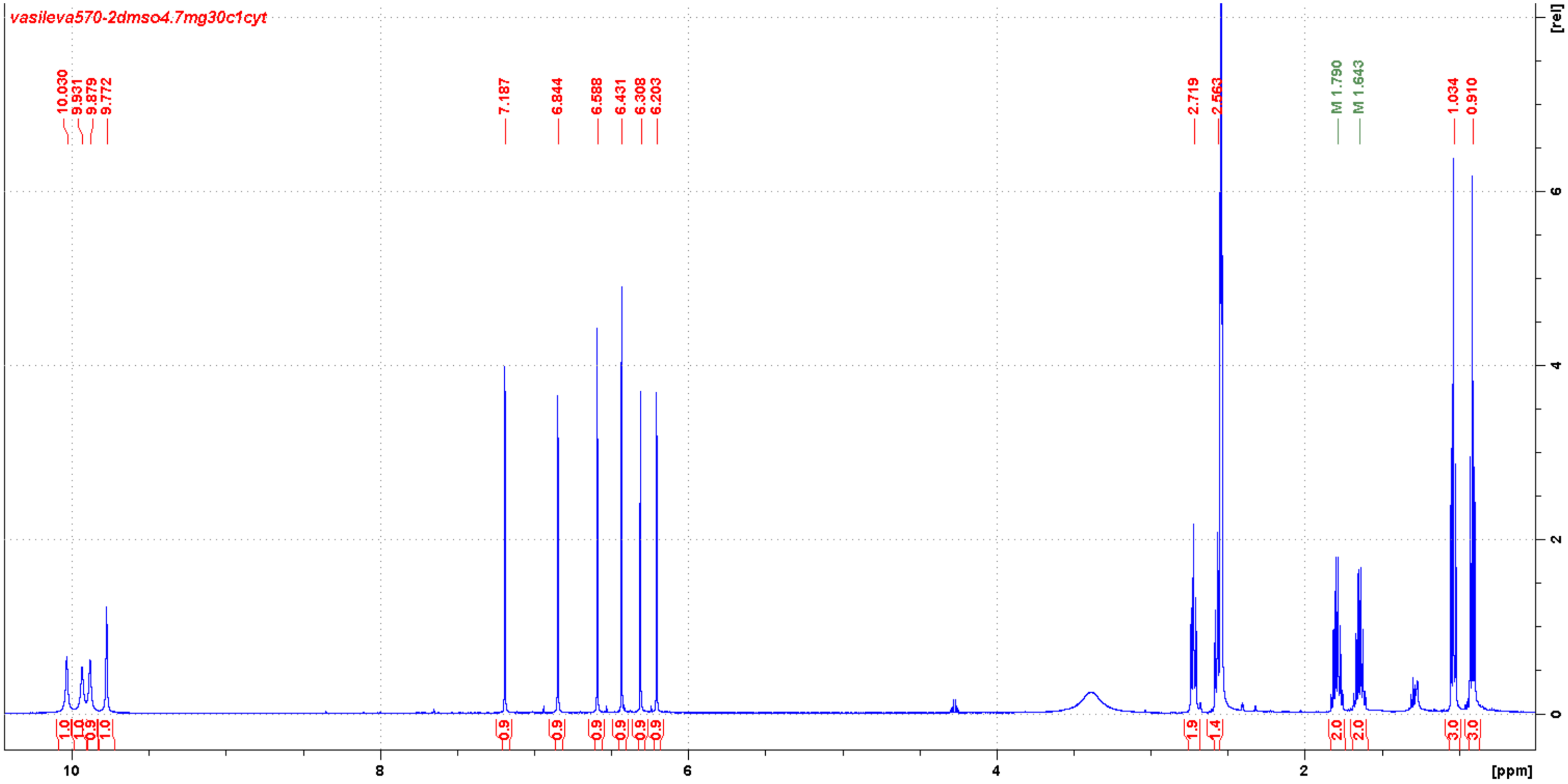

Figure S16. ^{1}H NMR spectrum of **1** (DMSO-*d*$_6$, 500 MHz)

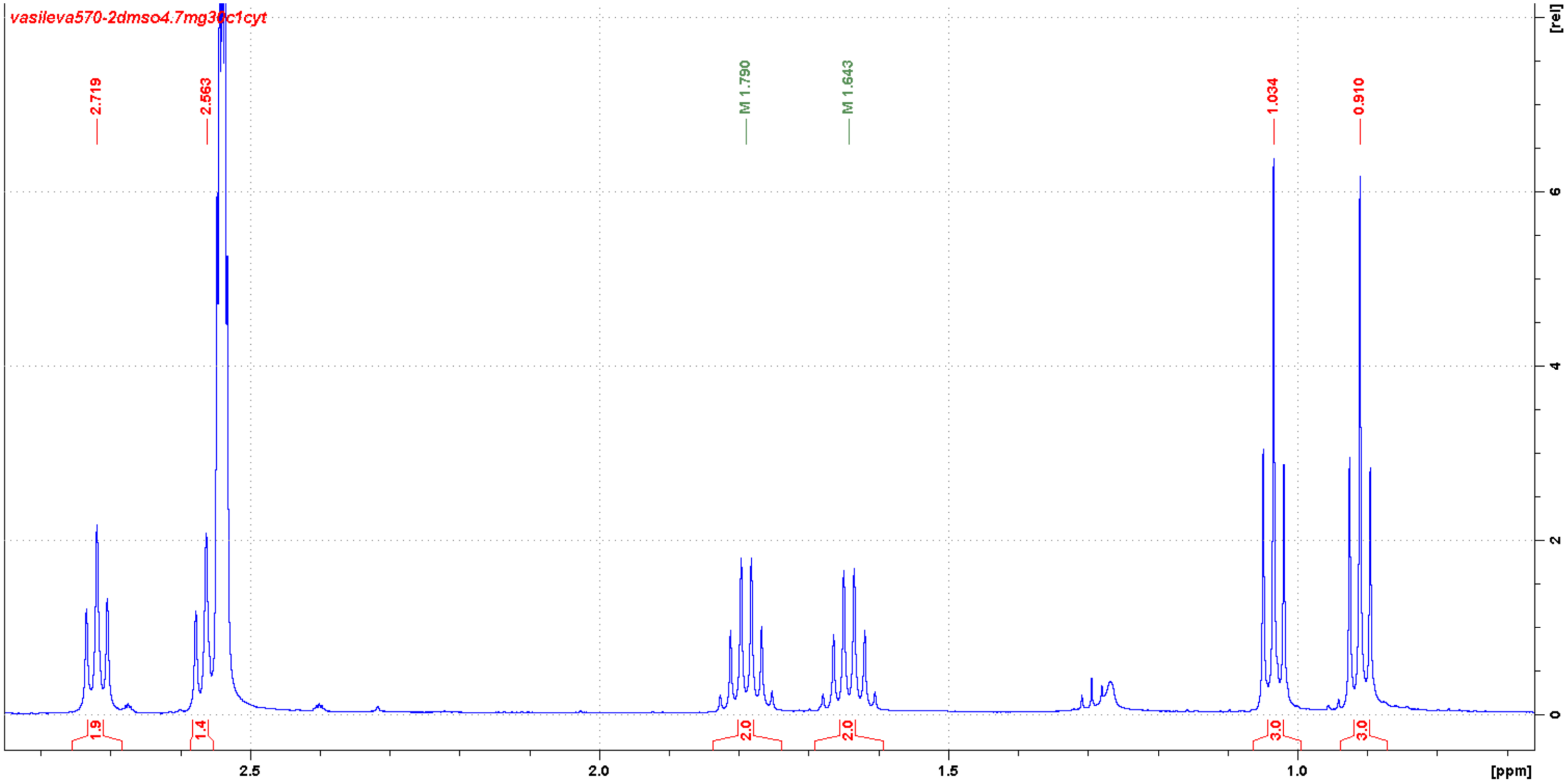

Figure S17. Enlarged fragment of ^{1}H NMR spectrum of **1** (DMSO-d_6, 500 MHz)

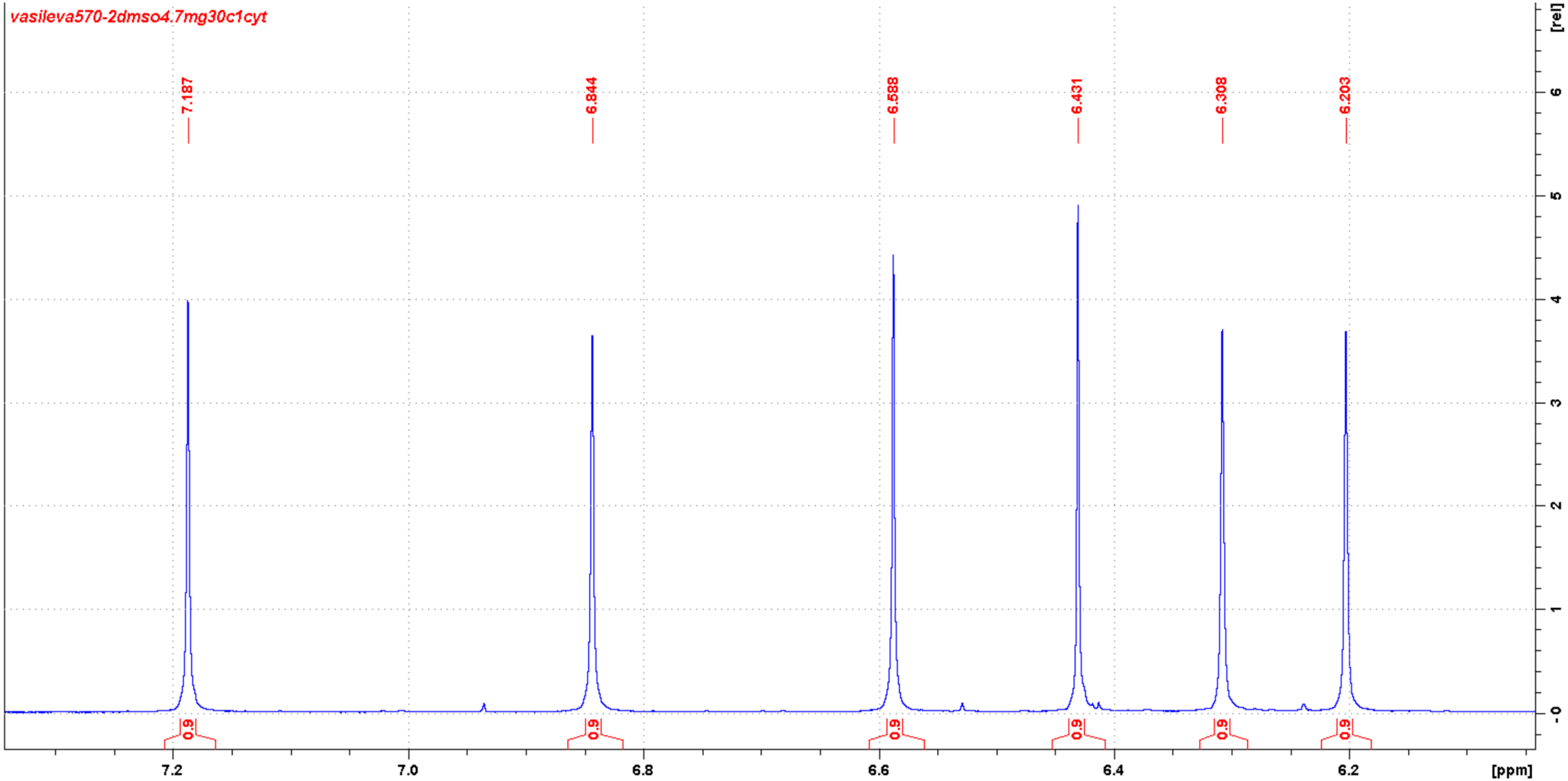

Figure S18. Enlarged fragment of ^{1}H NMR spectrum of **1** (DMSO-d_6, 500 MHz)

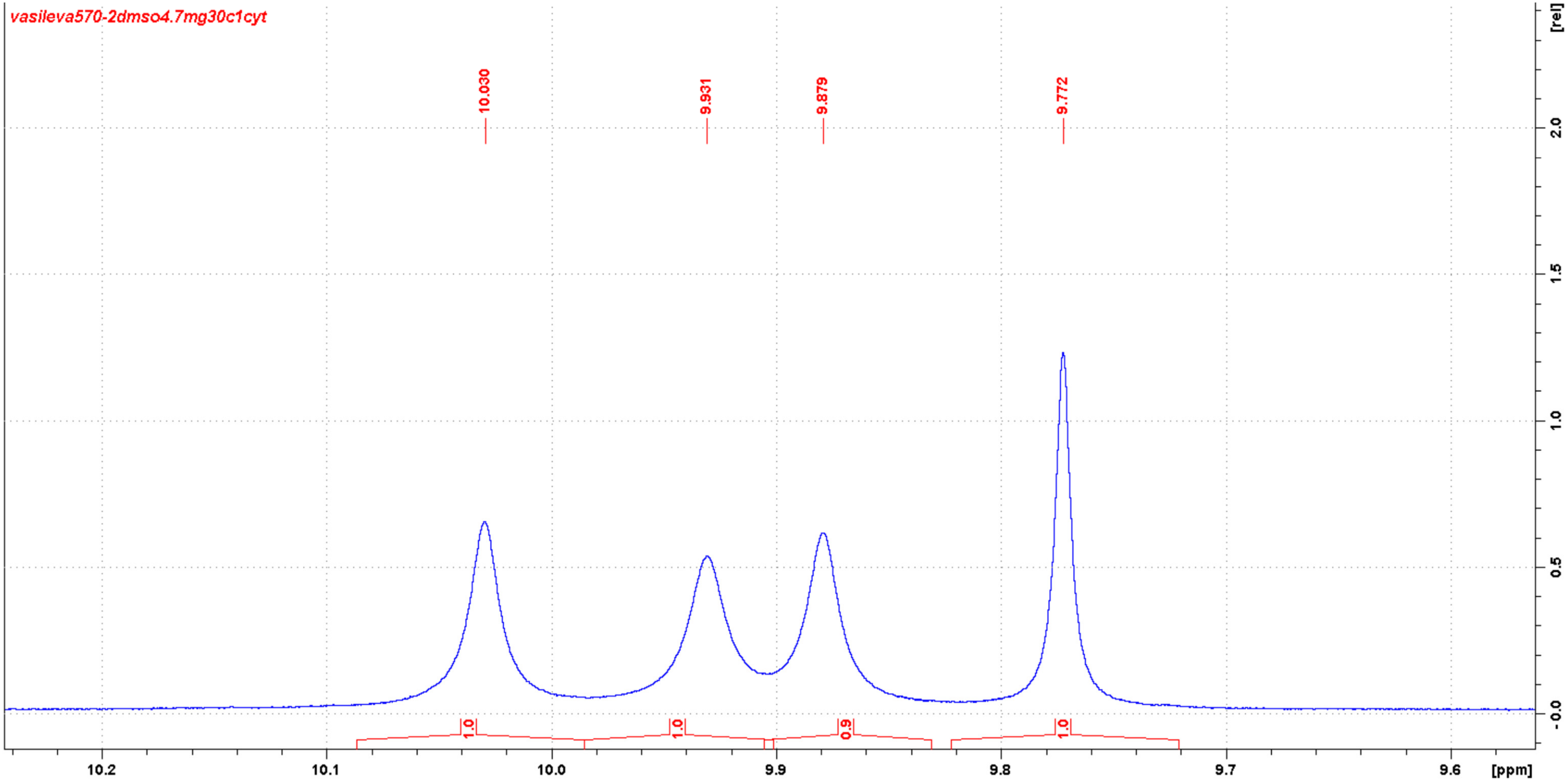

Figure S19. Enlarged fragment of ^{1}H NMR spectrum of **1** (DMSO-d_6, 500 MHz)

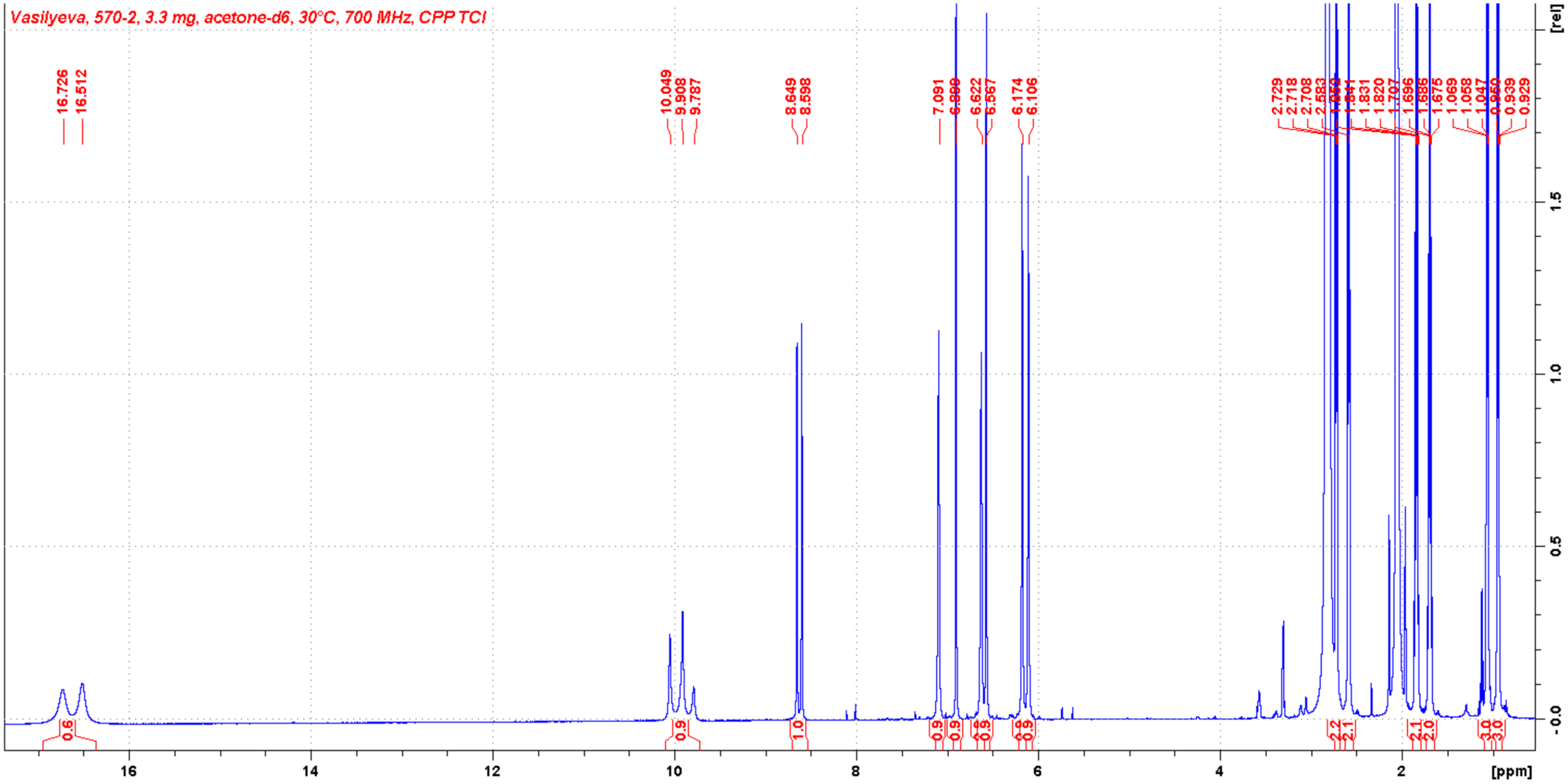

Figure S20. ^{1}H NMR spectrum of **1** (acetone-d_6, 700 MHz)

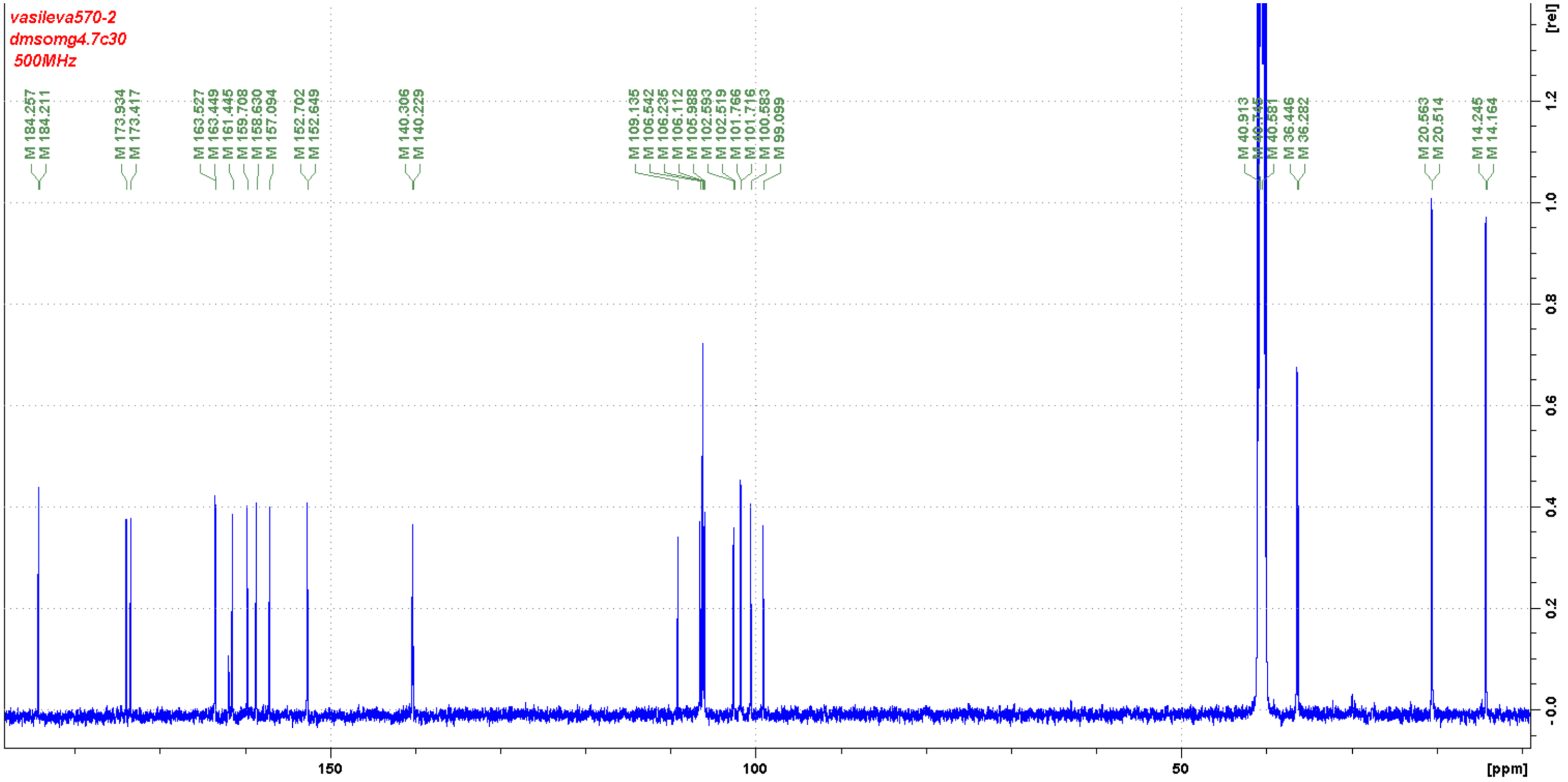

Figure S21. ^{13}C NMR spectrum of **1** (DMSO-d_6, 126 MHz)

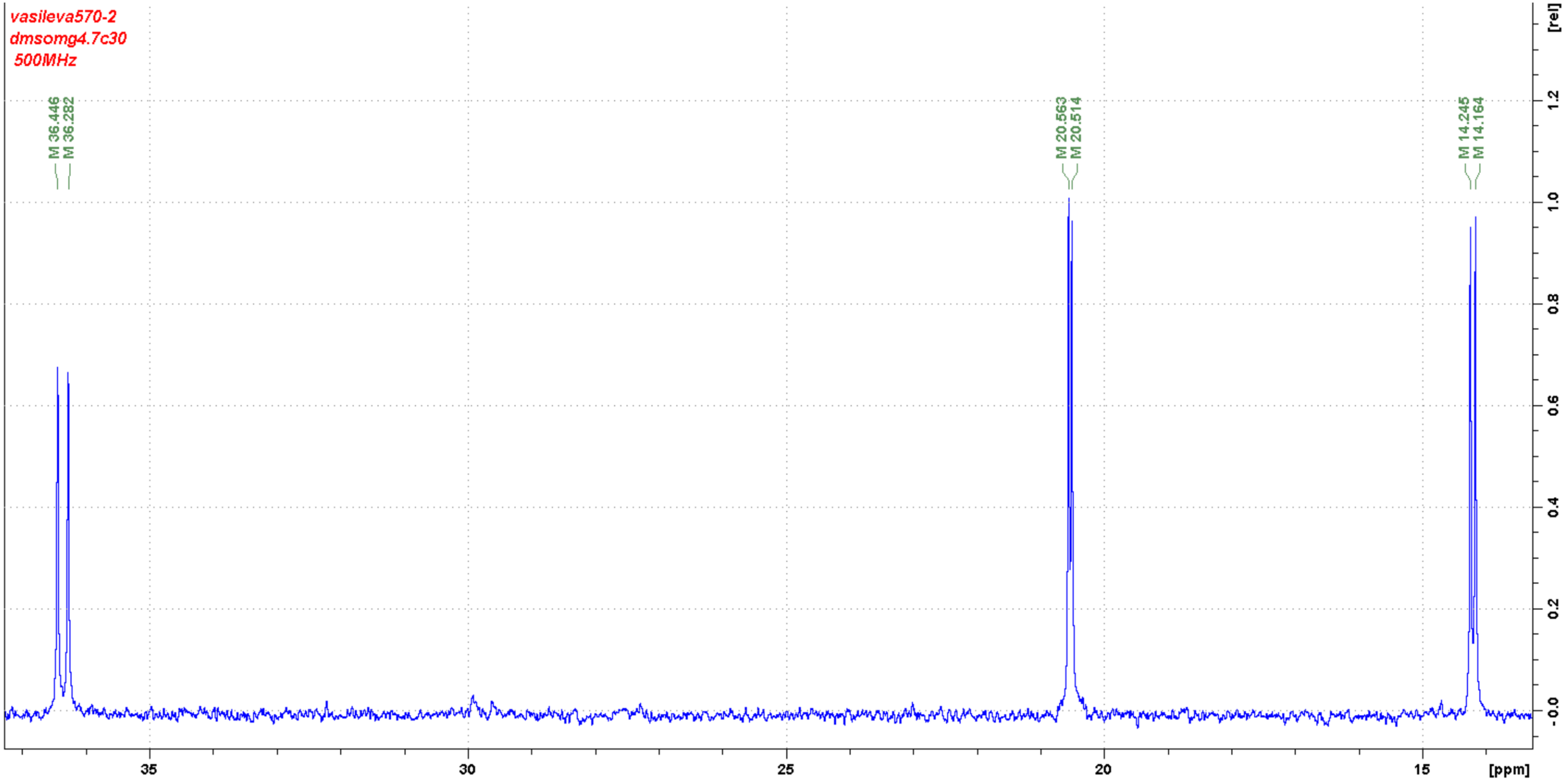

Figure S22. Enlarged fragment of ^{13}C NMR spectrum of **1** (DMSO-d_6, 126 MHz)

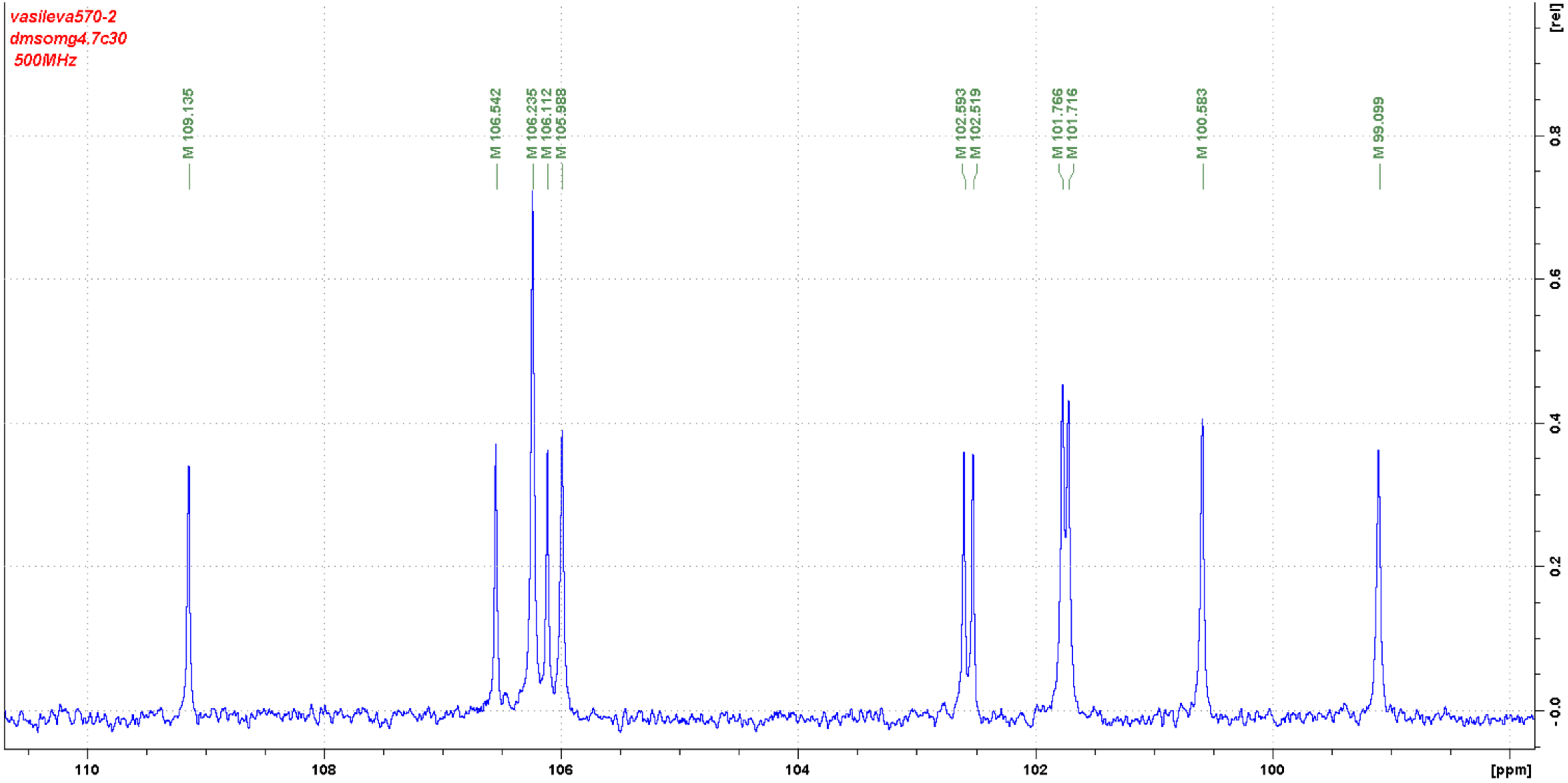

Figure S23. Enlarged fragment of ^{13}C NMR spectrum of **1** (DMSO-d_6, 126 MHz)

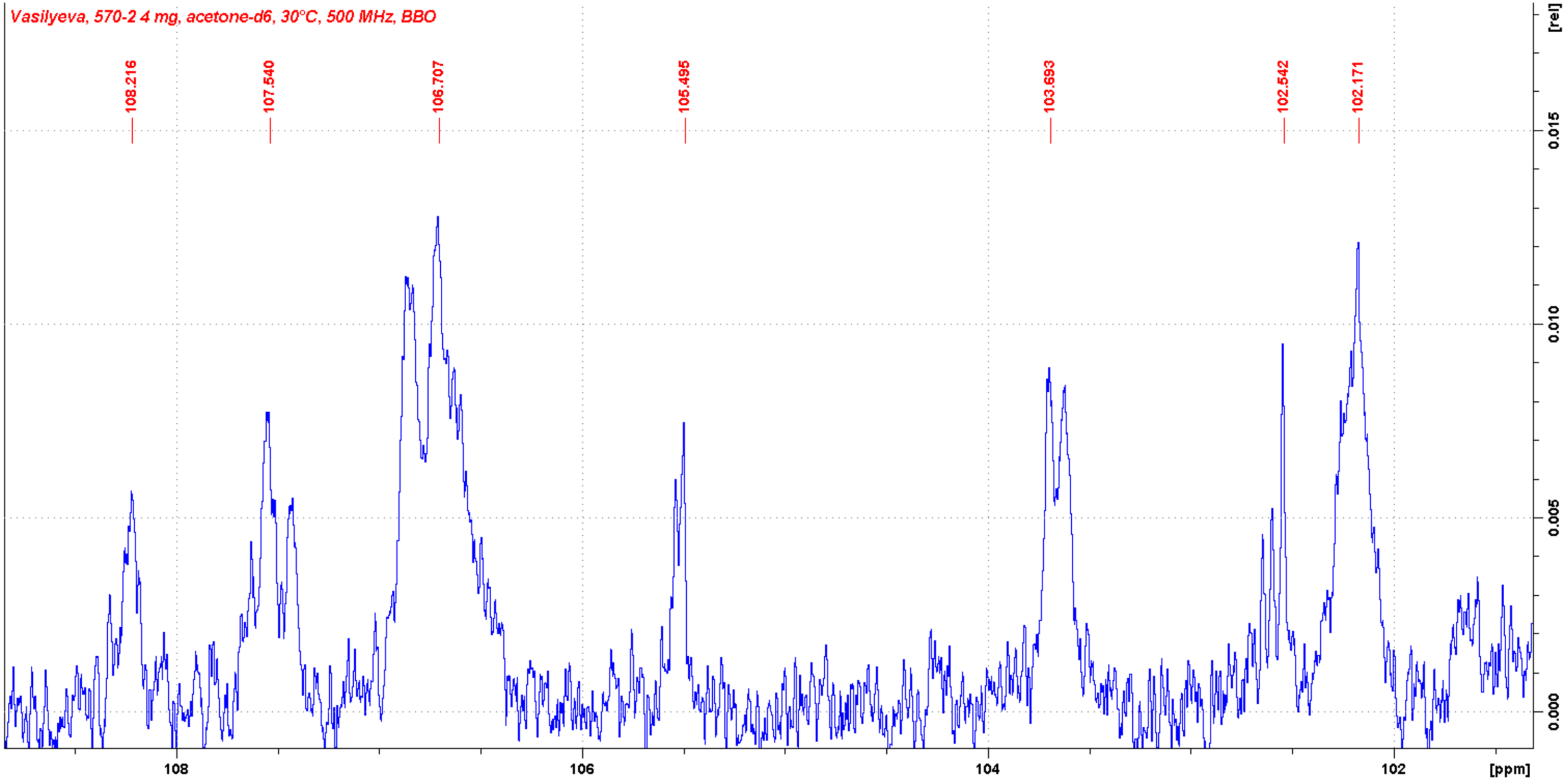

Figure S24. Enlarged fragment of ^{13}C NMR spectrum of **1** (acetone-d_6, 126 MHz). Aromatic carbon signals were poorly resolved

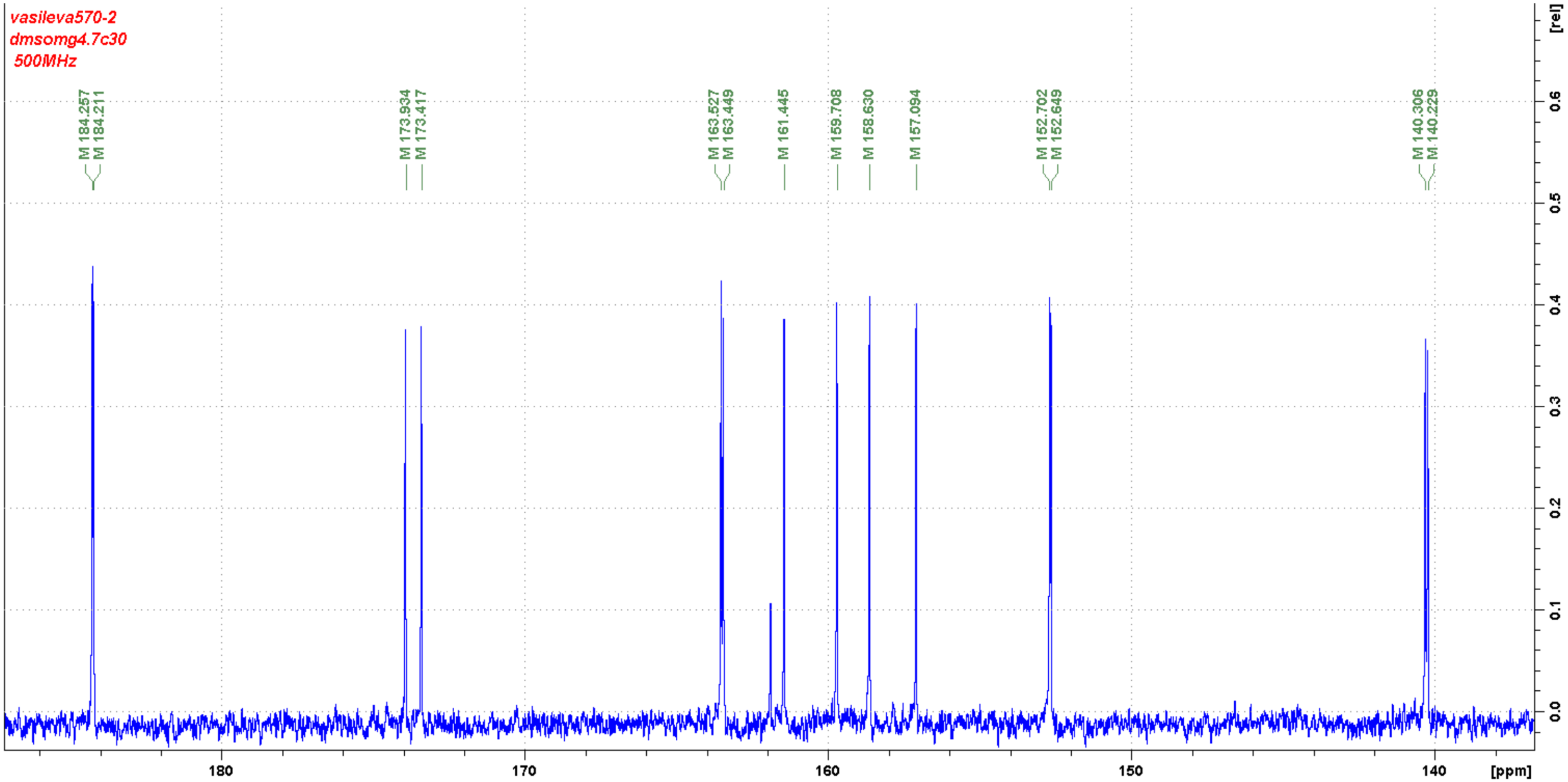

Figure S25. Enlarged fragment of ^{13}C NMR spectrum of **1** (DMSO-d_6, 126 MHz)

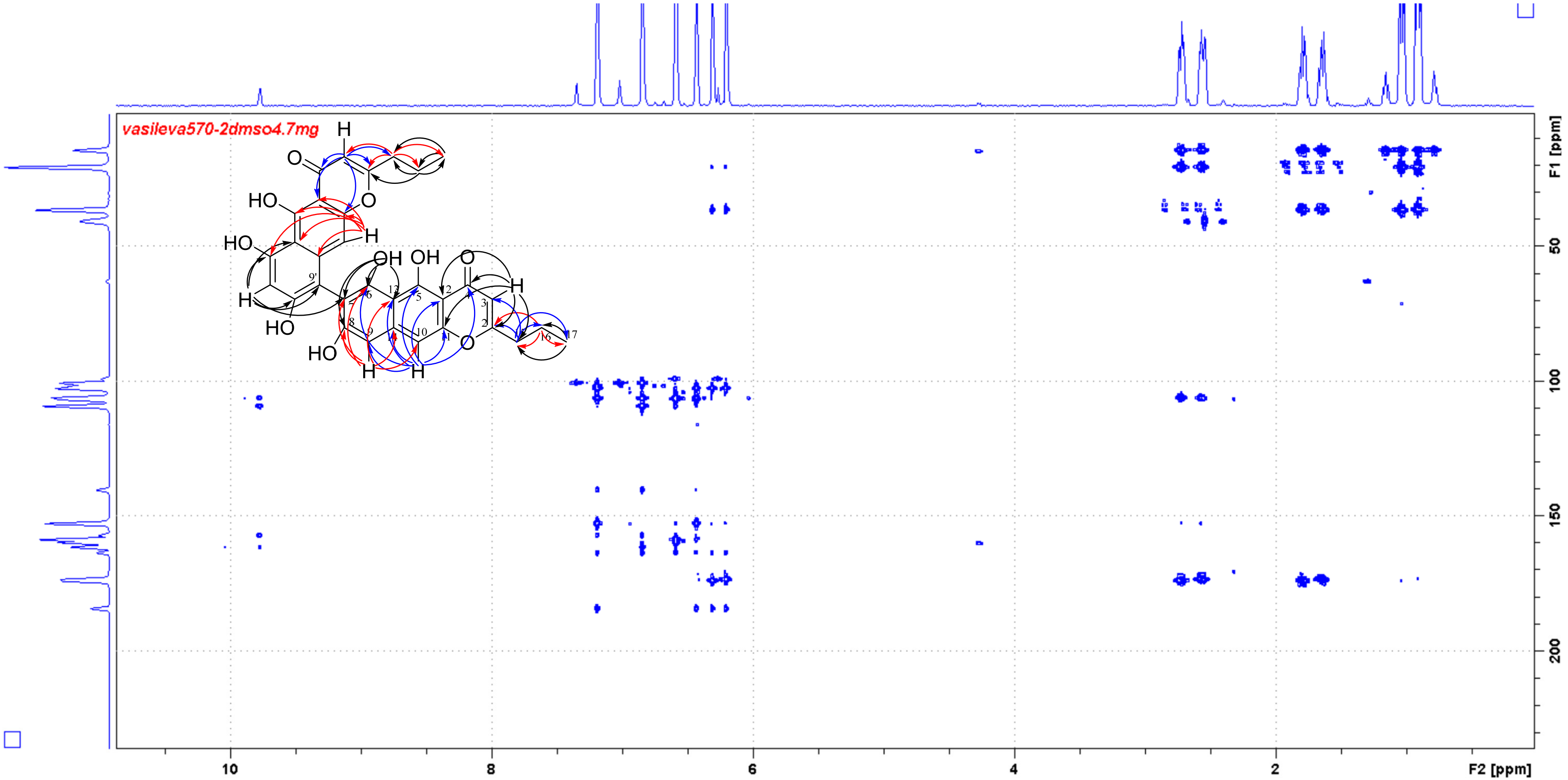

Figure S26. HMBC correlations for **1.** (HMBC correlations for other compounds are very similar)

Table S1. Crystal data and structure refinement for **1**

Empirical formula	$2(C_{32}H_{26}O_{10})\cdot CHCl_3$
Formula weight	1260.42
Temperature	120(2) K
Wavelength	Mo K_α (λ = 0.71073 Å)
Crystal system	Triclinic
Space group	$P\bar{1}$
Unit cell dimensions	a = 11.4196(15) Å, α = 88.977(9)°<br>b = 16.082(3) Å, β= 71.176(6)°<br>c = 16.684(2) Å, γ = 71.034(7)°
Volume	2729.8(7) $Å^3$
Z	2
Dcal	1.533 g/cm^3
μ	0.254 mm^{-1}
F(000)	1308
Crystal size	0.17×0.08×0.02 mm^3
Data collection area in θ, deg.	1.296 – 26.447
Index ranges	–14< h <14, –10< k< 18, –20< l < 20
Reflections collected	18836
Independent reflections	10145 [R(int) = 0.0929]
Data / restraints / parameters	10145 / 39 / 853
Goodness-of-fit on F^2	0.922
Final R indices [I>2sigma(I)]	R1 = 0.0722, wR2 = 0.1309
R indices (all data)	R1 = 0.2337, wR2 = 0.1867
Residual electron density (min/max),	–0.292 / 0.351 e/$Å^3$

Table S2. Selected bond lengths in the structure of **1**

Bond	d, Å	Bond	d, Å
C(1)—C(2)	1.354(8)	C(36)—O(12)	1.344(7)
C(1)—O(5)	1.378(6)	C(36)—C(37)	1.402(8)
C(1)—C(14)	1.478(7)	C(37)—C(42)	1.424(8)
C(2)—C(3)	1.403(7)	C(37)—C(38)	1.430(7)
C(2)—H(2)	0.9500	C(38)—O(13)	1.346(6)
C(3)—O(1)	1.260(6)	C(38)—C(39)	1.396(8)
C(3)—C(4)	1.454(7)	C(39)—C(40)	1.419(8)
C(4)—C(5)	1.390(7)	C(39)—C(48)	1.486(7)
C(4)—C(13)	1.406(7)	C(40)—O(14)	1.347(6)
C(5)—O(2)	1.350(6)	C(40)—C(41)	1.371(7)
C(5)—C(6)	1.398(7)	C(41)—C(42)	1.414(7)
C(6)—C(7)	1.430(7)	C(41)—H(41)	0.9500
C(6)—C(11)	1.437(7)	C(42)—C(43)	1.435(7)
C(7)—C(8)	1.365(7)	C(43)—C(44)	1.346(7)
C(7)—O(3)	1.366(6)	C(43)—H(43)	0.9500
C(8)—C(9)	1.378(7)	C(44)—O(15)	1.382(6)
C(8)—H(8)	0.9500	C(45)—C(46)	1.503(7)
C(9)—O(4)	1.372(6)	C(45)—H(45A)	0.9900
C(9)—C(10)	1.386(7)	C(45)—H(45B)	0.9900
C(10)—C(11)	1.414(7)	C(46)—C(47)	1.529(7)
C(10)—C(17)	1.492(7)	C(46)—H(46A)	0.9900
C(11)—C(12)	1.425(7)	C(46)—H(46B)	0.9900
C(12)—C(13)	1.360(7)	C(47)—H(47A)	0.9800
C(12)—H(12)	0.9500	C(47)—H(47B)	0.9800
C(13)—O(5)	1.391(6)	C(47)—H(47C)	0.9800
C(14)—C(15)	1.529(9)	C(48)—C(60)	1.377(7)
C(14)—H(14A)	0.9900	C(48)—C(49)	1.412(7)

C(14)—H(14B)	0.9900	C(49)—C(50)	1.414(7)
C(15)—C(16)	1.570(10)	C(49)—C(57)	1.429(7)
C(15)—H(15A)	0.9900	C(50)—C(51)	1.354(7)
C(15)—H(15B)	0.9900	C(50)—H(50)	0.9500
C(16)—H(16A)	0.9800	C(51)—O(16)	1.393(6)
C(16)—H(16B)	0.9800	C(51)—C(55)	1.399(7)
C(16)—H(16C)	0.9800	C(52)—C(53)	1.322(8)
C(17)—C(28)	1.378(7)	C(52)—O(16)	1.370(6)
C(17)—C(18)	1.413(8)	C(52)—C(61B)	1.533(12)
C(18)—C(19)	1.364(7)	C(52)—C(61A)	1.551(11)
C(18)—O(6)	1.380(6)	C(53)—C(54)	1.424(8)
C(19)—C(20)	1.425(7)	C(53)—H(53)	0.9500
C(19)—H(19)	0.9500	C(54)—O(17)	1.264(6)
C(20)—C(21)	1.416(7)	C(54)—C(55)	1.444(7)
C(20)—C(27)	1.422(7)	C(55)—C(56)	1.391(7)
C(21)—C(22)	1.377(7)	C(56)—O(18)	1.363(6)
C(21)—H(21)	0.9500	C(56)—C(57)	1.414(7)
C(22)—O(7)	1.370(6)	C(57)—C(58)	1.430(7)
C(22)—C(26)	1.402(8)	C(58)—C(59)	1.353(7)
C(23)—C(24)	1.323(8)	C(58)—O(19)	1.368(6)
C(23)—O(7)	1.381(6)	C(59)—C(60)	1.408(7)
C(23)—C(29)	1.493(7)	C(59)—H(59)	0.9500
C(24)—C(25)	1.430(7)	C(60)—O(20)	1.358(6)
C(24)—H(24)	0.9500	O(12)—H(12A)	0.8400
C(25)—O(8)	1.261(7)	O(13)—H(13)	0.8400
C(25)—C(26)	1.437(8)	O(14)—H(14)	0.8400
C(26)—C(74)	1.398(7)	O(18)—H(18)	0.8400
C(27)—C(74)	1.412(7)	O(19)—H(19A)	0.8400
C(27)—C(28)	1.450(7)	O(20)—H(20)	0.8400

C(28)—O(10)	1.352(6)	C(61A)—C(62A)	1.524(14)
C(29)—C(30)	1.512(7)	C(61A)—H(61A)	0.9900
C(29)—H(29A)	0.9900	C(61A)—H(61B)	0.9900
C(29)—H(29B)	0.9900	C(62A)—C(63A)	1.520(15)
C(30)—C(31)	1.522(7)	C(62A)—H(62A)	0.9900
C(30)—H(30A)	0.9900	C(62A)—H(62B)	0.9900
C(30)—H(30B)	0.9900	C(63A)—H(63A)	0.9800
C(31)—H(31A)	0.9800	C(63A)—H(63B)	0.9800
C(31)—H(31B)	0.9800	C(63A)—H(63C)	0.9800
C(31)—H(31C)	0.9800	C(61B)—C(62B)	1.525(13)
C(74)—O(9)	1.337(6)	C(61B)—H(61C)	0.9900
O(2)—H(2A)	0.8400	C(61B)—H(61D)	0.9900
O(3)—H(3)	0.8400	C(62B)—C(63B)	1.507(15)
O(4)—H(4)	0.8400	C(62B)—H(62C)	0.9900
O(6)—H(6)	0.8400	C(62B)—H(62D)	0.9900
O(9)—H(9)	0.8400	C(63B)—H(63D)	0.9800
O(10)—H(10)	0.8400	C(63B)—H(63E)	0.9800
C(32)—C(33)	1.333(8)	C(63B)—H(63F)	0.9800
C(32)—O(15)	1.368(6)	C(64)—Cl(1)	1.730(7)
C(32)—C(45)	1.491(7)	C(64)—Cl(3A)	1.738(7)
C(33)—C(34)	1.433(7)	C(64)—Cl(2B)	1.740(7)
C(33)—H(33)	0.9500	C(64)—Cl(2A)	1.785(7)
C(34)—O(11)	1.268(7)	C(64)—Cl(3B)	1.790(7)
C(34)—C(35)	1.418(8)	C(64)—H(64A)	1.0000
C(35)—C(36)	1.400(7)	C(64)—H(64B)	1.0000
C(35)—C(44)	1.428(8)		

Table S3. Hydrogen bonds in the structure of **1** (Å and °)

D—H···A	d(D—H)	d(H···A)	d(D···A)	<(DHA)
C(2)—H(2)···O(20)^{i}	0.95	2.50	3.426(6)	163.9
C(8)—H(8)···O(14)	0.95	2.64	3.415(6)	138.6
C(21)—H(21)···O(13)^{ii}	0.95	2.40	3.326(7)	165.4
O(2)—H(2A)···O(1)	0.84	1.75	2.506(5)	149.4
O(3)—H(3)···O(2)	0.84	1.87	2.597(5)	143.5
O(4)—H(4)···O(14)	0.84	2.43	3.131(5)	142.0
O(4)—H(4)···O(17)^{iii}	0.84	2.49	3.013(5)	121.0
O(6)—H(6)···O(1)^{i}	0.84	1.77	2.603(5)	172.6
O(10)—H(10)···O(9)	0.84	1.85	2.604(5)	147.9
C(43)—H(43)···O(10)^{iv}	0.95	2.50	3.406(7)	159.4
O(12)—H(12A)···O(11)	0.84	1.71	2.473(5)	150.8
O(13)—H(13)···O(12)	0.84	1.85	2.587(5)	146.5
O(14)—H(14)···O(17)^{iii}	0.84	2.00	2.685(5)	138.4
O(18)—H(18)···O(17)	0.84	1.78	2.532(5)	148.7
O(19)—H(19A)···O(18)	0.84	1.87	2.612(5)	146.1
O(20)—H(20)···O(6)	0.84	1.93	2.740(5)	160.3
C(64)—H(64A)···O(15)^{v}	1.00	2.52	3.377(8)	144.1

Symmetry transformations used to generate equivalent atoms: (i) –x+2,–y,–z+1; (ii) –x+1,–y+1,–z+1; (iii) –x,–y+1,–z+1; (iv) –x+1,–y,–z+1; (v) x,y+1,z

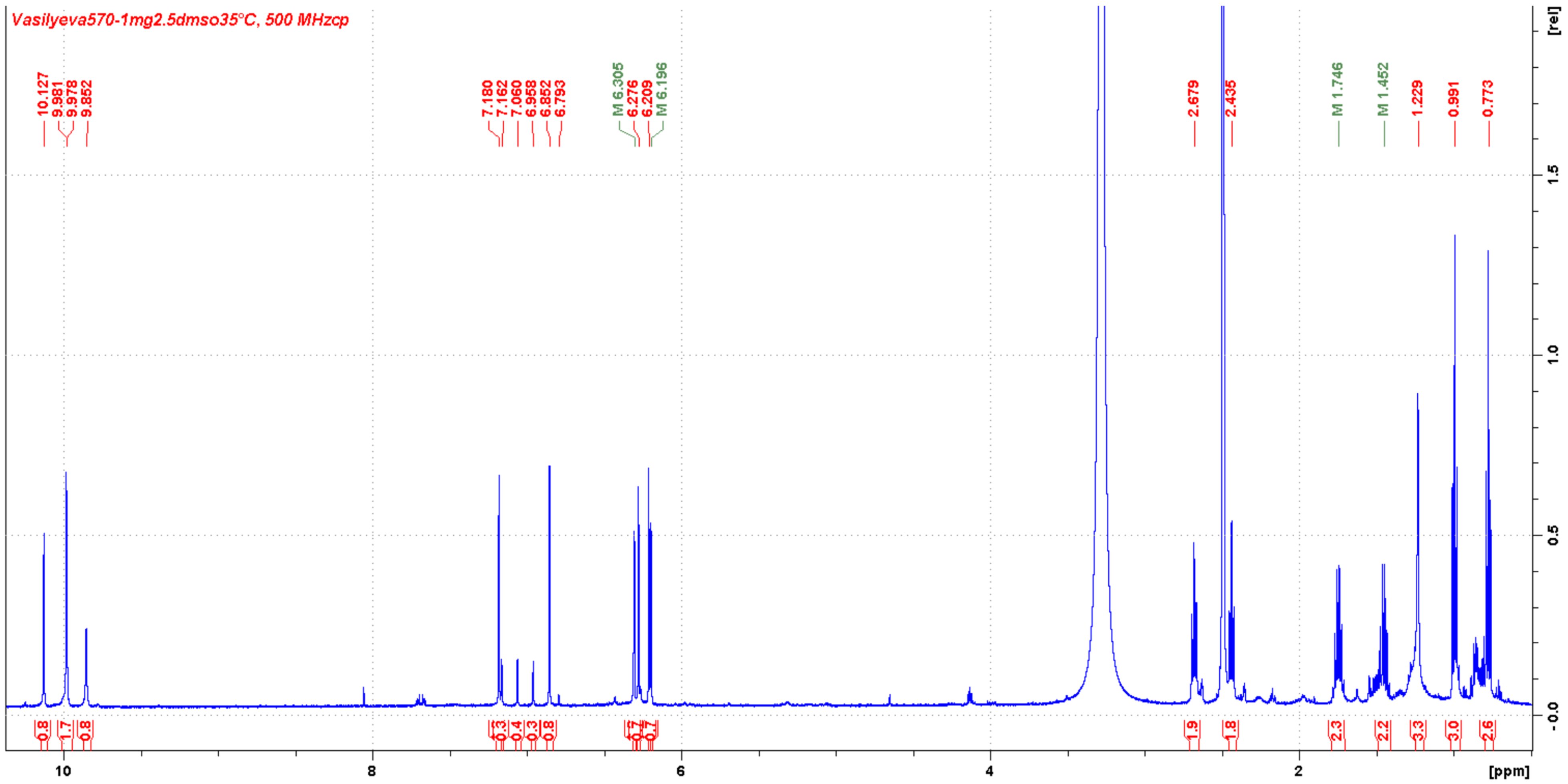

Figure S27. ^{1}H NMR spectrum of **2** (DMSO-d_6, 500 MHz)

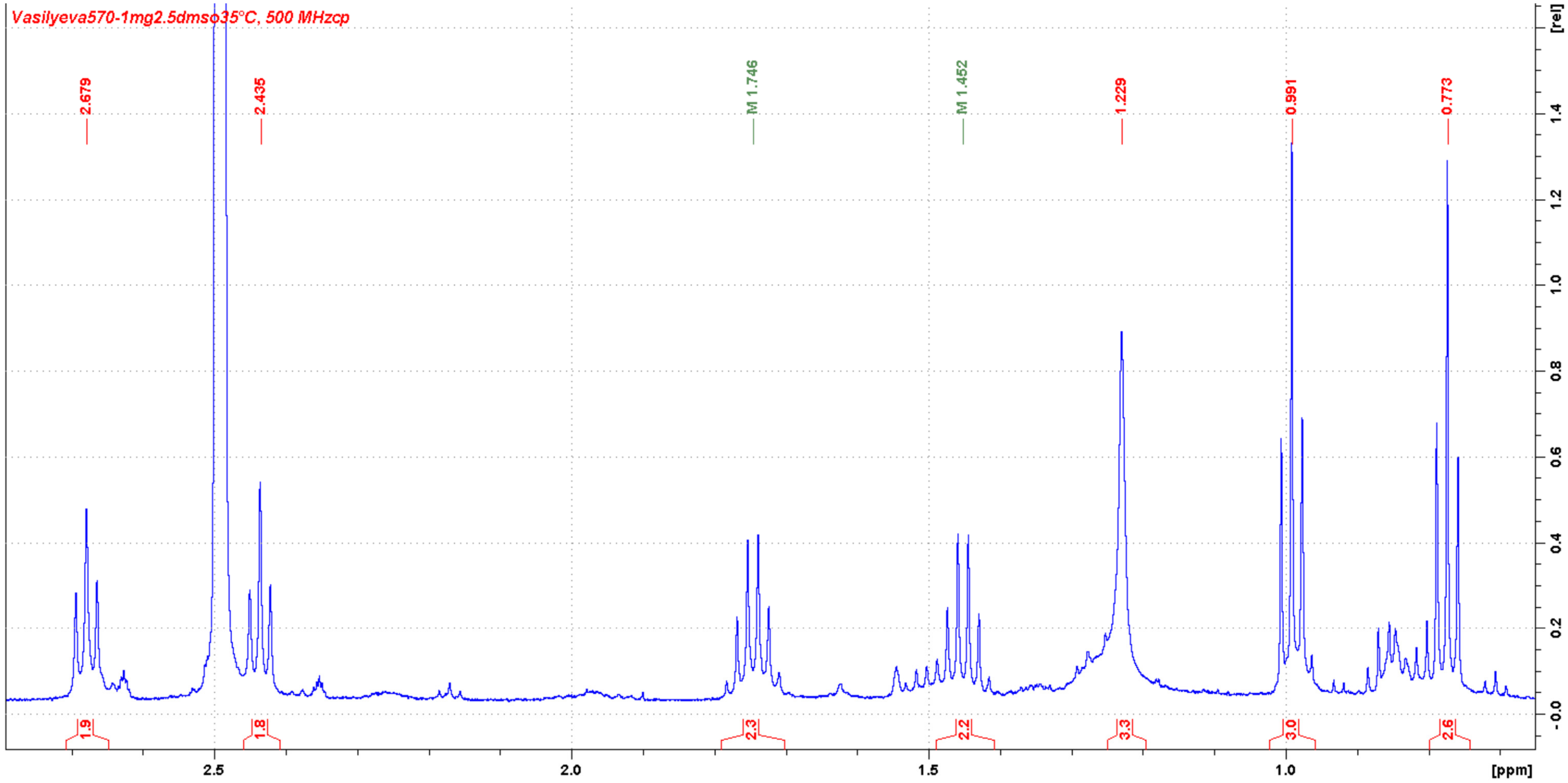

Figure S28. Enlarged fragment of ^{1}H NMR spectrum of **2** (DMSO-d_6, 500 MHz)

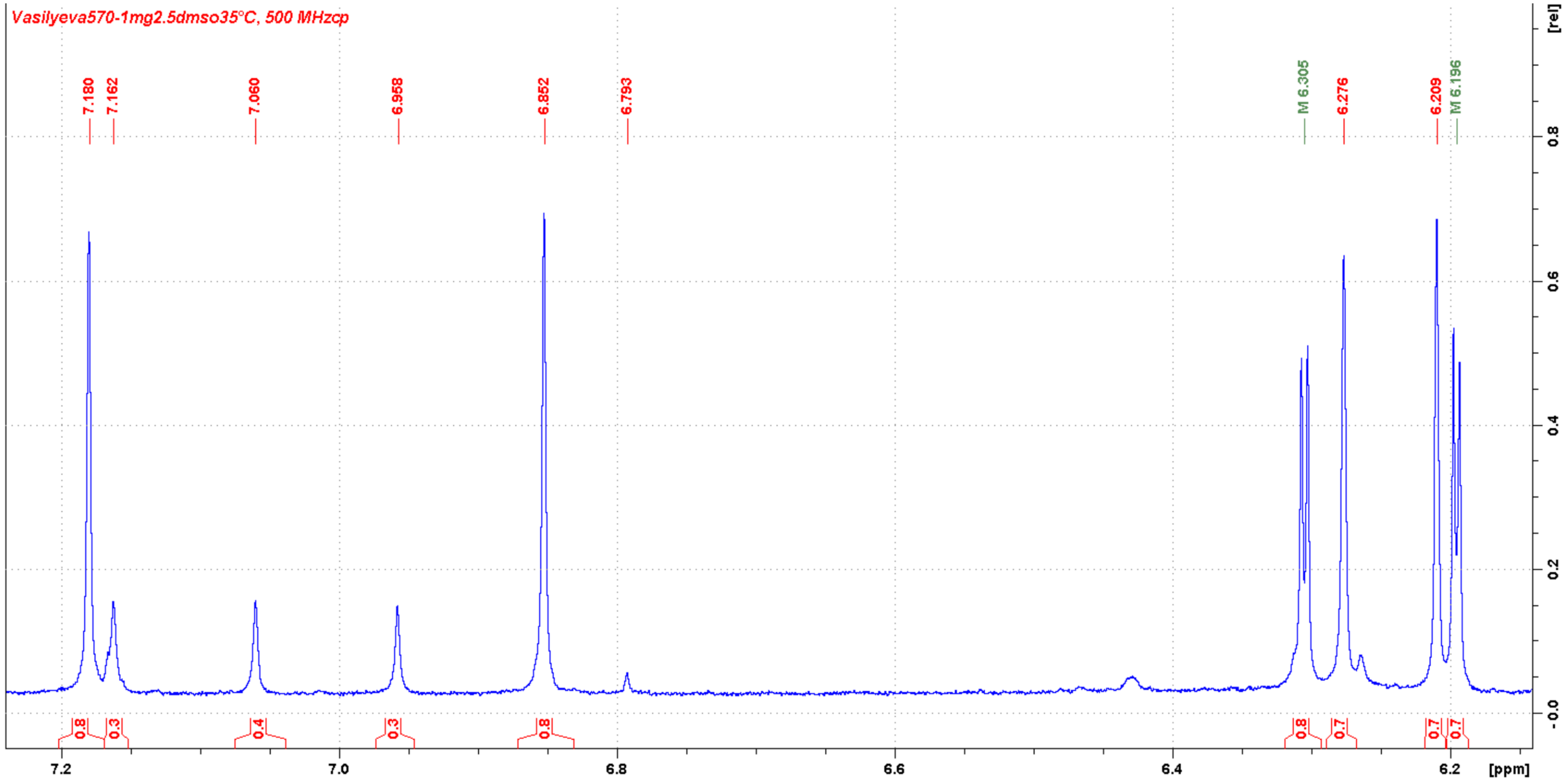

Figure S29. Enlarged fragment of ^{1}H NMR spectrum of **2** (DMSO-d_6, 500 MHz)

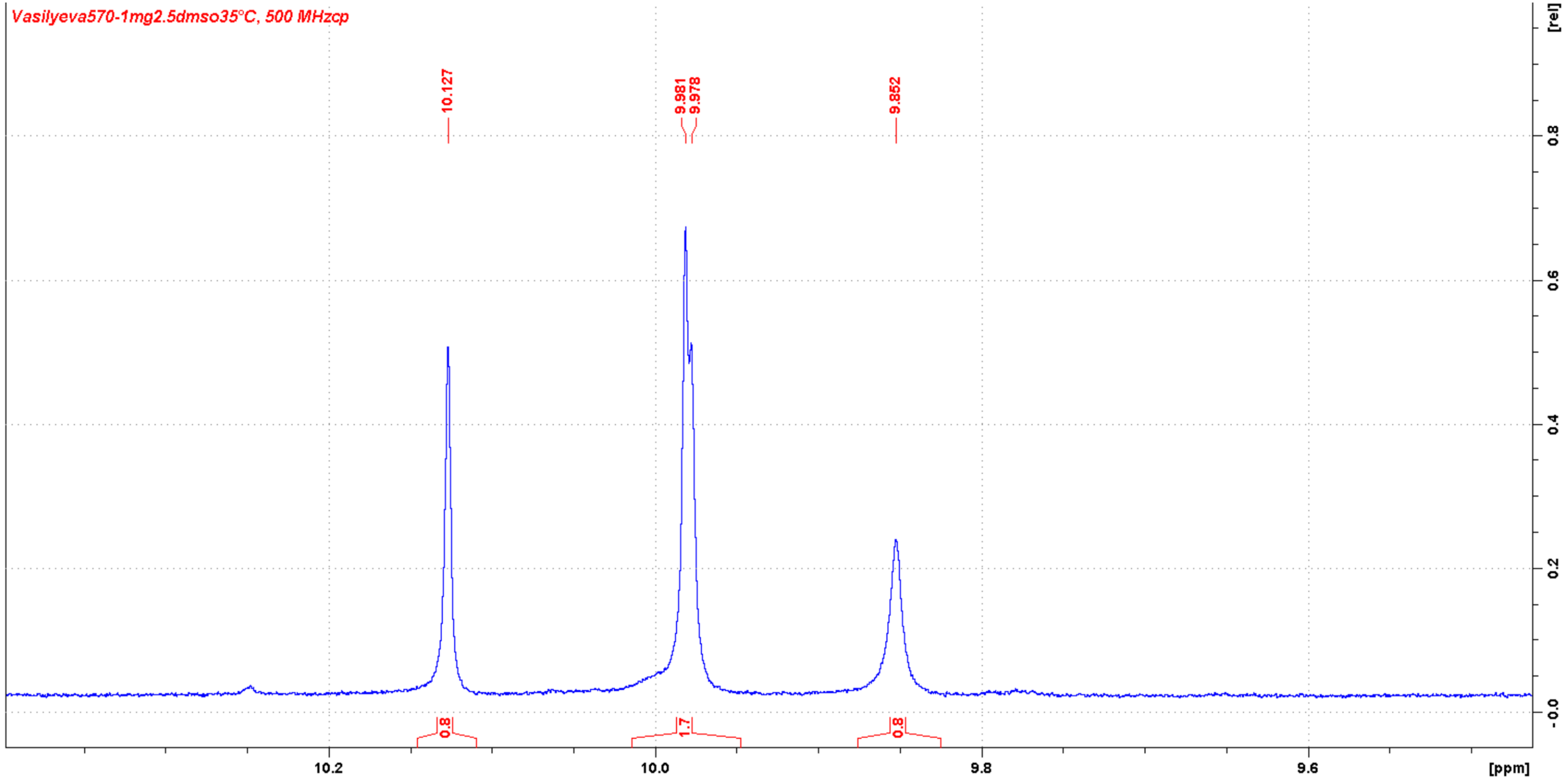

Figure S30. Enlarged fragment of ^{1}H NMR spectrum of **2** (DMSO-d_6, 500 MHz)

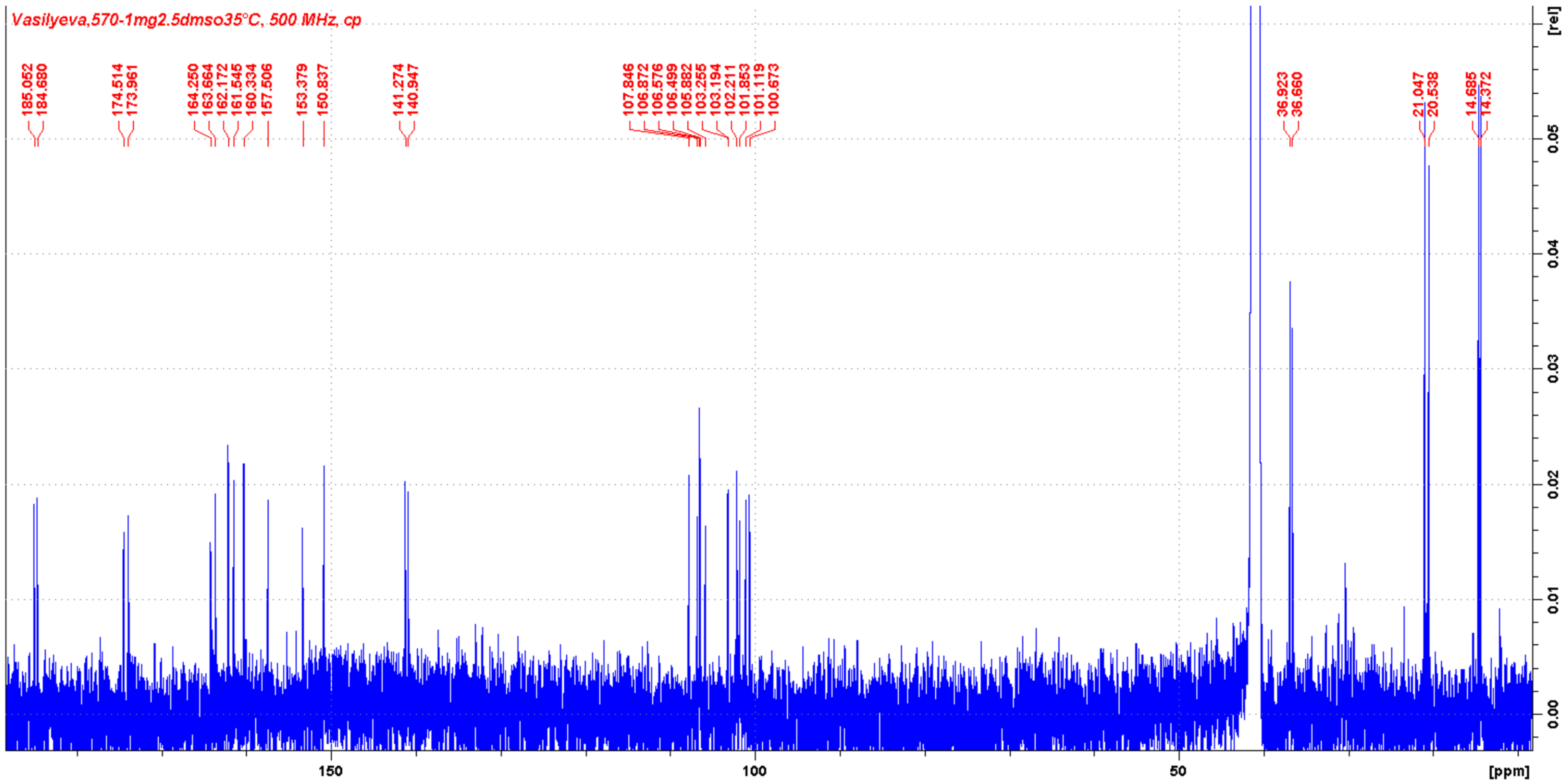

Figure S31. ^{13}C NMR spectrum of **2** (DMSO-d_6, 126 MHz)

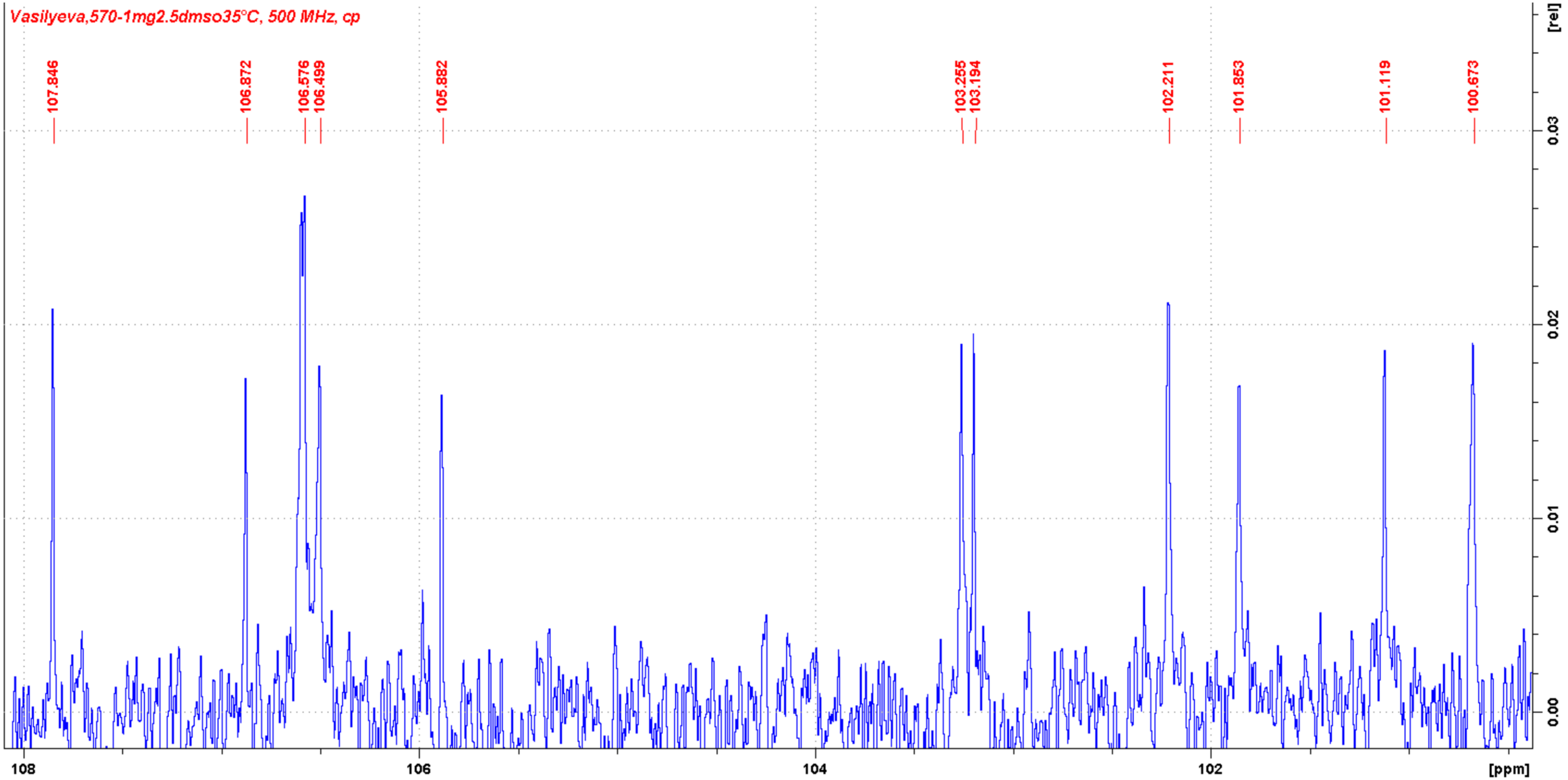

Figure S32. Enlarged fragment of ^{13}C NMR spectrum of **2** (DMSO-*d*$_6$, 126 MHz)

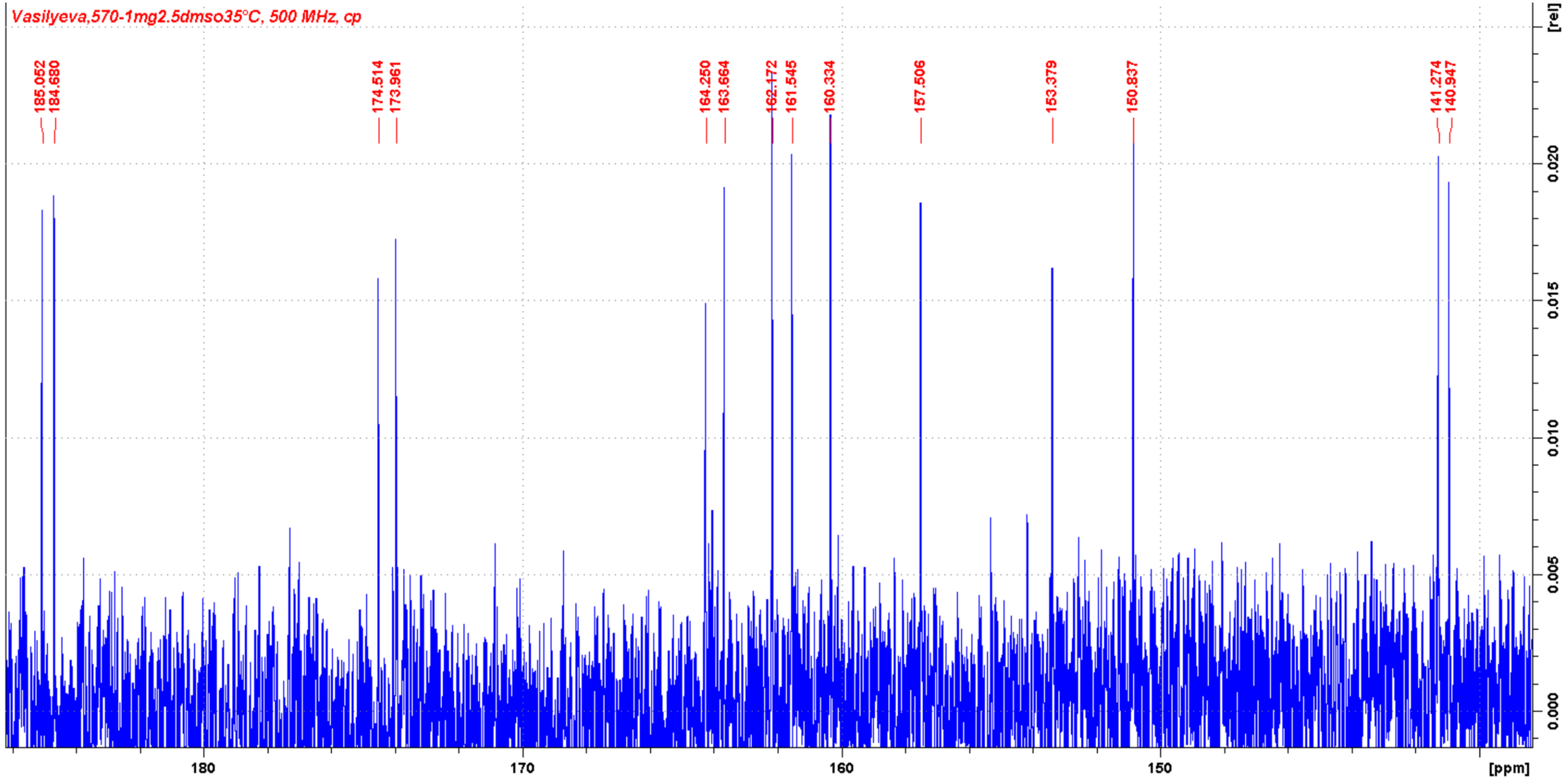

Figure S33. Enlarged fragment of ^{13}C NMR spectrum of **2** (DMSO-d_6, 126 MHz)

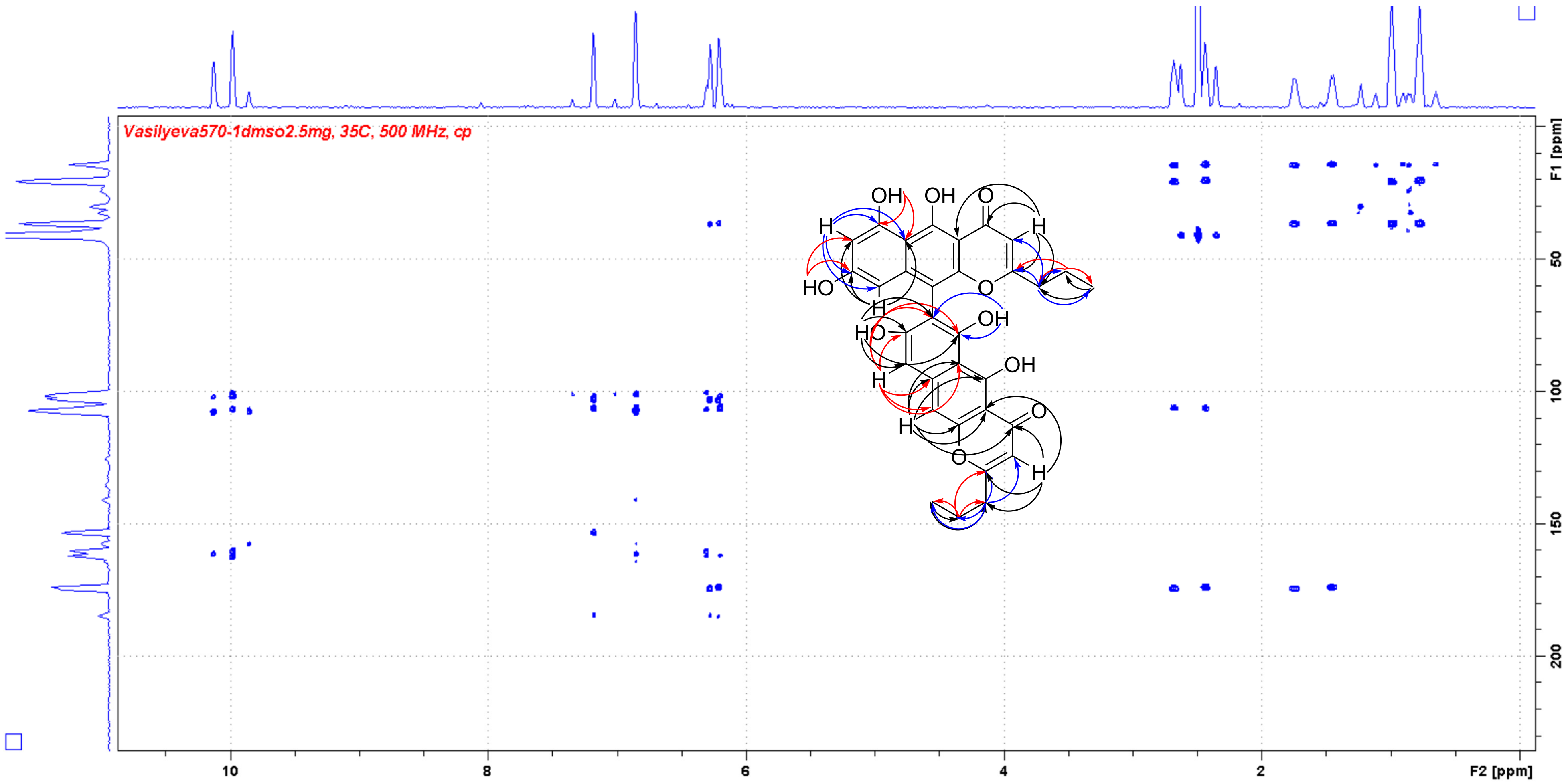

Figure S34. HMBC spectrum of **2**

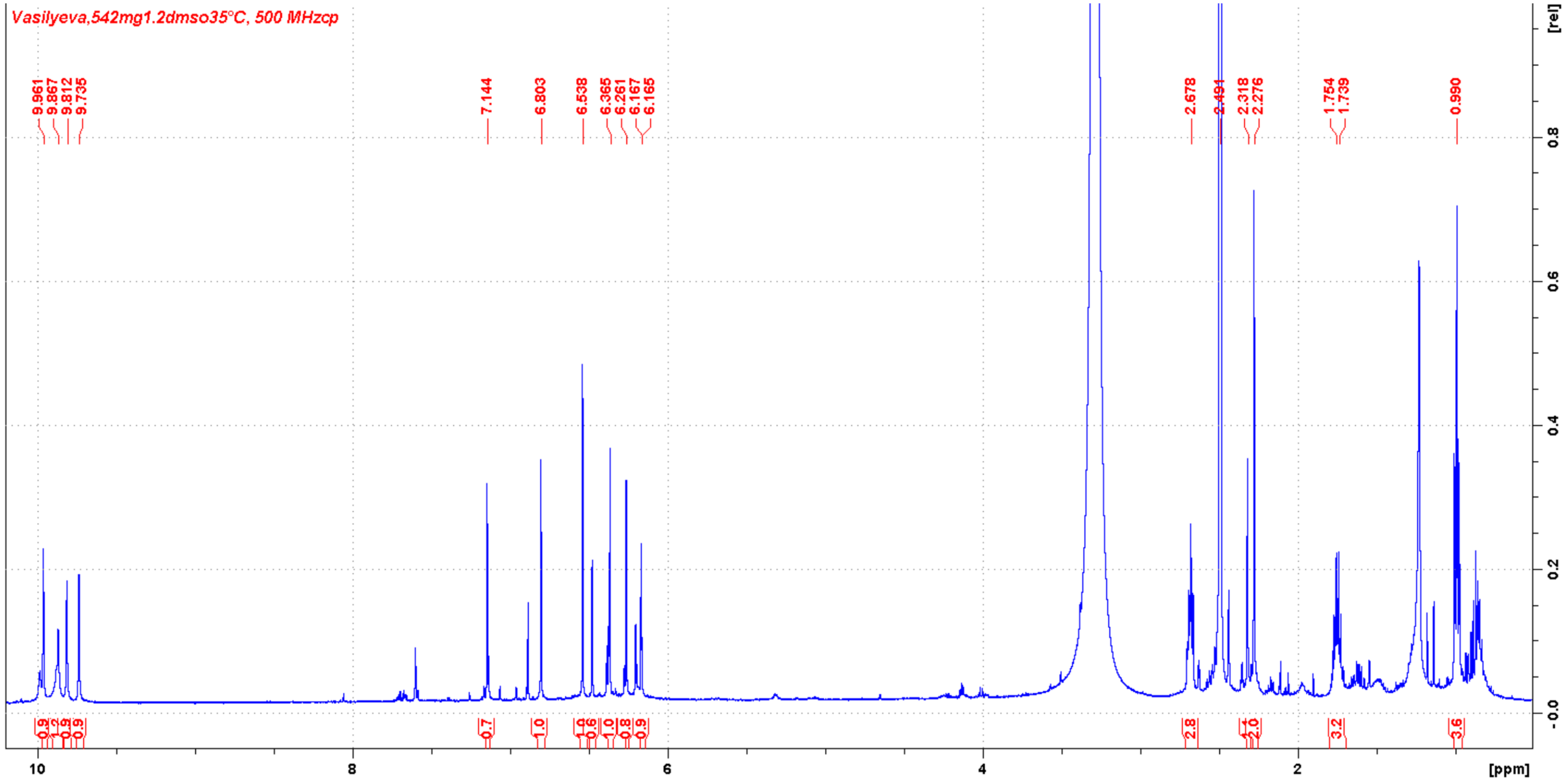

Figure S35. ^{1}H NMR spectrum of **3** (DMSO-d_6, 500 MHz)

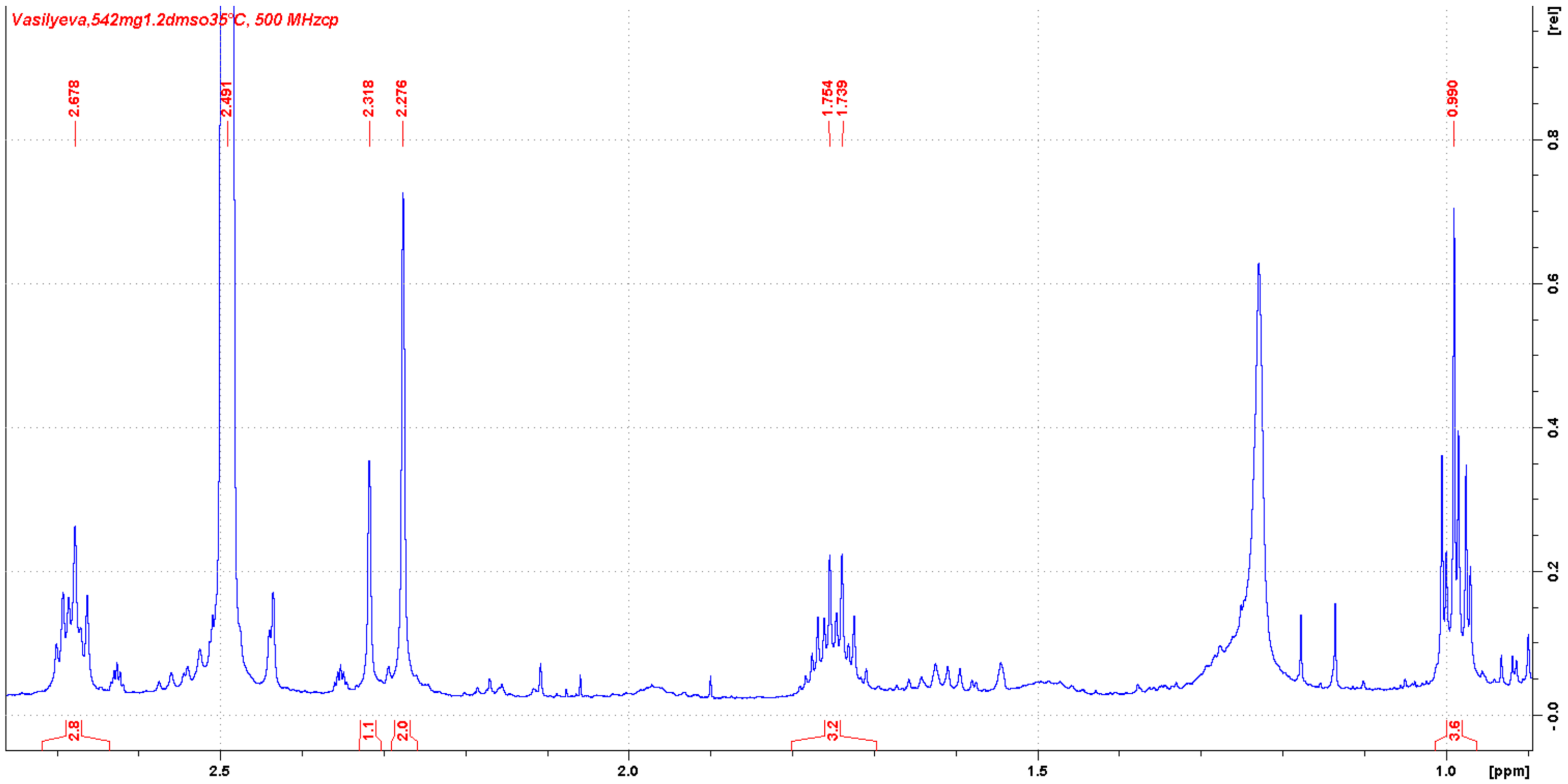

Figure S36. Enlarged fragment of ^{1}H NMR spectrum of **3** (DMSO-d_6, 500 MHz)

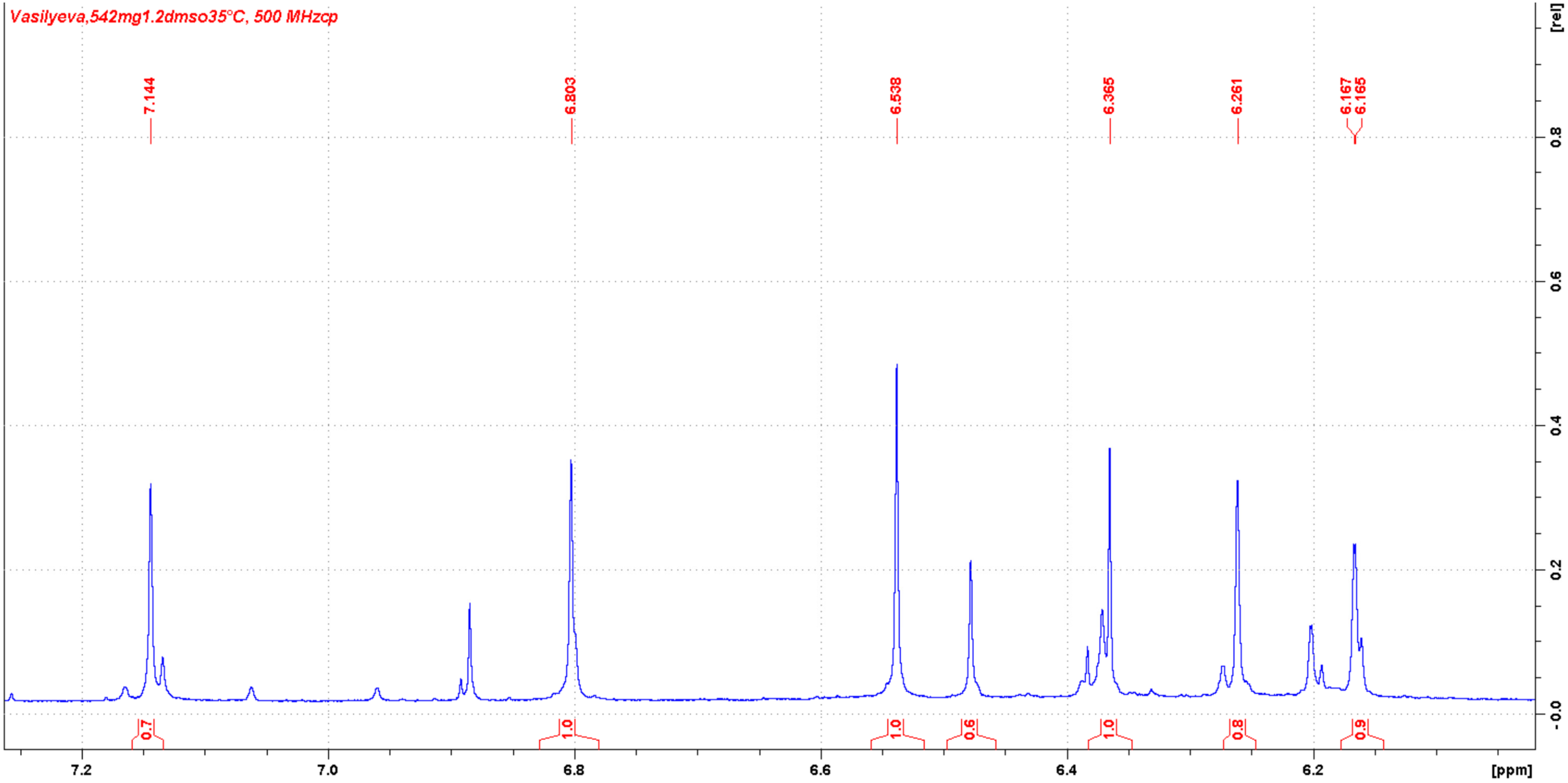

Figure S37. Enlarged fragment of ^{1}H NMR spectrum of **3** (DMSO-d_6, 500 MHz)

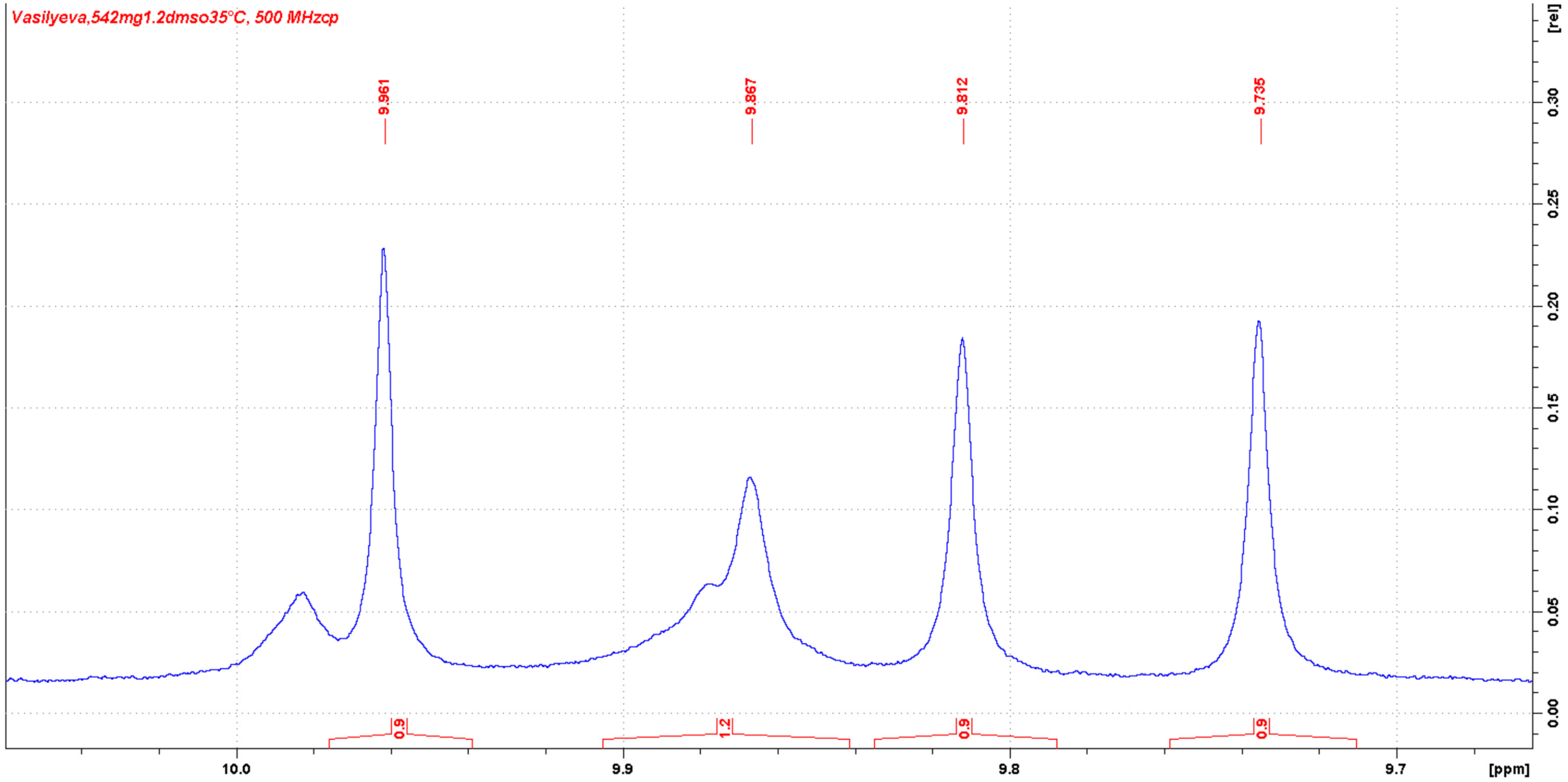

Figure S38. Enlarged fragment of ^{1}H NMR spectrum of **3** (DMSO-d_6, 500 MHz)

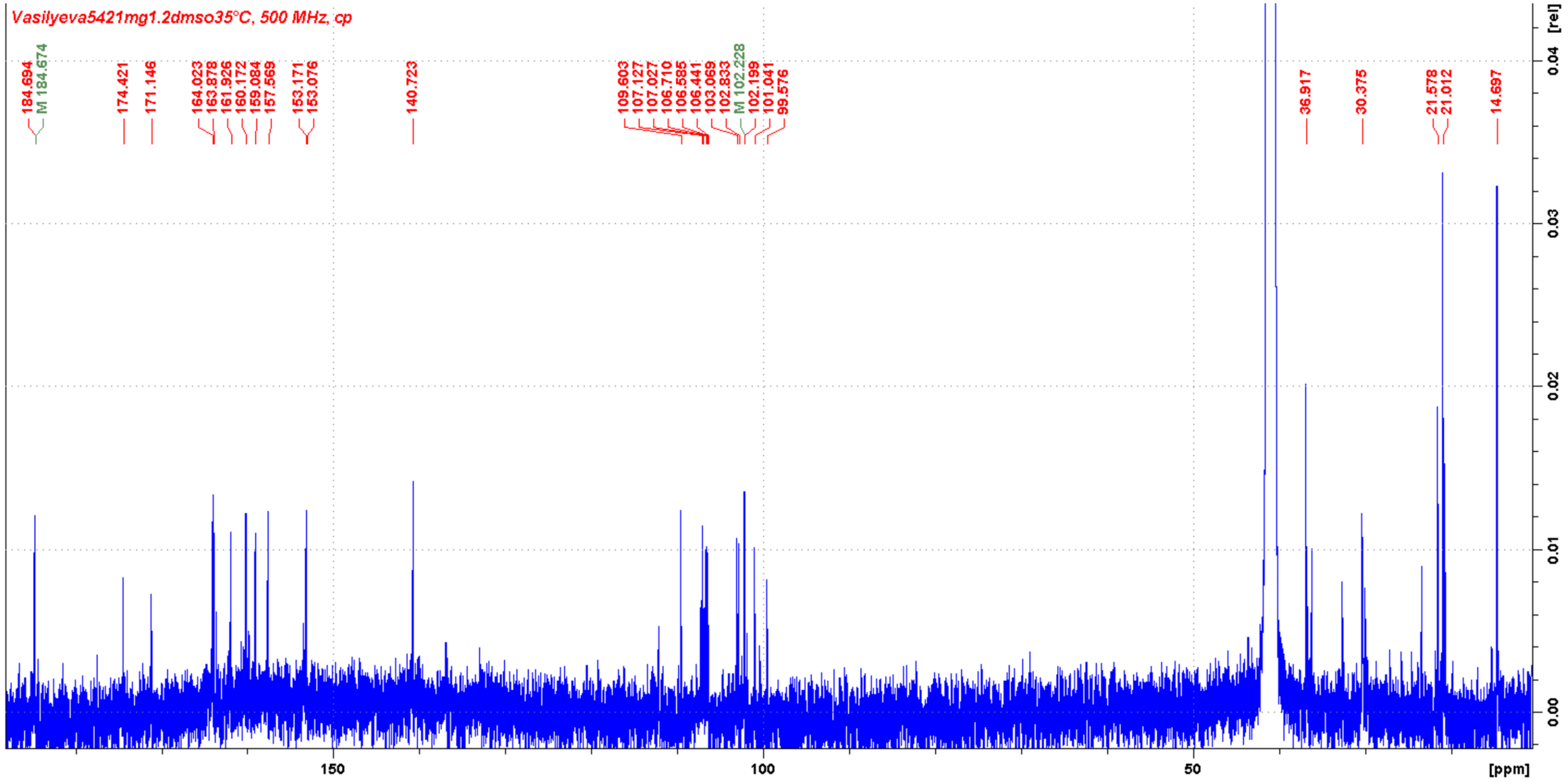

Figure S39. ^{13}C NMR spectrum of **3** (DMSO-d_6, 126 MHz)

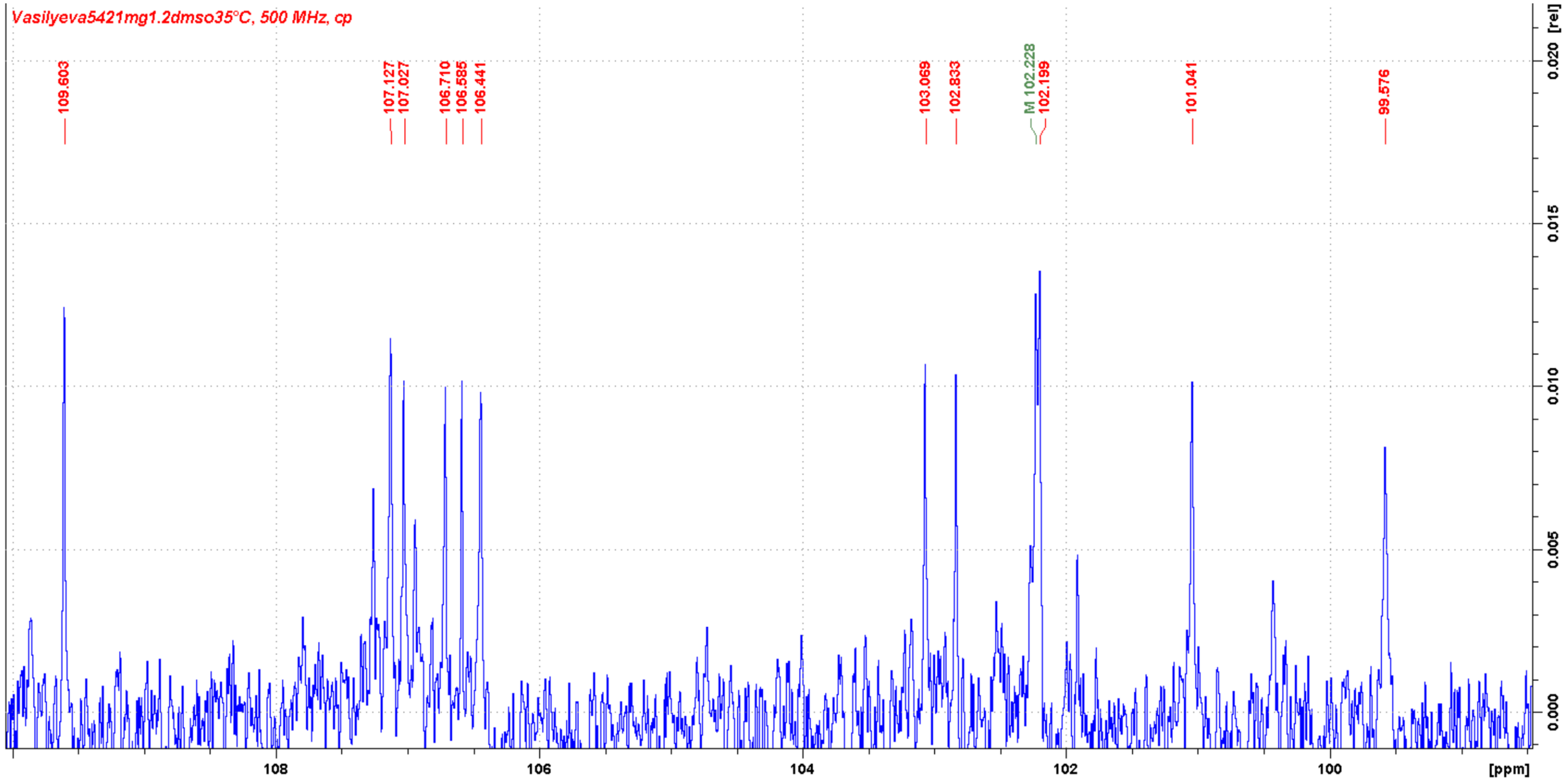

Figure S40. Enlarged fragment of ^{13}C NMR spectrum of **3** (DMSO-d_6, 126 MHz)

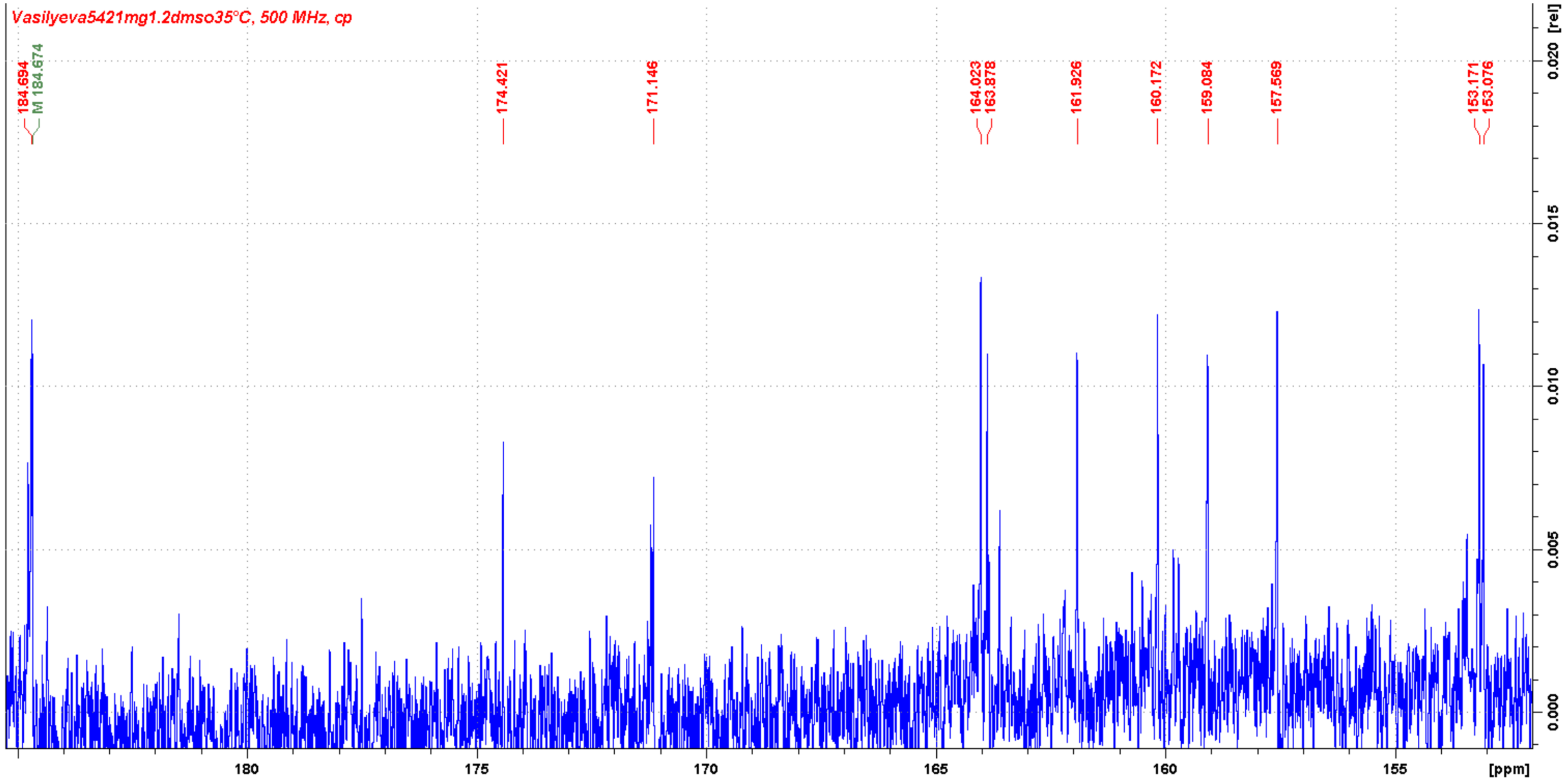

Figure S41. Enlarged fragment of ^{13}C NMR spectrum of **3** (DMSO-*d*$_6$, 126 MHz)

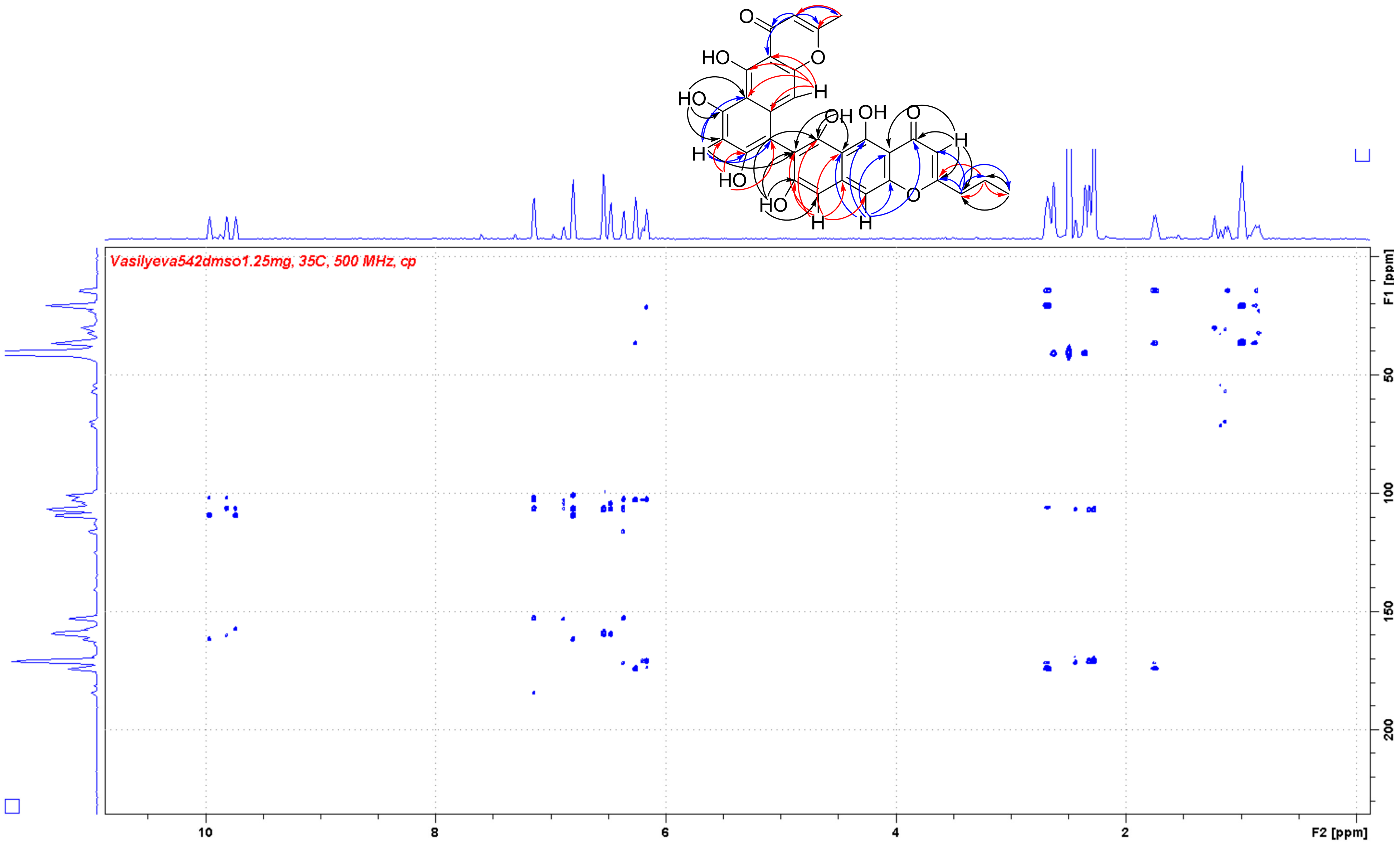

Figure S42. HMBC spectrum of **3**

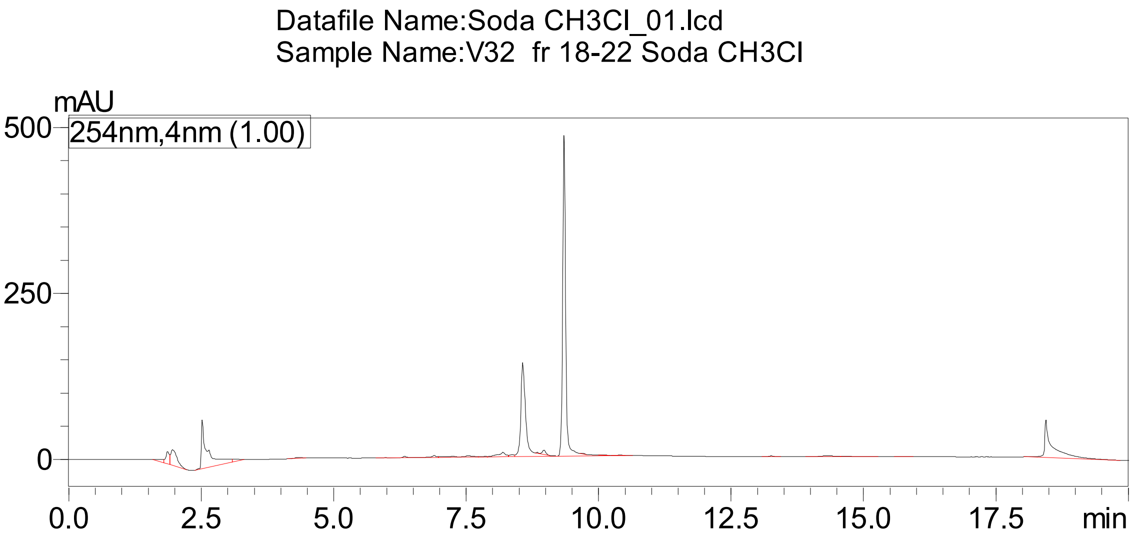

Figure S43. HPLC profile of compound **1** after purification using Na_2CO_3 – compound **4** was derived

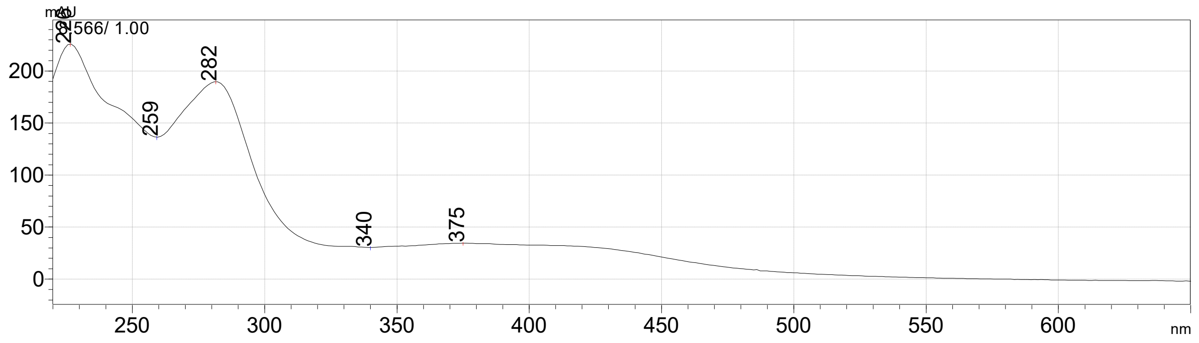

Figure S44. Absorption spectrum of compound **4** (DAD)

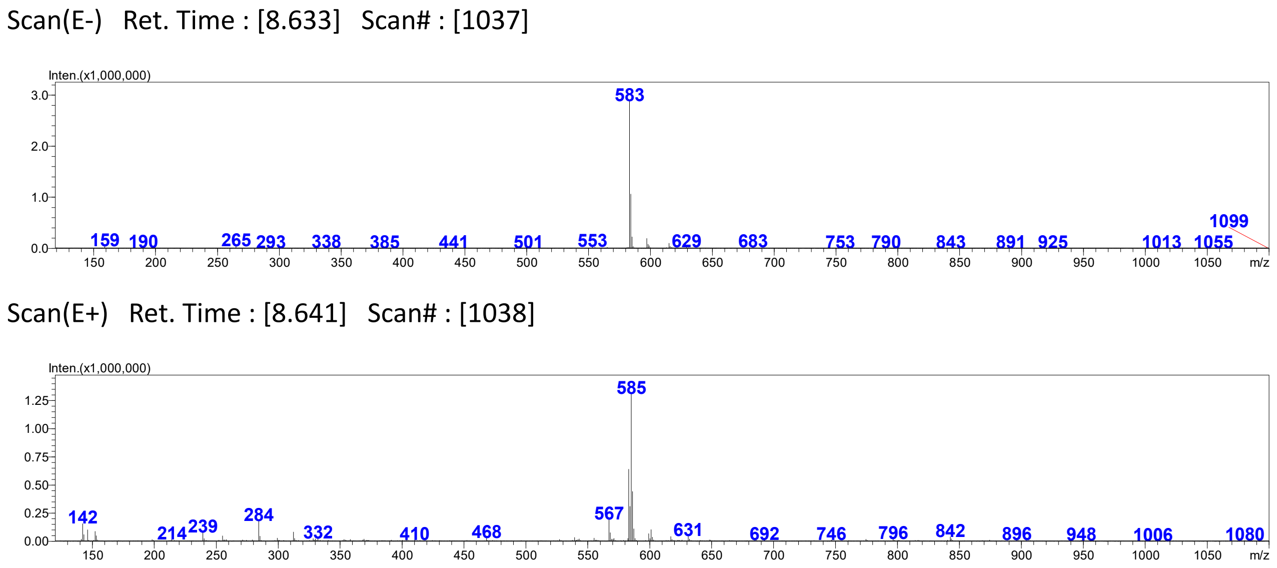

Figure S45. MS data of compound **4**

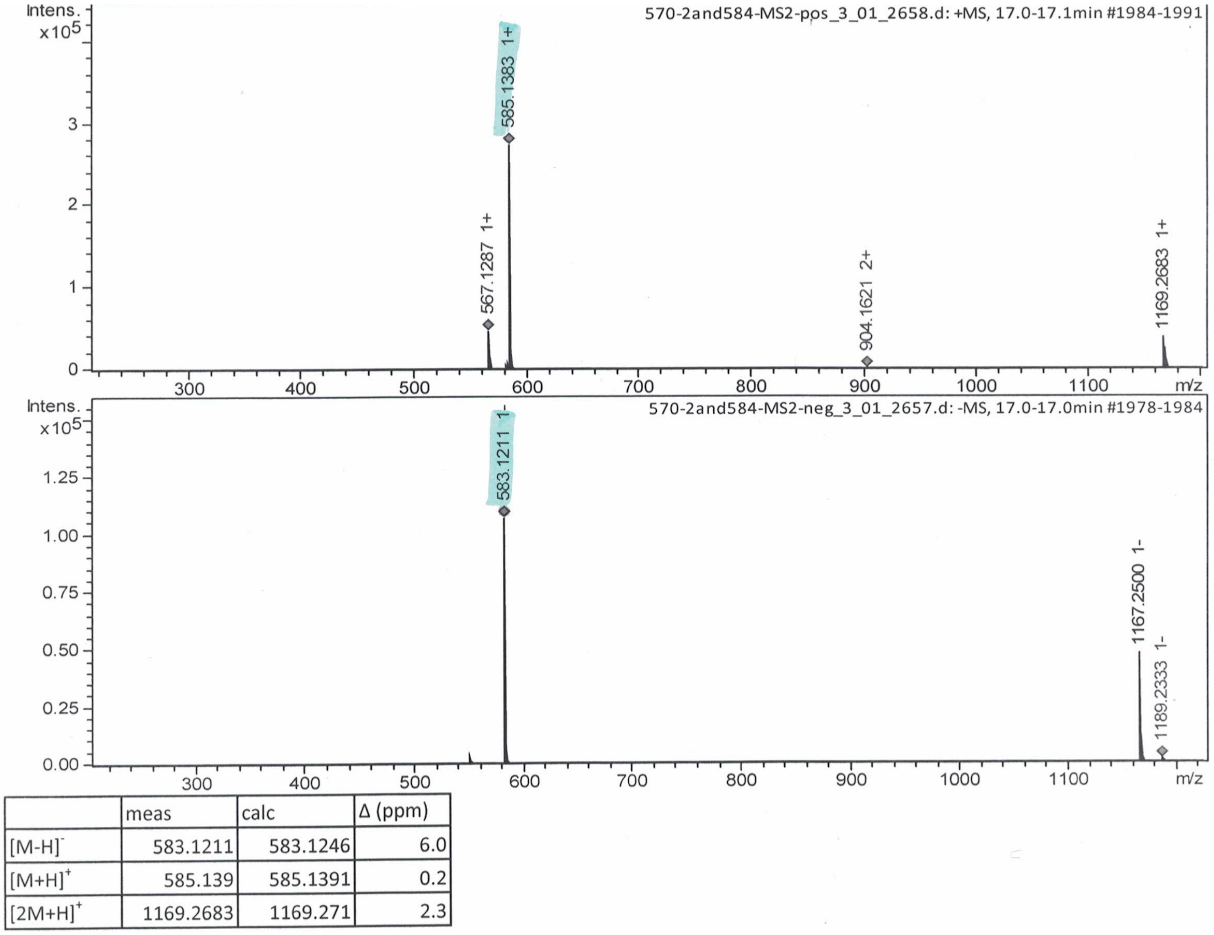

	meas	calc	Δ (ppm)
$[M-H]^-$	583.1211	583.1246	6.0
$[M+H]^+$	585.139	585.1391	0.2
$[2M+H]^+$	1169.2683	1169.271	2.3

Figure S46. HRESIMS data of compound **4**

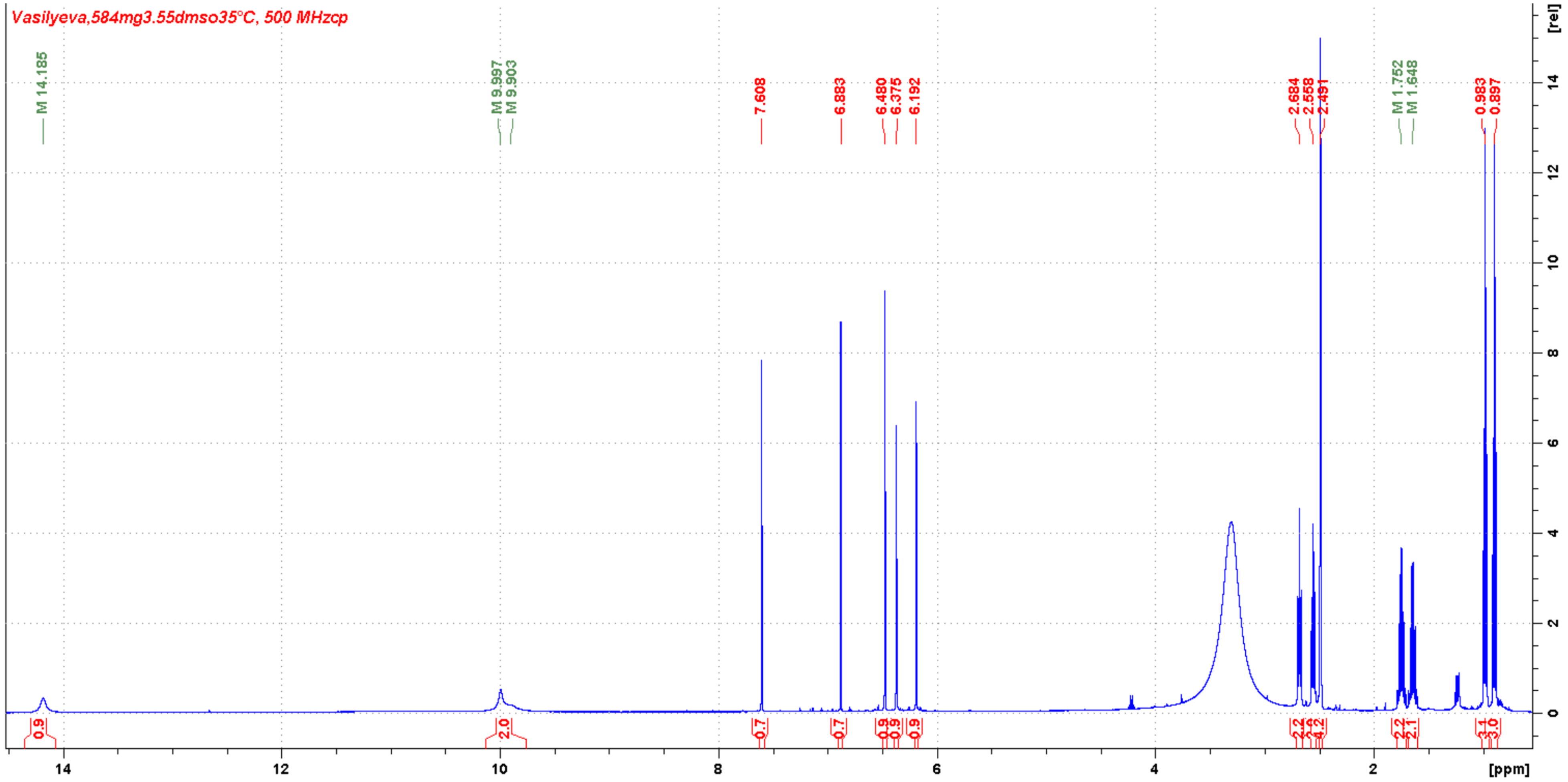

Figure S47. ^{1}H NMR spectrum of **4** (DMSO-d_6, 500 MHz)

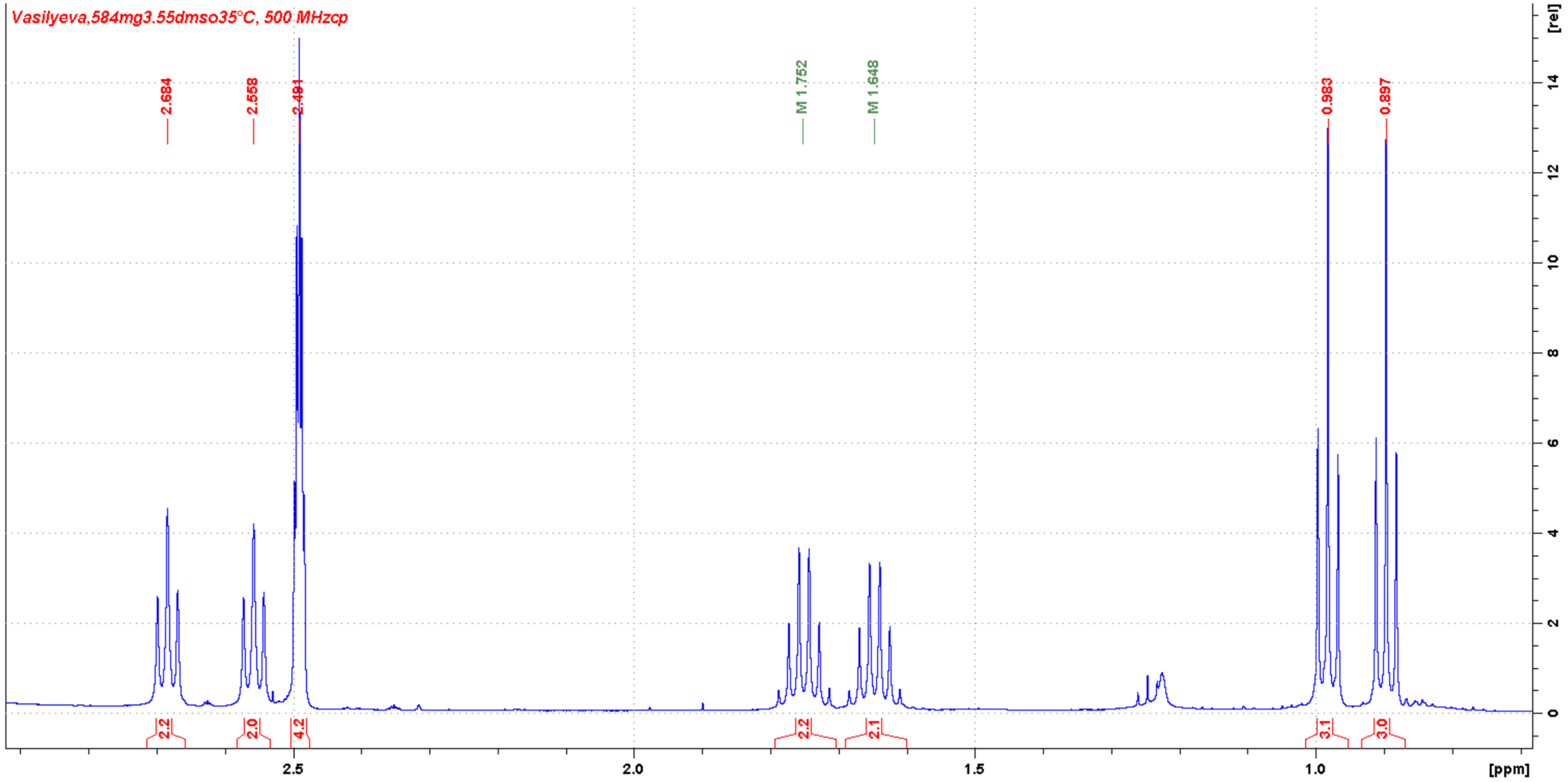

Figure S48. Enlarged fragment of ^{1}H NMR spectrum of **4** (DMSO-d_6, 500 MHz)

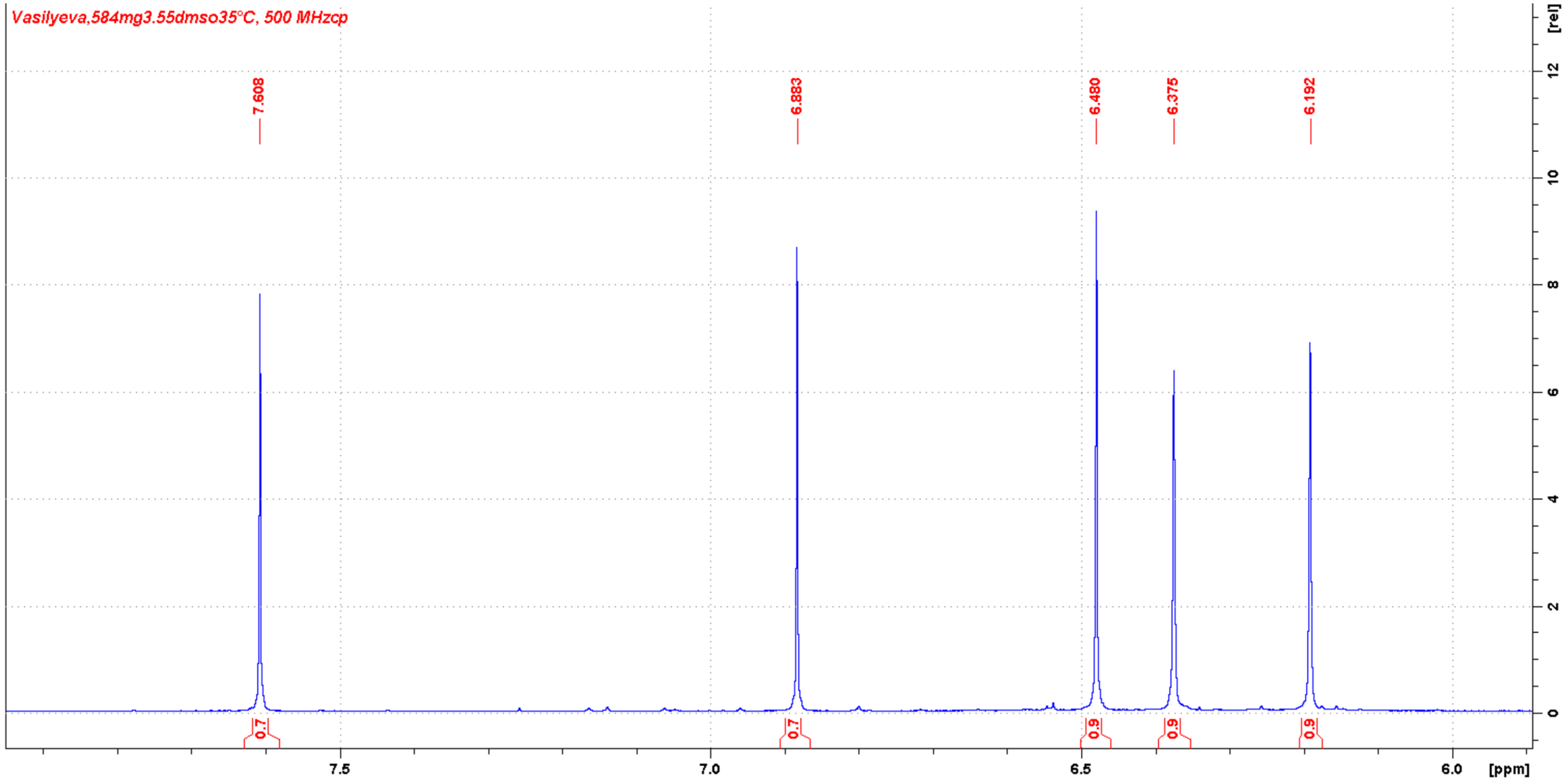

Figure S49. Enlarged fragment of ^{1}H NMR spectrum of **4** (DMSO-d_6, 500 MHz)

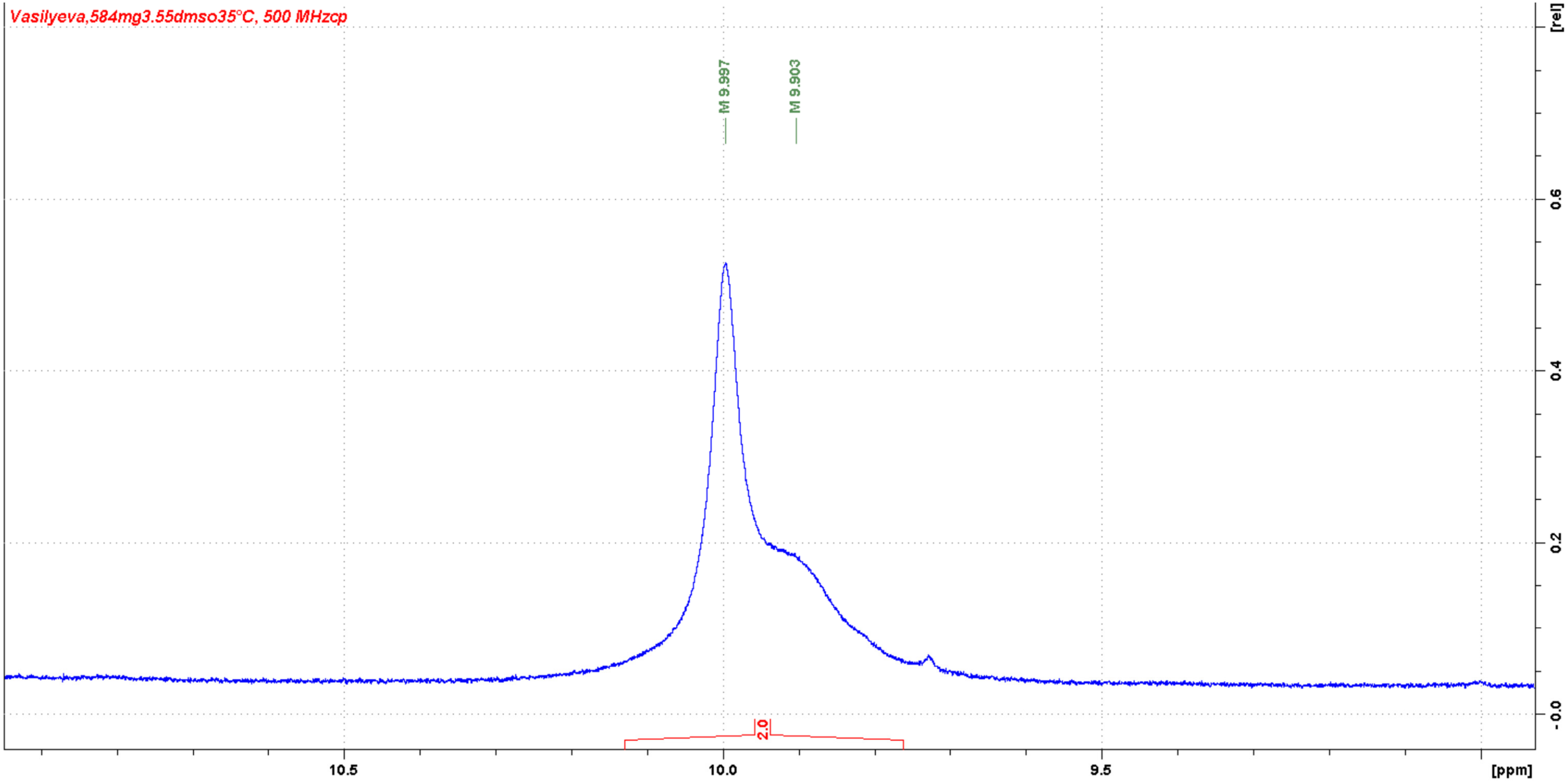

Figure S50. Enlarged fragment of ^{1}H NMR spectrum of **4** (DMSO-d_6, 500 MHz)

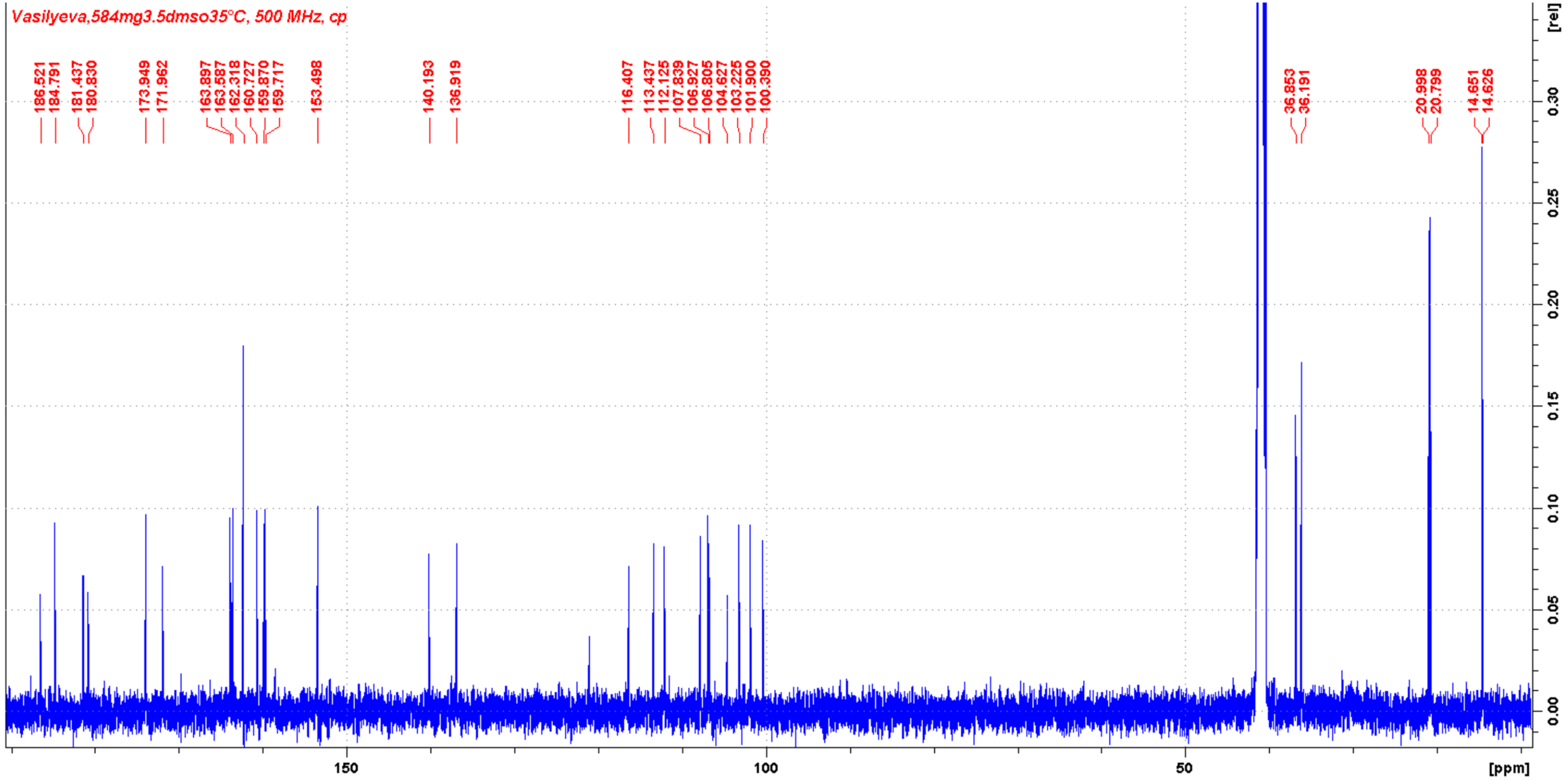

Figure S51. ^{13}C NMR spectrum of **4** (DMSO-d_6, 126 MHz)

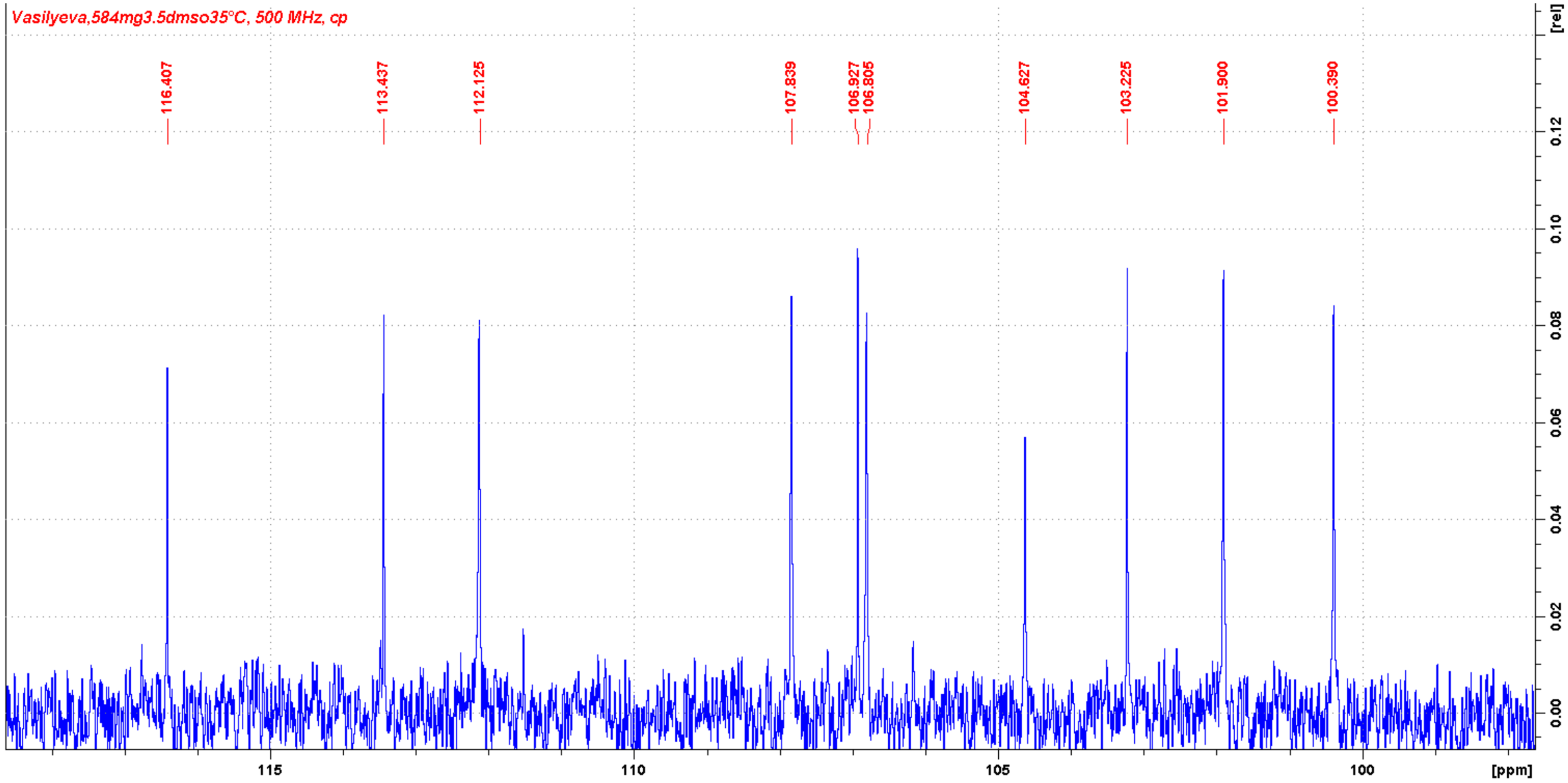

Figure S52. Enlarged fragment of ^{13}C NMR spectrum of **4** (DMSO-d_6, 126 MHz)

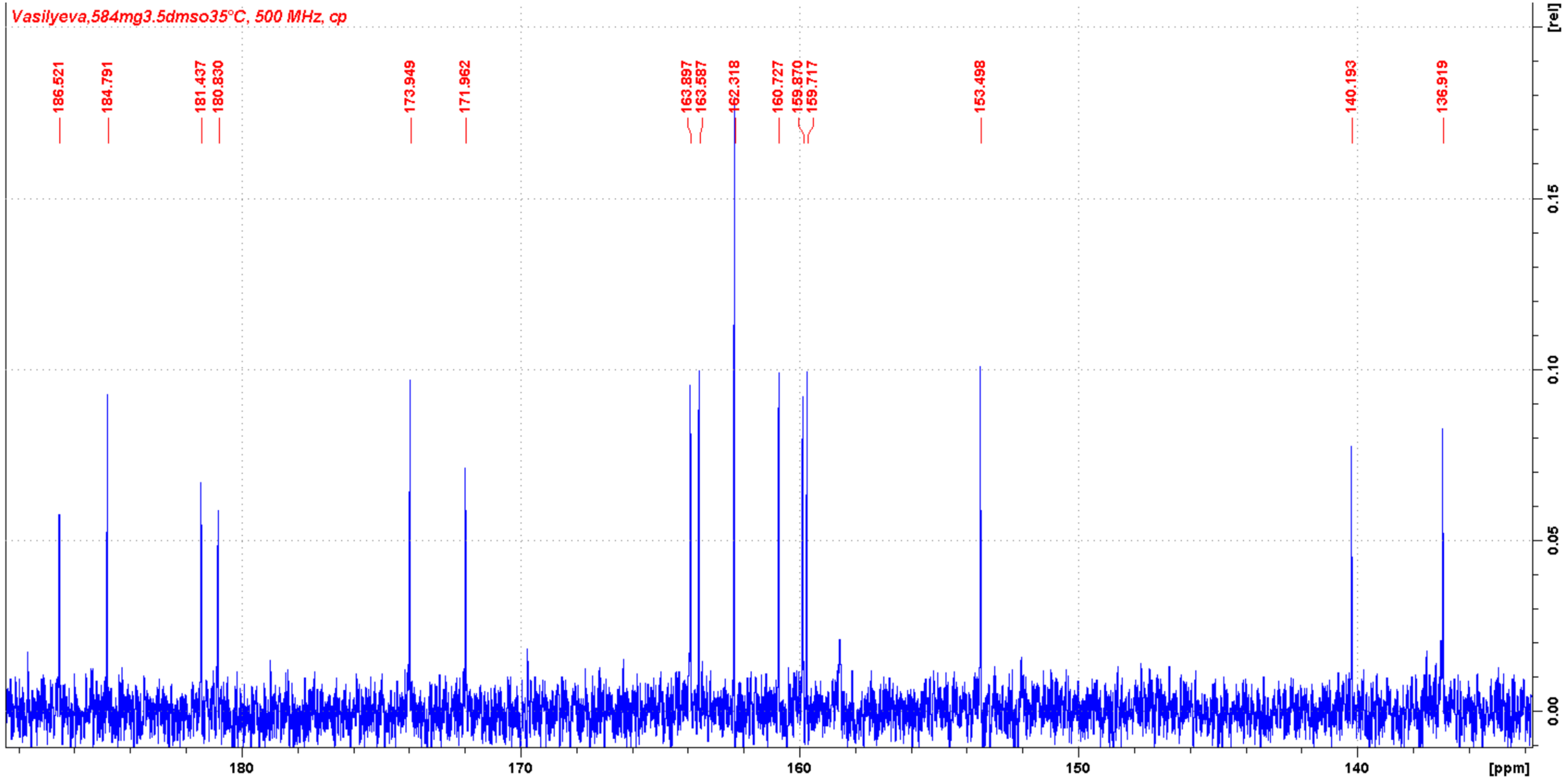

Figure S53. Enlarged fragment of ^{13}C NMR spectrum of **4** (DMSO-d_6, 126 MHz)

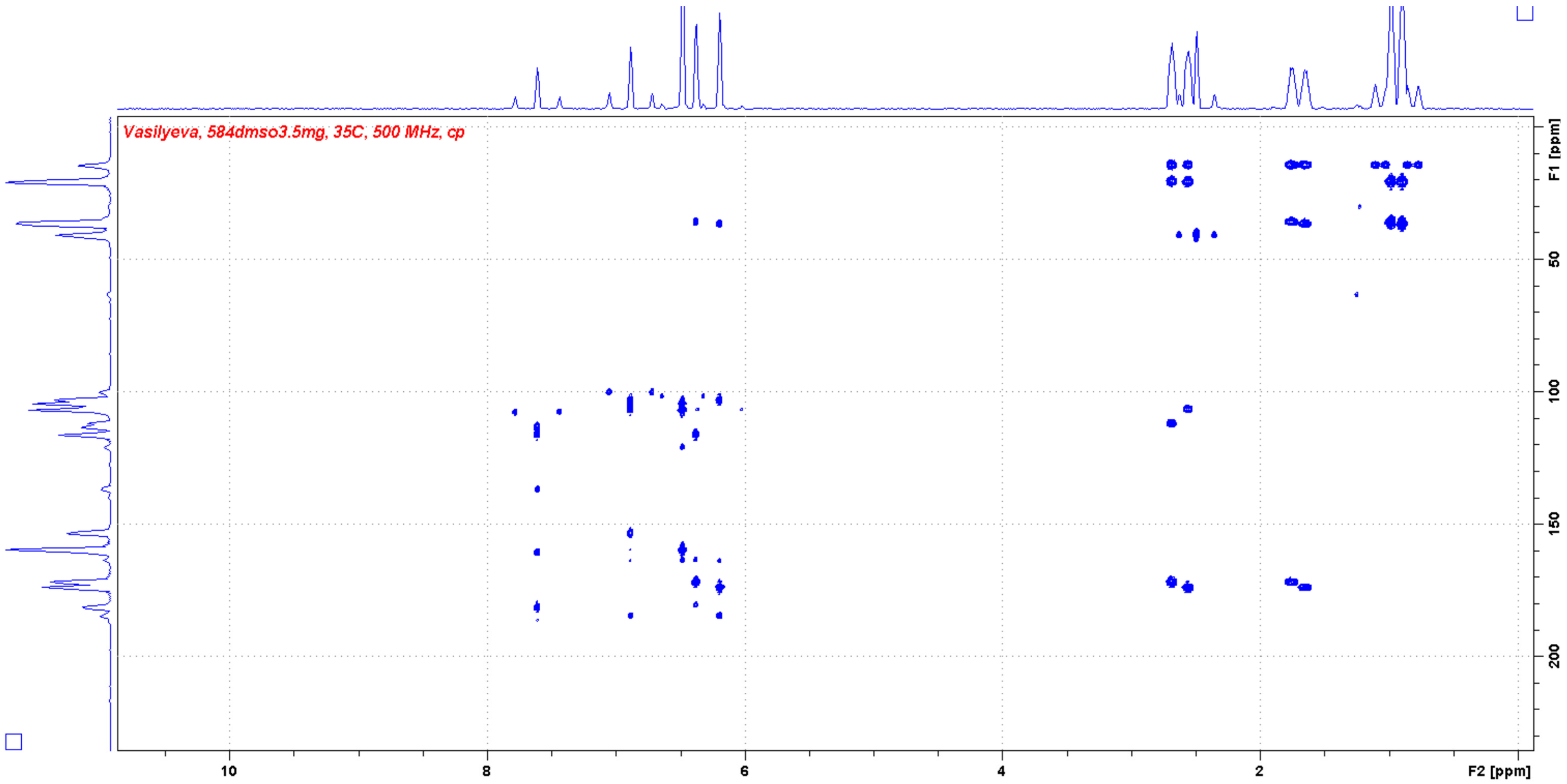

Figure S54. HMBC spectrum of **4** (DMSO-d_6, 126 MHz)

Investigation of interaction of compound 1 with recombinant cytochromes P450 using SPR analysis

The analysis was performed using the Biacore 8K SPR biosensor (Cytiva, USA). The resulting sensorgrams of interaction were processed using the Biacore Insight Evaluation software package Version 3.0.11.15423. For analysis, standard CM5 optical chips with carboxymethylated dextran on the gold surface (Cytiva, USA) were used. Ligands (molecules immobilized on the surface of the optical chip of the biosensor): recombinant cytochromes P450 of *Mycobacterium tuberculosis* (CYP51, CYP124, CYP125, CYP126, CYP142), *Candida albicans* (CYP51), *Candida glabrata* (CYP51), *Homo sapiens* (CYP51);

Activation of the surface of the working channel was carried out by injection of an activating mixture of 50% EDC / 50% NHS for 7 minutes at a flow rate of 5 μL/min. Injection of a solution of ligands (studied CYPs) in immobilization buffer (10 mM sodium acetate, pH 4.5 in the case of CYP51 *Candida albicans* and *Candida glabrata*; 10 mM sodium acetate, pH 5.0 in the case of all other CYPs) was carried out for 5 min at flow rate 5 μL/min. After this, the chip was incubated for 1 hour in a docked state in the "Stand By" device mode at a measuring cell temperature of 25°C, which led to the hydrolysis of unreacted activated dextran groups. The amount of the protein immobilized on the chip is given in the Table S4.

Table S4. The amount of the ligand immobilized on the chip. MT - *Mycobacterium tuberculosis*, human - *Homo sapiens*, Calb - *Candida albicans*, Cglab - *Candida glabrata*

Ligands	The amount of the ligand immobilized, RU
CYP51_MT	5499
CYP124_MT	3315
CYP125_MT	5730
CYP126_MT	4590
CYP142_MT	2445
CYP51_human	8286
CYP51_Calb	2603
CYP51_Cglab	4888

A 10 mM stock solution of compound **1** was prepared using dimethyl sulfoxide. Next, a 100 μM solution of the compound **1** was prepared using HBS-N buffer. Lower concentrations of **1** were prepared from a 100 μM HBS-N solution with a buffer containing 1% DMSO. HBS-N buffer with 1% DMSO was also used as a working buffer for the biosensor.

The analysis was carried out at a temperature of 25 °C. The channel of the measuring cell with an immobilized ligand was used as the experimental channel. A channel in which the ligand was not immobilized was used as a control channel to level the signal from the nonspecific interaction of the analyte protein with the surface of the biosensor chip. The resulting sensorgram was the difference between the signals from the channel with the immobilized ligand and the control empty channel. Compound **1** in the concentration range of 10-100 μM was injected over the surface of the chip with immobilized ligands for 210 s at a flow rate of 15 μL/min. The dissociation of complexes was recorded for 900 s at the same flow rate. Regeneration of the chip surface after injection of the analyte was carried out by double injection of a regenerating solution of 2 M NaCl, 0.5% CHAPS for 30 s at a flow rate of 30 μL/min.

The resulting sensorgrams were processed in the Biacore Insight Evaluation Program Version 3.0.11.15423 (Cytiva, USA). The two-state reaction model was chosen as a processing model corrected for the contribution of the refractive index (RI parameter, estimated individually for each sensorgram). Kinetic interaction parameters were calculated globally for the entire concentration range. The analysis results are shown in Table S5. Selection of the result of mathematical processing of sensorgrams was carried out on the basis of the minimum parameter chi2 (the standard deviation of the points of the calculated graph from the actual obtained sensorgram).

Table S5. Parameters of interaction of compound **1** with cytochromes of various organisms immobilized on the CM5 optical chip.

Ligand	ka1 (1/Mc x 10^{2})	kd1 (1/c x 10^{-2})	ka2 (1/c x 10^{-3})	kd2 (1/c x 10^{-4})	KD (мкМ)
CYP51_MT	1,27	2,21	1,97	12,5	67,3

CYP124_MT	0,021	1,14	4,56	6,80	707
CYP125_MT	1,11	2,32	2,29	8,18	55,3
CYP126_MT	0,654	1,86	2,85	7,71	60,5
CYP142_MT	0,137	1,70	4,64	6,99	163
CYP51_human	0,655	2,42	1,66	9,84	138
CYP51_Calb	0,378	6,82	4,31	6,51	237
CYP51_Cglab	0,606	2,07	2,21	8,36	93,9
